# Tracing Bai-Yue Ancestry in Aboriginal Li People on Hainan Island

**DOI:** 10.1093/molbev/msac210

**Published:** 2022-09-29

**Authors:** Hao Chen, Rong Lin, Yan Lu, Rui Zhang, Yang Gao, Yungang He, Shuhua Xu

**Affiliations:** Key Laboratory of Computational Biology, Shanghai Institute of Nutrition and Health, University of Chinese Academy of Sciences, Chinese Academy of Sciences, Shanghai 200031, China; Department of Biology, Hainan Medical University, Haikou 571199, Hainan, China; Center of Forensic Medicine of Hainan Medical University, Hainan Provincial Academician Workstation (Tropical Forensic Medicine), Hainan Provincial Tropical Forensic Engineering Research Center, Haikou 571199, Hainan, China; State Key Laboratory of Genetic Engineering, Center for Evolutionary Biology, Collaborative Innovation Center for Genetics and Development, School of Life Sciences, Fudan University, Shanghai 200438, China; Human Phenome Institute, Zhangjiang Fudan International Innovation Center, and Ministry of Education Key Laboratory of Contemporary Anthropology, Fudan University, Shanghai 201203, China; Key Laboratory of Computational Biology, Shanghai Institute of Nutrition and Health, University of Chinese Academy of Sciences, Chinese Academy of Sciences, Shanghai 200031, China; Human Phenome Institute, Zhangjiang Fudan International Innovation Center, and Ministry of Education Key Laboratory of Contemporary Anthropology, Fudan University, Shanghai 201203, China; Shanghai Fifth People’s Hospital, and Shanghai Key Laboratory of Medical Epigenetics, International Co-laboratory of Medical Epigenetics and Metabolism (Ministry of Science and Technology), Institutes of Biomedical Sciences, Fudan University, Shanghai 200032, China; State Key Laboratory of Genetic Engineering, Center for Evolutionary Biology, Collaborative Innovation Center for Genetics and Development, School of Life Sciences, Fudan University, Shanghai 200438, China; Human Phenome Institute, Zhangjiang Fudan International Innovation Center, and Ministry of Education Key Laboratory of Contemporary Anthropology, Fudan University, Shanghai 201203, China; Center for Excellence in Animal Evolution and Genetics, Chinese Academy of Sciences, Kunming 650223, China; Department of Liver Surgery and Transplantation Liver Cancer Institute, Zhongshan Hospital, Fudan University, Shanghai 200032, China; Jiangsu Key Laboratory of Phylogenomics and Comparative Genomics, School of Life Sciences, Jiangsu Normal University, Xuzhou 221116, China

**Keywords:** Li population, aboriginal people, local adaptation, Bai-Yue ancestry, Tai-Kadai language, genetic admixture

## Abstract

As the most prevalent aboriginal group on Hainan Island located between South China and the mainland of Southeast Asia, the Li people are believed to preserve some unique genetic information due to their isolated circumstances, although this has been largely uninvestigated. We performed the first whole-genome sequencing of 55 Hainan Li (HNL) individuals with high coverage (∼30–50×) to gain insight into their genetic history and potential adaptations. We identified the ancestry enriched in HNL (∼85%) is well preserved in present-day Tai-Kadai speakers residing in South China and North Vietnam, that is, Bai-Yue populations. A lack of admixture signature due to the geographical restriction exacerbated the bottleneck in the present-day HNL. The genetic divergence among Bai-Yue populations began ∼4,000–3,000 years ago when the proto-HNL underwent migration and the settling of Hainan Island. Finally, we identified signatures of positive selection in the HNL, some outstanding examples included *FADS1* and *FADS2* related to a diet rich in polyunsaturated fatty acids. In addition, we observed that malaria-driven selection had occurred in the HNL, with population-specific variants of malaria-related genes (e.g., *CR1*) present. Interestingly, HNL harbors a high prevalence of malaria leveraged gene variants related to hematopoietic function (e.g., *CD3G*) that may explain the high incidence of blood disorders such as B-cell lymphomas in the present-day HNL. The results have advanced our understanding of the genetic history of the Bai-Yue populations and have provided new insights into the adaptive scenarios of the Li people.

## Introduction

Hainan Island is located in southern China and is considered a critical site connecting the human populations of East Asia and Southeast Asia (Li, Li, Ou, et al. 2008; Li et al. 2013). Several archaeological relic sites discovered in Changjiang County of Hainan Province have indicated that the earliest modern human settlement on Hainan Island could date back to ∼20,000 years ago (ya) in the Paleolithic Age, and the unearthed stone implements signify a high similarity with cultures from mainland South China (Li, Li, Wang, et al. 2008). The frequent movements of East Asian and Southeast Asian populations on the mainland have facilitated their genetic admixture, and further increased genetic diversity and phenotypic affinities of populations involved in admixture (Lipson et al. 2018; McColl et al. 2018). In turn, human genetic diversity in the insular region is always distinguished from those in the continent due to the effect of geographical isolation (Matsunami et al. 2021), resulting in the unique and uniform genetic backgrounds of island aboriginal people. As a result, Hainan Island may harbor ancient footprints of East Asian and Southeast Asian populations regarding genetic origins and evolution. Overall, the specific circumstances could intensify the merits of present-day aboriginal people living on Hainan Island regarding the preservation of distinctive genetic patterns and undergoing characteristics of adaptive evolution, and thus such information would help to gain insight into the identification of genetic variants with large effects in traits related to adaptations.

As the dominant aboriginal people living on Hainan Island, the Li (also known as the Hlai) population in Hainan (HNL) is considered an ethnic group in China whose history is not well known. The Li nationality is officially recognized as one of the 55 Chinese ethnic minorities, and the “Li” in ancient Chinese refers to the ethnic minority living dispersedly in the mountainous areas of Hainan Island. The present-day HNL speaks the Hlai language that belongs to the Tai-Kadai (also known as Kra-Dai) language family, and the group inhabits mountainous areas in Central and South Hainan Island. The earliest historical record of HNL can be traced back to Shangshu ∼2,500 ya (Li 2006), and the records of Shiji in the Han dynasty ∼2,200 ya formally described the HNL as one lineage of ancient Bai-Yue (Wu 1997; Li 2006). The “Bai-Yue” in ancient Chinese refers to the “hundreds of tribes,” who are collectively known as ancient indigenous Tai-Kadai-speaking populations living in the present-day south of the Yangtze River to North Vietnam (Jin et al. 2001). Under the influences and constraints from surrounding populations throughout history, the populations derived from ancient Bai-Yue lineage have undergone different migration, admixture, and isolation, which shape the various present-day southern East Asians (EAS.South) and mainland Southeast Asians (MSEA).

Nonetheless, the genetic origin and population history of HNL remain debatable. One hypothesis proposes the HNL migrated from South China and are descendants of the ancient Bai-Yue lineage. For example, previous studies based on mitochondrial DNA (mtDNA) and SNP-array data showed that HNL presented close genetic affinities with mainland Tai-Kadai-speaking populations in South China (He et al. 2020; Mengge et al. 2020). An alternative hypothesis based on Y-chromosomal data proposed that the HNL originated from ancient migrants from Southeast Asia to East Asia ∼20,000 ya (Li, Li, Ou, et al. 2008). Moreover, another study applying Y-chromosomal analysis proposed that the lower genetic diversity of HNL at the paternal level probably resulted from a founder effect (Song et al. 2019). These studies suggest that HNL manifested a close genetic relationship with indigenous populations in South China, where the Bai-Yue ancestors were believed to be widely distributed, while also retaining a unique genetic background. However, previous studies of the HNL have focused on forensic characteristics or uniparental genetic markers (Li, Li, Ou, et al. 2008; Peng et al. 2011; Li et al. 2013; Fan et al. 2018; Song et al. 2019; Li et al. 2020; Mengge et al. 2020) and have therefore failed to portray the full picture of genetic history and adaptive evolution of HNL. In addition, due to the limited amount of genetic material, small sample size, and analytical approaches, conclusions drawn from previous studies are contradictory and may show bias concerning the fine-scale population history of HNL. Indeed, genetic studies of HNL remain largely unexplored, and fundamental questions remain unsolved, including (1) whether there is a Bai-Yue ancestry enriched in HNL and other indigenous populations living in present-day South China and North Vietnam; (2) when the HNL arrived at Hainan Island; (3) whether there is recent genetic admixture in HNL and when it began; (4) whether there was adaptive evolution of HNL attributed to the local environment of the isolated island.

To obtain explicit information concerning the genetic characterization of HNL, in the present study we sequenced whole genomes of 55 HNL individuals living in Central and South Hainan Island (supplementary fig. S1*A*, Supplementary Material online), the main settlement of the Li population. Analyzing the genetic data together with East Asian and Southeast Asian populations (supplementary table S2, Supplementary Material online), especially the populations of EAS.South and MSEA, we describe the population structure, demographic history, and local adaptations of the HNL. We provide new insights into the genetic history of populations from the Bai-Yue lineage, and the result will advance our understanding of human adaptive evolution in insular circumstances.

## Results

### Genetic Profile of HNL

**Fig. 1.**
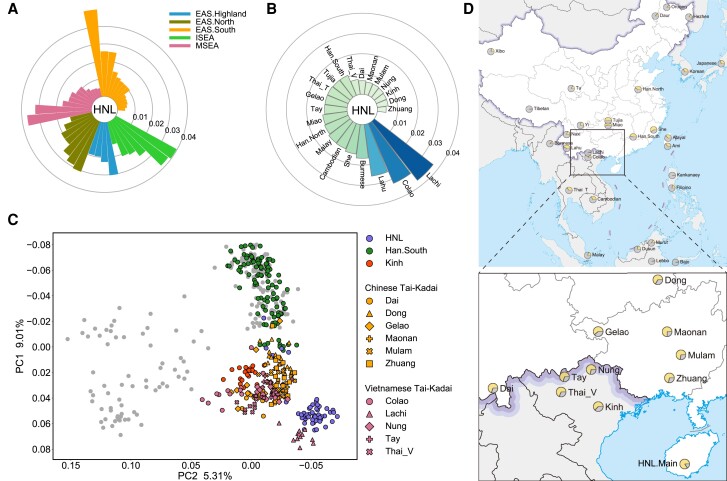
Genetic affinity and Bai-Yue ancestry profile of HNL with East Asian and Southeast Asian populations. (*A*) A fan-like chart showing genetic affinity measured by *F_ST_* between HNL and other East Asian and Southeast Asian populations. (*B*) A fan-like chart showing genetic affinity measured by *F_ST_* between HNL and populations from southern East Asia (except Ami and Atayal) and mainland Southeast Asia. (*C*) PCA of 53 unrelated HNL individuals with individuals of other southern East Asian and mainland Southeast Asian populations. Populations close to HNL clusters are colored and labeled on the PC plot. (*D*) Map visualization showing the proportion of Bai-Yue ancestry in East Asian and Southeast Asian populations, inferred by the *ADMIXTURE* analysis at *K* = 7. The boxed and enlarged part of the map represents the main distribution area of present-day Bai-Yue populations. EAS.Highland: East Asian highlanders; EAS.North: northern East Asians; EAS.South: southern East Asians; ISEA: island Southeast Asians; MSEA: mainland Southeast Asians.

Principal component analysis (PCA) in the context of global populations showed that HNL was located in the cluster of East Asians and Southeast Asians (supplementary fig. S2*A*, Supplementary Material online). In particular, HNL was placed together with EAS.South and MSEA (supplementary fig. S2*B*, Supplementary Material online), consistent with the result of genetic differentiation measured by unbiased *F_ST_* (fig. 1*A*). Notably, HNL was overall most closely related to the Tai-Kadai-speaking populations in South China and North Vietnam, such as Zhuang (*F_ST_* = 0.0043), Dong (*F_ST_* = 0.0054), and Nung (*F_ST_* = 0.0058), as well to the Austroasiatic-speaking Kinh (*F_ST_* = 0.0055; fig. 1*B* and supplementary fig. S3, Supplementary Material online). This pattern was also supported by the outgroup *f*_3_ statistics (supplementary fig. S2*C*, Supplementary Material online). In addition, the PCA with EAS.South and MSEA showed that there was a substructure within the HNL (fig. 1*C* and supplementary fig. S4*A*, Supplementary Material online). The major cluster of HNL was close to Chinese and Vietnamese Tai-Kadai speakers, and another cluster of five individuals was clustered with Han and other EAS.South (fig. 1*C* and supplementary fig. S4*A*, Supplementary Material online). We also observed the results of both *F_ST_* (fig. 1*B* and supplementary fig. S3, Supplementary Material online) and three-dimensional PCA (supplementary fig. S4*B*, Supplementary Material online) showed Tai-Kadai-related Colao and Lachi in North Vietnam presented large genetic differences from other populations, probably suggesting there are additional genetic components in these two populations. The more detailed PC plots indicated that the major HNL cluster could be distinguished from the other Tai-Kadai-speaking populations and Kinh after removing outliers Colao and Lachi (supplementary fig. S4*C*, Supplementary Material online), while the minor HNL cluster remained to be clustered with the Han and EAS.South (supplementary fig. S4*D*, Supplementary Material online). Here, we defined HNL individuals from the major cluster as HNL.Main and the remaining five HNL individuals admixed with the cluster of Han and EAS.South as HNL.Admixed. The respective *F_ST_* (supplementary fig. S5, Supplementary Material online) and outgroup *f*_3_ statistics (supplementary fig. S6, Supplementary Material online) of HNL.Main and HNL.Admixed also produced similar results as for the PCA (fig. 1*C* and supplementary fig. S4, Supplementary Material online). We also confirmed that the HNL substructure was not biased by the sampling locations (Fisher’s exact test, *P* > 0.05; supplementary fig. S7, Supplementary Material online).

To dissect the ancestral composition of HNL, we further performed global ancestry inference using *ADMIXTURE* (Alexander et al. 2009). The *ADMIXTURE* results showed that HNL.Main harbored an ancestral component in the highest frequency across East Asia and Southeast Asia assuming *K* ≥ 6 (supplementary fig. S8, Supplementary Material online). Excepting Colao and Lachi, the ancestry dominated in HNL.Main (84.32 ± 3.41%) was enriched in Kinh and other Tai-Kadai-speaking populations in South China and North Vietnam at *K* = 7 (fig. 1*D*). A similar pattern was retained even with larger *K* (supplementary fig. S8, Supplementary Material online), suggesting a common genetic origin of these populations. In this study, we defined the ancestry substantially enriched in HNL.Main as Bai-Yue ancestry. HNL and other populations with relatively enriched Bai-Yue ancestry (>60%) at *K* = 7 who do not form distinct genetic components at larger *K* were defined as the Bai-Yue populations. These include Dai, Dong, Gelao, Maonan, Mulam, and Zhuang from South China, and Kinh, Nung, Tay, and Thai_V from North Vietnam (fig. 1*D*). We also distinguished these populations from HNL as mainland Bai-Yue populations, since they all live on the mainland of East Asia and Southeast Asia. The phylogenetic tree constructed from pairwise *F_ST_* also showed that HNL and these mainland Bai-Yue populations are located on the same branch (supplementary figs. S3*B* and S5*D*, Supplementary Material online).

To elucidate the paternal and maternal structures of the HNL, we also identified their non-recombining Y-chromosome (NRY) and mtDNA haplogroups (supplementary table S3, Supplementary Material online) and collected haplogroup data of East Asian and Southeast Asian populations for a comparative analysis (see Materials and Methods, supplementary tables S4 and S5, Supplementary Material online). We found that the HNL harbored the second-lowest NRY haplogroup diversity among all populations due to the high proportion of NRY haplogroup O-M175 (97.96%), only slightly higher than that of the Taiwan aboriginal Atayal (supplementary fig. S9*A* and *B*, Supplementary Material online). The main haplogroup O-M175 in HNL was dominated by sublineages O1a-M119 (25.17%), O1b1-F2320 (61.9%), and O2-M122 (10.88%). Among these haplogroups, O1b1-F2320 was prevalent in Bai-Yue populations, and had the highest proportion in HNL (supplementary fig. S9*C*and table S4, Supplementary Material online). The PCA based on NRY haplogroup frequency also illustrated that HNL had a close relationship with Bai-Yue populations such as Dai and Zhuang (supplementary fig. S9*D*, Supplementary Material online). As for the mtDNA haplogroup results, HNL showed much higher mtDNA haplogroup diversity than NRY, and the main mtDNA haplogroups of HNL, B (20.83%), F (23.61%), and M7 (27.78%), are widely distributed in EAS.South (supplementary fig. S10*A*, *B*and table S5, Supplementary Material online). Moreover, the PCA based on mtDNA haplogroup frequency also showed that the haplogroup pattern of the Bai-Yue populations including Dai and Kinh was similar to that of the HNL (supplementary fig. S10*C*, Supplementary Material online). In summary, consistent with the PCA results from the autosomal data, Bai-Yue populations showed a close genetic relationship at both the paternal and maternal levels, and HNL retained the highest proportion of Bai-Yue-dominated lineages.

### Genomic Diversity and Genetic Ancestry of HNL

As revealed by the *ADMIXTURE* analysis, the Bai-Yue ancestry in HNL.Main is genetically homogeneous with low variation, indicating strong drift due to isolation. To measure the population inbreeding of HNL, we calculated the runs of homozygosity (ROH) for HNL and compared this with other East Asian populations in the next-generation sequencing (NGS) panel (see Materials and Methods). The HNL showed larger numbers and longer average lengths of medium (0.5–1 Mb) and long ROH (>1 Mb) than other East Asian populations (supplementary fig. S11, Supplementary Material online), supporting the hypothesis that HNL was more isolated from having lived on the island.

**Fig. 2.**
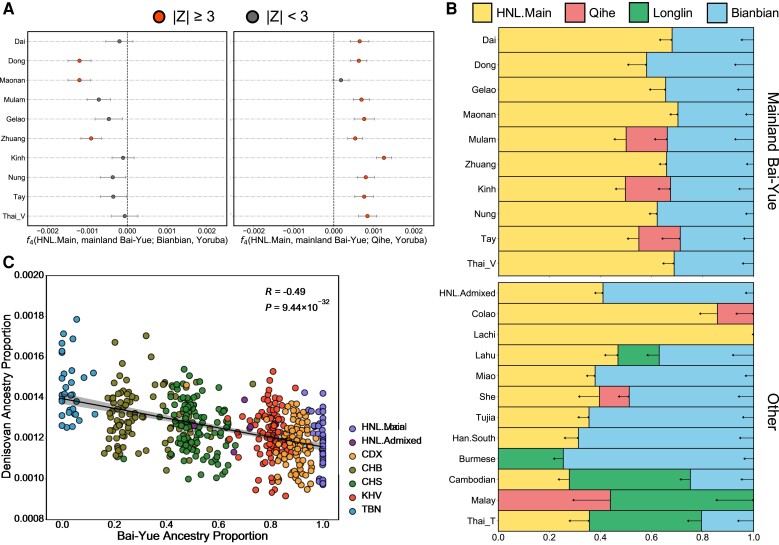
Genetic characterization of HNL based on comparison with other populations. (*A*) *f_4_* statistics in the form of *f_4_*(HNL.Main, mainland Bai-Yue groups; Bianbian, Yoruba) and *f_4_*(HNL.Main, mainland Bai-Yue groups; Qihe, Yoruba) testing the HNL’s genetic connections with ancient northern and southern East Asian ancestries compared with the mainland Bai-Yue populations. (*B*) *qpAdm*-based admixture models testing admixture for the southern East Asian and mainland Southeast Asian populations. A best-fitting model with the largest *P*-value is presented for each target population. The horizontal arrows represent the standard deviations for coefficients of ancestry sources. (*C*) Correlation between Bai-Yue ancestry proportion and Denisovan ancestry proportion in HNL and mainland East Asian populations. The shadow region indicates the 95% CI for the regression fit. CDX: Chinese Dai in Xishuangbanna, China; CHB: Han Chinese in Beijing, China; CHS: Han Chinese South; KHV: Kinh in Ho Chi Minh City, Vietnam; TBN: Tibetan.

We further calculated the *f*_3_ statistics in the form of *f*_3_(X, Y; HNL.Main), with X and Y as all the possible population combinations of East Asian and Southeast Asian populations to test for potential admixture in HNL.Main. We found no evident admixture signal can be detected with *f*_3_ tests since all *f*_3_ values were positive with *Z* values >10 (supplementary table S6, Supplementary Material online). We further calculated the admixture *f*_3_ in the form of *f*_3_(Bai-Yue groups, Han; HNL.Main) and *f*_3_(HNL.Main, Han; Bai-Yue groups). We observed *f*_3_(HNL.Main, Han; Bai-Yue groups) were consistently positive, whereas *f*_3_(Bai-Yue groups, Han; HNL.Main) show negative values for HNL.Admixed, Dong, Zhuang, and Kinh (supplementary fig. S12*A*, Supplementary Material online). These results suggest that admixture evidence was found in HNL.Admixed and another three mainland Bai-Yue populations, but was lacking in the HNL.Main. We alternatively employed *GLOBETROTTER* (Hellenthal et al. 2014) to detect plausible ancestral sources for HNL.Main and mainland Bai-Yue populations from multiple East Asian and Southeast Asian surrogates. The best-guess conclusion for admixture in the HNL.Main and Thai_V was “uncertain,” whereas potential admixture events were detected in other Bai-Yue populations (supplementary table S7, Supplementary Material online), suggesting less likely admixture events occurred in HNL.Main. To further test whether HNL is the best representation of a Bai-Yue ancestry found in present-day Bai-Yue populations, we introduced two ancient individuals, the Bianbian representing an ancient northern East Asian ancestry and the Qihe representing an ancient southern East Asian ancestry (Yang et al. 2020), and used *f*_4_ statistics in the form of *f*_4_(HNL.Main, mainland Bai-Yue groups; Bianbian/Qihe, Yoruba) to evaluate their genetic connections with ancient ancestries. The result illustrated the *f*_4_ values were consistently negative for Bianbian and positive for Qihe, which indicates HNL.Main show closer genetic connections with ancient southern East Asian ancestry than with mainland Bai-Yue populations (fig. 2*A*). We further introduced additional ancient ancestries from Guangxi of South China and applied *qpAdm*-based mixture models (Patterson et al. 2012) to characterize genetic ancestry components of present-day HNL and other Bai-Yue populations (see Materials and Methods, supplementary table S8, Supplementary Material online). We observed that HNL.Main harbored higher ancient southern ancestry (LadaKH01 + Qihe) but lower ancient northern ancestry (Bianbian) than other Bai-Yue populations. In addition, we also found that HNL.Main showed a higher proportion of Qihe ancestry, an ancestry related to that found in Austronesians (Yang et al. 2020), than other mainland Bai-Yue populations (supplementary fig. S12*B*, Supplementary Material online). This is consistent with our result of *f*_4_(HNL.Main, mainland Bai-Yue groups; Qihe, Yoruba) as well as a previous study that illustrated the Li population shows the highest ancestry proportion of Liangdao hunter-gatherer among Tai-Kadai speakers (Wang, Yeh, et al. 2021). We also computed the *f*_4_ statistics in the form *f*_4_(mainland Bai-Yue groups, X; HNL.Main, Yoruba), where X is other present-day East Asians and Southeast Asians, to investigate whether HNL showed different affinities with East Asians or Southeast Asians compared with other mainland Bai-Yue populations. We found that HNL showed a closer genetic affinity with isolated Austronesian populations that harbor more divergent ancestry, such as the Ami, Atayal, and Kankanaey (Lipson et al. 2014; Morseburg et al. 2016; Skoglund et al. 2016), than with the mainland Bai-Yue populations (supplementary fig. S13, Supplementary Material online), suggesting HNL could be a present-day Tai-Kadai-speaking population who is closer to the Austronesian-related ancestry. Overall, these results suggest that lower gene flow occurred in HNL because of the isolated circumstances; this may have helped to retain the genetic characteristics of HNL’s genome and to be representative of Bai-Yue ancestry.

As shown in the *ADMIXTURE* results, Bai-Yue ancestry was widely distributed in EAS.South and MSEA. We thus compared ancestry sharing between HNL and other EAS.South and MSEA based on identity by descents (IBDs). We found HNL.Main, Gelao, and Tay showed elevated levels of within-population IBD sharing compared with other Bai-Yue populations (supplementary fig. S14*A*, Supplementary Material online). In addition, between-population IBD sharing showed that HNL shared more IBD segments with mainland Bai-Yue populations than other EAS.South and MSEA (supplementary fig. S14, Supplementary Material online), suggesting the common ancestor sharing of Bai-Yue populations. Given the homogeneous genetic makeup of HNL.Main and better representativeness of Bai-Yue ancestry, we thus speculated that HNL.Main can be treated as an ancestral population of Bai-Yue lineage to test admixture induced by Bai-Yue ancestry in EAS.South and MSEA. We applied *qpAdm*-based admixture modeling and used HNL.Main as well as three ancient individuals with deep ancient ancestry (Bianbian, Qihe, and Longlin) as sources to estimate the corresponding ancestry coefficients of EAS.South and MSEA (see Materials and Methods, supplementary table S8, Supplementary Material online). The result showed the ancestry source of HNL.Main was utilized in the best-fitting models of EAS.South and MSEA except for Burmese and Malay (fig. 2*B*). In addition, we also observed that HNL.Main and Bianbian were modeled as two main ancestry sources of HNL.Admixed (fig. 2*B*). We then applied *RFMix* and used HNL.Main and present-day Han as ancestral populations to infer the local ancestry of HNL.Admixed (supplementary fig. S15, Supplementary Material online). The results showed that HNL.Admixed harbored 43.44% (±4.19%) and 56.56% (±4.19%) ancestral components of HNL.Main and Han, respectively (supplementary fig. S15, Supplementary Material online). We also applied *MultiWaver* to estimate the admixture time between HNL.Main and Han was ∼2,000 ya (82 generations with a generation time of 25 years).

Lastly, to test whether the Bai-Yue ancestry enriched in HNL was derived from archaic hominins, we identified archaic introgression segments in HNL and compared them with that in other mainland East Asian populations in the NGS panel (supplementary fig. S16, Supplementary Material online). We found that HNL harbored relatively fewer Denisovan archaic variants, and the Denisovan ancestry proportion in mainland East Asian populations was negatively correlated with the Bai-Yue ancestry (*R* = −0.49, *P* = 9.44 × 10^−32^) (fig. 2*C* and supplementary fig. S17, Supplementary Material online). Although the Denisovan ancestry was relatively lower in HNL, we identified three archaic introgression segments enriched in HNL but at relatively lower frequency in other populations (supplementary fig. S18, Supplementary Material online). Interestingly, among these archaic introgression signals, the involved gene *NPHP3-AS1* and the hypothetical gene *BC039487* were both reported to be associated with the age at menarche in previous genome-wide association studies (GWAS; Perry et al. 2009; Pickrell et al. 2016; Tachmazidou et al. 2017). These results suggest that Denisovans had less connection with southern East Asian populations of Bai-Yue ancestry, although relatively unique Denisovan sequences were identified in Bai-Yue populations.

### Genetic Origin and Population History

Given the genetically isolated ancestry identified in HNL, we also analyzed ancient DNA (aDNA) data that consist of ancient individuals of EAS.South and MSEA (see Materials and Methods, supplementary table S9, Supplementary Material online) to investigate the homogeneous Bai-Yue ancestry in HNL from ancient individuals with a wide time range. We first projected these ancient individuals onto the PCA of present-day East Asian and Southeast Asian populations and found that five ancient individuals from Guangxi in a historical era were placed with Bai-Yue populations (supplementary fig. S19, Supplementary Material online). In particular, three ancient Guangxi individuals ∼1,500 ya were placed with HNL.Main, and the other two Guangxi individuals ∼500 ya were closer to mainland Bai-Yue populations (supplementary fig. S19, Supplementary Material online). The *ADMIXTURE* analysis with aDNA data at *K* = 9 also illustrated the three ancient individuals ∼1,500 ya shared an ancestral component with Bai-Yue populations (supplementary fig. S20, Supplementary Material online). However, the results of outgroup *f*_3_ statistics in the form of *f*_3_(ancient individuals, present-day populations; Yoruba) in the context of EAS.South and MSEA showed that the five ancient individuals from Guangxi ∼1,500 and ∼500 ya presented a close genetic affinity with mainland Tai-Kadai speakers instead of HNL.Main (supplementary fig. S21, Supplementary Material online). In addition, HNL.Main showed closer genetic connections with several ancient individuals in Vietnam and Fujian, China ∼4,000 ya compared with other EAS.South and MSEA (supplementary fig. S21, Supplementary Material online). These results possibly suggest the genetic drift of HNL induced by isolation occurred earlier than 1,500 ya.

**Fig. 3.**
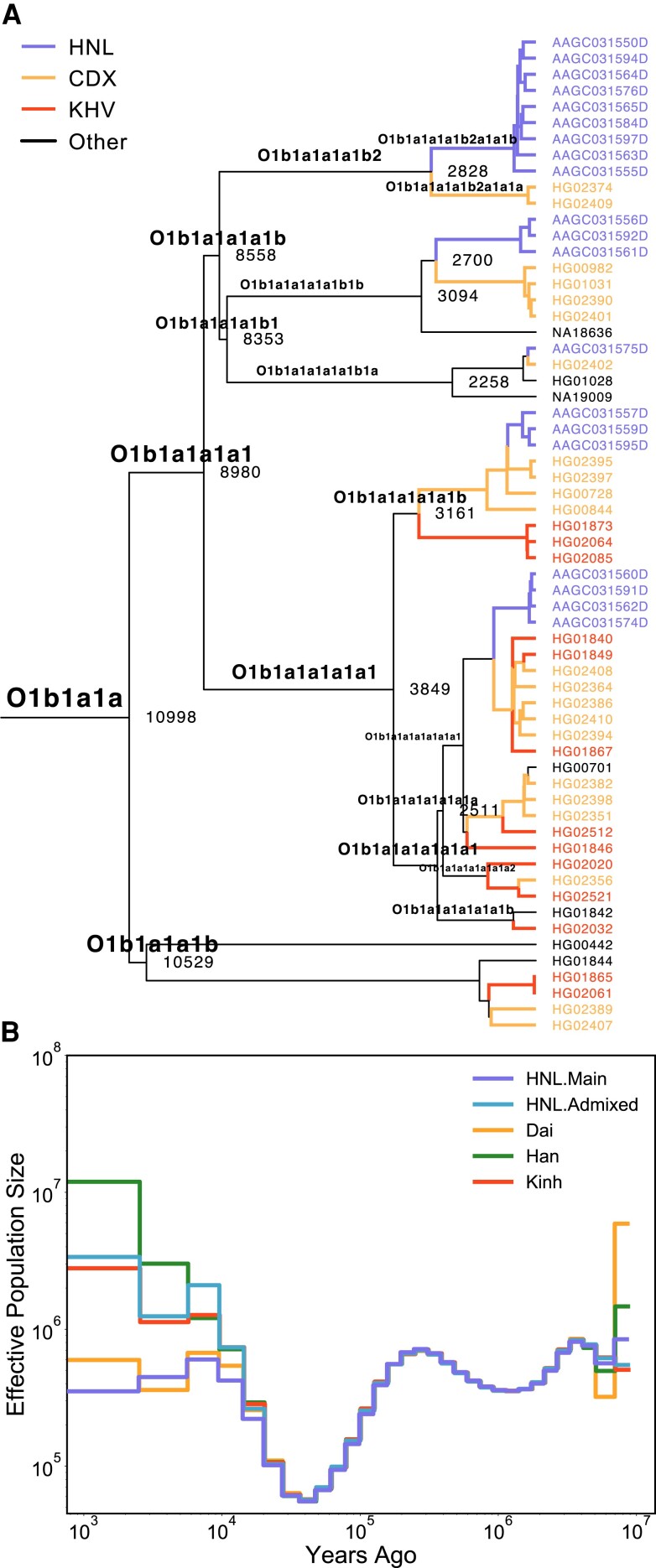
Genetic history of HNL with Bai-Yue lineage. (*A*) Y-chromosomal phylogeny of O1b1a1*a* (O-M95) with sublineages dominated by Bai-Yue populations. Numbers at each node represent coalescence dates in years. (*B*) Estimated historical effective population size for HNL, mainland Bai-Yue populations (Dai and Kinh), and Han. Estimates were scaled by an autosomal mutation rate of 1.25 × 10^−8^ per base pair per generation and 25 years per generation. CDX: Chinese Dai in Xishuangbanna, China; KHV: Kinh in Ho Chi Minh City, Vietnam.

In previous NRY haplogroup analysis, we found that O1b1a1a (O-M95) was a prevalent NRY haplogroup in HNL (supplementary table S3, Supplementary Material online), possibly suggesting the formation of the Bai-Yue lineage. To explore the potential genetic origin of the Bai-Yue lineage, we constructed a phylogeny based on Y-chromosomal sequencing data of East Asian populations in the NGS panel (supplementary fig. S22, Supplementary Material online) and estimated the TMRCA of this specific paternal lineage. We found that paternal lineage O1b1a1a (O-M95) was dominated by Bai-Yue populations (55/62), including HNL, CDX (Dai), and KHV (Kinh). We then estimated that this paternal lineage appeared at least in 10,998 ya (95% confidence interval [CI]: 10,082–12,651 ya; fig. 3*A* and supplementary table S10, Supplementary Material online). As for sublineages of O1b1a1a (O-M95), we found that the HNL individuals under O1b1a1a (O-M95) all belonged to the sublineage O1b1a1a1a1. We also found that individuals belonging to sublineage O1b1a1a1a1a were mainly Dai (12/32), Kinh (11/32), and HNL (6/32), whereas the sublineage O1b1a1a1a1b was dominated by HNL (13/23) and Dai (7/23). This may suggest a closer genetic relationship at a paternal level between HNL and Dai, and the divergence of HNL and Dai occurred later than that of HNL and Kinh. We also observed that there were two evident divergences between HNL and Dai under the O1b1a1a1a1b sublineage occurring 2,700 ya (95% CI: 2,025–3,437 ya) and 2,828 ya (95% CI: 2,151–3,280 ya).

To infer the fine-scale population history of HNL with Bai-Yue lineage, we applied a multiple sequentially Markovian coalescent (MSMC; Schiffels and Durbin 2014) to estimate the historical effective population size (*N_e_*) using Han and mainland Bai-Yue populations (Dai and Kinh) for comparison (fig. 3*B*). The results showed that *N_e_* of HNL.Admixed was consistently higher than that of HNL.Main since ∼20,000 ya (fig. 3*B*), probably resulting from the higher genetic similarity between HNL.Admixed and Han. We also observed that Bai-Yue populations, including HNL, Dai, and Kinh, all experienced a bottleneck ∼7,400 ya when the Han Chinese underwent population expansion in the early Neolithic Age. In addition, HNL.Main continued to experience bottlenecks since ∼4,000 ya, consistent with the timing of large-scale migration of the Li population to Hainan Island from South China, when all the other mainland populations experienced substantial increases of *N_e_*. We also estimated the *N_e_* based on genome-wide genealogies using *RELATE* (Speidel et al. 2019). Although *RELATE* yielded lower estimated values, the overall pattern was consistent with that of MSMC (supplementary fig. S23*A*, Supplementary Material online). Recent demography inferred from IBD segments using *IBDNe* (Browning and Browning 2015) also illustrated that HNL.Main showed an elevated decrease of *N_e_* compared with Han and mainland Bai-Yue populations (supplementary fig. S24, Supplementary Material online). We then estimated that HNL.Main divergence from Han occurred ∼13,000–7,900 ya, much earlier than the divergence between HNL.Admixed and Han ∼3,600 ya (supplementary fig. S23*B* and *C*, Supplementary Material online). In addition, we estimated the divergence time between HNL.Main and the mainland Bai-Yue populations such as Dai and Kinh began ∼3,600 ya (supplementary fig. S23*B*, Supplementary Material online). This divergence was followed by other two divergences between HNL and Dai within the O1b1a1a1a1b sublineage ∼2,800–2,700 ya, suggesting the time of population differentiation among the ancient Bai-Yue lineages.

### Local Adaptation

**Fig. 4.**
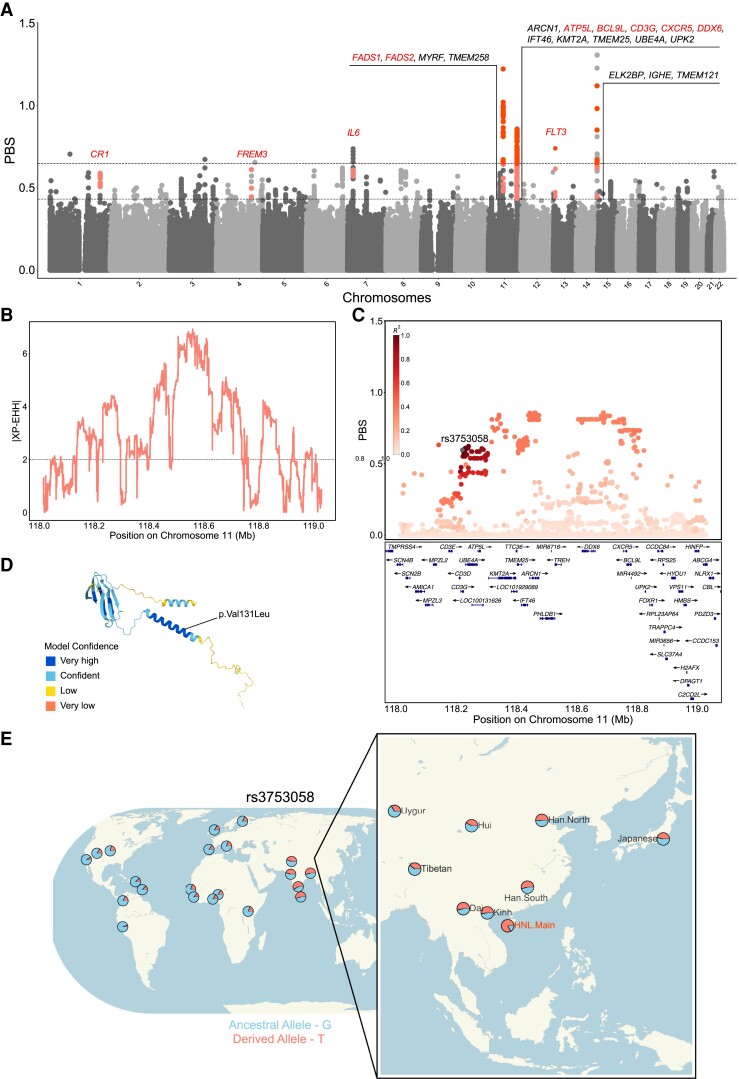
Local adaptation identified in HNL. (*A*) Manhattan plot showing the PBS values in genome-wide scan for HNL.Main, using the Han and CEU as reference populations. The 99.995th and 99.999th percentiles of the PBS distribution are shown as dashed horizontal lines. Gene symbols with PBS values over the 99.999th percentile and with PBS over the 99.995th percentile but showing putative functions are labeled. Gene symbols with putative functions are highlighted with color. Corresponding variants over the 99.999th and 99.995th percentiles are colored as dark and light dots, respectively. (*B*) Example of a genomic region located in chromosome 11 under positive selection identified by the XP-EHH method. (*C*) Local PBS distributions of example putative functional adaptive variant rs3753058 within *CD3G*. The concerning adaptive variant (rs3753058) is labeled, whereas other SNVs are colored according to pairwise linkage disequilibrium with this variant based on the HNL-Han-CEU trio data set. (*D*) The protein tertiary structure predicted by *AlphaFold* shows the functional consequences of the loss-of-function variant rs3753058 within *CD3G*. (*E*) Population prevalence for the DAF of the rs3753058 based on the *PGG.SNV* database.

To investigate the potential population-specific adaptation of HNL.Main, we applied population branch statistics (PBS; Yi et al. 2010) to perform a genome-wide scan using the Han and CEU as the ingroup and outgroup reference populations, respectively. Notably, in the HNL-Han-CEU trio, we pinpointed several evident haplotype blocks with strong PBS signals that suggest recent positive selections with specific functions in HNL (fig. 4*A* and supplementary fig. S25, Supplementary Material online). These selection signals determined by PBS were also supported by the iHS and/or XP-EHH approaches (supplementary fig. S26, Supplementary Material online).

The strongest PBS signal of selection was an ∼110 kb region encompassing four genes located on chromosome 11 comprising *FADS1*, *FADS2*, and their upstream genes *MYRF* and *TMEM258* (fig. 4*A* and supplementary fig. S27*A*, Supplementary Material online). The genes *FADS1* and *FADS2* encode the fatty acid desaturase (FADS) enzymes that involve the determinants of long-chain (LC-) polyunsaturated fatty acid (PUFA) levels in lipid metabolism. We found that rs174570 had the top selection signal within the FADS region (PBS = 1.22), which has been reported as an evident and potentially functional selection signal identified from Greenlandic Inuit (Fumagalli et al. 2015). In addition, a reported variant under selection in Indonesia Flores pygmy (Tucci et al. 2018), rs174547-C tagging ancestral haplotype, was also fixed in HNL with 0% derived allele frequency (DAF) as another evident selection signal (PBS = 1.02; supplementary fig. S27*B*, Supplementary Material online). In particular, the selected derived allele of rs174570-T and the ancestral allele of rs174547-C are both associated with the down-regulation of *FADS1*, lowering the ratio of conversion from the short-chain (SC-) to LC-PUFA (supplementary fig. S27*C*, Supplementary Material online).

Malaria was prevalent in Central and South Hainan and overlapped with the main settlement of HNL (Xiao et al. 2010, 2012). Thus, we also focused on the selection signals related to malaria pathogenesis. We identified variants of three genes for which the PBS values were in the top 0.005% percentile, *CR1*, *FREM3*, and *IL6* (fig. 4*A*), genes that have been reported to be associated with malarial susceptibility and/or severity. The *CR1* encodes a membrane glycoprotein found on different types of blood cells. It was reported as being a receptor for the invasion of red blood cells by the parasite (Stoute 2011). As for *FREM3*, it was identified as a selection or GWAS signal of malaria in African populations in previous studies (Malaria Genomic Epidemiology et al. 2015; Ndila et al. 2018; Ravenhall et al. 2018; Choudhury et al. 2020), and the polymorphism of *FREM3* was reported to be associated with differential susceptibility to severe malaria (Ndila et al. 2018; Choudhury et al. 2020). This association is probably because *FREM3* is close to a cluster of glycophorin genes (supplementary fig. S28*A*, Supplementary Material online; *GYPA*, *GYPB*, and *GYPE*) that encode blood group antigens for malaria resistance (Malaria Genomic Epidemiology et al. 2015; Ndila et al. 2018). The last gene with significant PBS signals was *IL6* which encodes interleukin-6 and is one of the indicators of malaria severity (Kern et al. 1989; Mbengue et al. 2016). Overall, these genes with strong selection signals suggest positive selection induced by malaria resistance in the HNL.

Since a high incidence of blood disorders is accompanied by a high prevalence of malaria infection, we were also concerned with genes that were involved in hematopoiesis or blood disorders. We first focused on the haplotype block with a strong selection signal located in a ∼610 kb region of chromosome 11 involving 11 genes with variants having high PBS values (fig. 4*A*). This region also showed strong selection signals by the XP-EHH method (fig. 4*B*). Among these genes, we found five genes, *ATP5L*, *BCL9L*, *CD3G*, *CXCR5*, and *DDX6*, that have been reported to be associated with the occurrence of B-cell lymphomas, a blood cancer caused by the disorder of immune functional B cells (also known as B lymphocytes) that attack invading pathogens. Notably, we found a missense variant rs3753058 within *CD3G* that showed a strong selection signal (PBS = 0.6; fig. 4*C*) and putative loss of function, since it was predicted to be damaged by Sorting Intolerant from Tolerant (SIFT) (Kumar et al. 2009), Polymorphism Phenotyping (PolyPhen) (Adzhubei et al. 2010), and Combined Annotation Dependent Depletion (CADD) (Rentzsch et al. 2019) methods. The protein encoded by *CD3G* is a part of the T-cell receptor (TCR)–CD3 complex that plays an essential role in the adaptive immune response. The derived allele (T) of *CD3G*-rs3753058 could change the position 131 of the *CD3G* protein sequence (Ensembl protein ID ENSP00000431445) from valine to leucine (p.Val131Leu; fig. 4*D*). This derived allele is enriched in East Asians (∼30–50%) and particularly shows a much higher frequency in HNL (82.29%) compared with other global populations (fig. 4*E*). Moreover, we also found another gene located on chromosome 13 with strong PBS signals, *FLT3* (fig. 4*A*), that is involved in the regulation of hematopoiesis and the development of lymphocytes. In addition, most of these genes with selection signals showed relatively high expression levels in tissues related to B cells such as spleen and Epstein-Barr virus (EBV)-transformed lymphocytes in the GTEx data set (supplementary fig. S29, Supplementary Material online). Collectively, we speculated that these genes under selection could be malaria driven and have become a part of the genetic contribution to immune-related blood traits in present-day HNL.

Finally, to investigate the interactions of genes putatively under selection, we performed functional enrichment using genes with variants for which the PBS value was over the top 0.005% percentile (supplementary table S11, Supplementary Material online). We found that a hematopoietic cell lineage (KEGG: hsa04640) was identified as having the strongest signal in the enrichment analysis (supplementary fig. S30 and table S12, Supplementary Material online). In addition, we also searched for adaptive signals of polygenic selection in HNL from the *KEGG* database (Kanehisa et al. 2017) by determining whether the PBS distribution of variants in a gene set was significantly shifted toward larger values than the rest of the genes across the genome. We detected 13 gene sets showing an overall significantly larger distribution of PBS values as candidates for polygenic selection (Bonferroni *P*-value <0.05; supplementary table S13, Supplementary Material online) and found that the hematopoietic cell lineage (KEGG: hsa04640) was also identified as a candidate pathway for polygenic selection. These results again confirmed that a local adaptation of hematopoietic function has occurred in HNL.

### Evolutionary Scenario of Bai-Yue Lineage

**Fig. 5.**
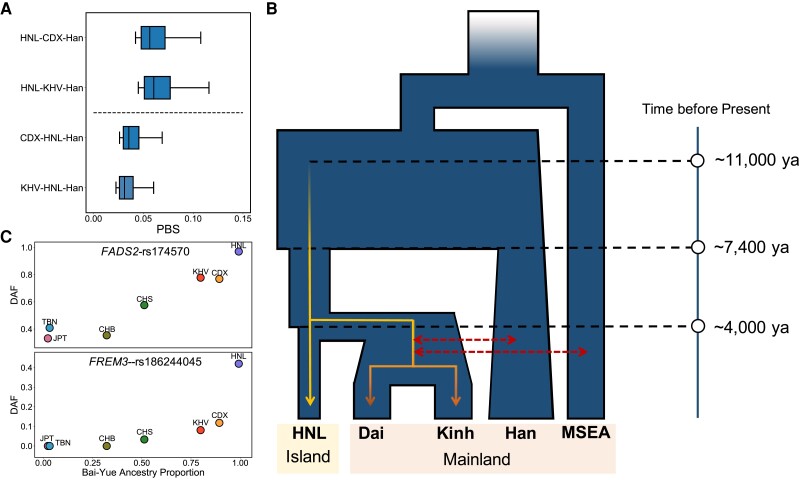
A proposed model of Bai-Yue lineage evolution. (*A*) Comparison of PBS distributions using HNL or mainland Bai-Yue as the target population with the remaining one as an ingroup reference population. Han was used as an outgroup reference population in each PBS calculation. Only PBS values higher than the 90th percentile and lower than the 99.995th percentile were used for comparison. (*B*) A simplified model based on the inferred population history of Bai-Yue populations, explaining the possible scenario of differentiated population histories and local adaptations within Bai-Yue lineage. (*C*) Example adaptive variants supporting the results of the proposed evolution model. CDX: Chinese Dai in Xishuangbanna, China; CHB: Han Chinese in Beijing, China; CHS: Han Chinese South; JPT: Japanese in Tokyo, Japan; KHV: Kinh in Ho Chi Minh City, Vietnam; TBN: Tibetan.

To characterize differentiated adaptation within Bai-Yue populations, we also used HNL-CDX-Han and HNL-KHV-Han trios to search for candidate selection signals within Bai-Yue lineage (supplementary fig. S31*A* and *B*, Supplementary Material online). We found that a certain number of strong PBS signals, including genes in the FADS region and genes related to malaria and B-cell lymphomas, overlapped with the HNL-Han-CEU trio (supplementary fig. S31*C*, *D*and table S14, Supplementary Material online). We thus hypothesized that the differentiation between the island and mainland Bai-Yue populations could have been driven by the admixture between mainland Bai-Yue and surrounding mainland populations such as the Han. We then changed the target population and ingroup reference populations of these two trios as CDX-HNL-Han and KHV-HNL-Han and compared the PBS distribution with the original HNL-CDX-Han and HNL-KHV-Han trios. We found that the PBS distribution using HNL as the target population was significantly lower than those of mainland Bai-Yue populations (fig. 5*A*), indicating that mainland Bai-Yue populations shared more potential adaptations with the Han.

Combined with the previously inferred population history, we assumed that the founder effect in HNL preserved the high proportion of Bai-Yue ancestry and the local adaptations in ancient Bai-Yue, which were subsequently diluted in mainland Bai-Yue populations due to the gene flow (fig. 5*B*). For example, the DAF of rs174570-T on the *FADS2* locus decreased with a decrease of Bai-Yue ancestry proportion (fig. 5*C*). This observation was also consistent with the East Asian haplotype patterns of the FADS region, that is, populations with more Bai-Yue ancestry tended to harbor more haplogroups closer to the ancestral FADS haplotype (supplementary fig. S32, Supplementary Material online). As another example, we observed that *FREM3*-rs186244045, a typical Bai-Yue-specific variant, showed the highest DAF in HNL (42.71%) and was followed by mainland Bai-Yue populations, CDX (11.82%), and KHV (8.08%), whereas this derived allele is rare (<5%) or absent in other worldwide populations (fig. 5*C* and supplementary fig. S28, Supplementary Material online). These results suggested that the continental region intensified genetic affinity among mainland populations, while such an effect was much weaker on island populations due to the more isolated circumstances.

## Discussion

The present-day populations of once or currently speaking Tai-Kadai languages are mainly EAS.South and MSEA. As revealed by the *ADMIXTURE* analysis, Bai-Yue ancestry was widespread in EAS.South and MSEA and was well preserved in Bai-Yue populations in South China and North Vietnam (fig. 1*D* and supplementary fig. S8, Supplementary Material online). In particular, our analyses confirmed that these populations from South China and North Vietnam showed close genetic affinity and have a common genetic origin, the Bai-Yue lineage. We also observed the Bai-Yue ancestry in HNL.Main was homogeneous with the highest proportion of Bai-Yue ancestry (fig. 1*D*), which is likely to be a consequence of the isolated circumstance of Hainan Island. The Bai-Yue lineage is believed to have originated from South China and corresponds to the present-day Tai-Kadai-speaking populations (Jin et al. 2001; Bin et al. 2021; Yang et al. 2022). However, our results showed the Austroasiatic-speaking Kinh population presented evident genetic characteristics of Bai-Yue lineage as similar to the other Tai-Kadai-speaking Bai-Yue populations on the mainland. A previous study also illustrated that Austroasiatic-speaking Kinh, Muong, and Tibeto-Burman-speaking Phula and Lolo in North Vietnam were genetically closer to the Tai-Kadai-speaking populations rather than to other populations from the same language family (Liu et al. 2020). In addition, consistent with this previous study, we also observed that Tai-Kadai-speaking Colao and Lachi populations in North Vietnam show distinctiveness with specific genetic components from other mainland Bai-Yue populations, probably due to the strong genetic drift (Liu et al. 2020). As genetic and linguistic classifications can diverge in a population, we proposed that although present-day Bai-Yue populations mainly speak Tai-Kadai languages, Bai-Yue lineages also included populations speaking different language families.

A previous study based on sporadic Y-SNP markers estimated that the settling of HNL on Hainan Island occurred ∼44,500–11,300 ya based on the paternal lineage O-M95 (Li, Li, Ou, et al. 2008), whereas another study based on maternal mtDNA lineages proposed that the peopling of Hainan Island occurred ∼27,000–7,000 ya (Peng et al. 2011). However, due to the low resolution of the data and the lack of comparisons, the timing estimated from previous studies may be ambiguous. Moreover, due to strong genetic drift, uniparental markers are inclined to estimate the formation time of specific paternal or maternal lineages of HNL ancestors, rather than the timing of the settlement on Hainan Island. Taking advantage of the high-resolution NGS data, we estimated the formation time of the specific NRY lineage O-M95 of the Bai-Yue population as ∼11,000 ya (fig. 3*A*), an estimate that refines the possible origin time of the ancient Bai-Yue lineage. In addition, we observed the Bai-Yue populations experienced a bottleneck from ∼7,400 to ∼4,000 ya based on our MSMC estimation (fig. 3*B*), probably induced by the Han Chinese expansion in the Neolithic Age (Wang et al. 2013; Zhang et al. 2017). This hypothesis is also supported by our observation that multiple EAS.South and MSEA were modeled as an admixture of ancestry sources from HNL.Main and ancient northern East Asian ancestry (Bianbian) in *qpAdm* analyses (fig. 2*B*). Intriguingly, we also found that Han Chinese and Tujia with a strong Han Chinese genetic assimilation showed relatively high *f*_3_ values in outgroup *f*_3_ analyses of HNL (supplementary figs. S2*C*and S6, Supplementary Material online), which may be induced by the consistently increasing *N_e_* and large genetic variation of Han Chinese population.

Traditional historical records indicate that the HNL migrated from mainland South China to Hainan Island ∼4,000–3,000 ya (Du et al. 1993; Attané and Gu 2014). Our observations based on aDNA analyses indicated that HNL.Main show closer genetic affinity with ancient individuals from Vietnam and Fujian, China ∼4,000 ya rather than ancient Guangxi individuals ∼1,500 ya compared with other mainland Bai-Yue populations, which suggests the migration of HNL was much earlier than 1,500 ya (supplementary fig. S21, Supplementary Material online). In our MSMC estimates, we found that since ∼4,000 ya, the HNL experienced a continual population bottleneck (fig. 3*B*), whereas other mainland Bai-Yue populations (Dai and Kinh) displayed population growth after the previous bottleneck induced by the Han Chinese expansion since ∼7,400 ya (fig. 3*B*). Our observation suggests that the further HNL bottleneck was probably caused by the large-scale migration of the ancient proto-HNL from mainland South China to Hainan Island. In addition, previous linguistic research proposed that the Hlai language used by HNL diverged as a separate branch from other languages within the Tai-Kadai language family ∼4,000–3,000 ya (Bauer 2002; Diller et al. 2004; Blench et al. 2005). Our MSMC analyses estimated that the divergence between HNL and mainland Bai-Yue populations started from ∼3,600 ya (supplementary fig. S23, Supplementary Material online), in agreement with the time of linguistic divergence. Moreover, both previous studies (He et al. 2020; Li et al. 2020) and our observations based on the *f*_3_ tests indicated that HNL was an isolated population with low gene flow compared with other mainland Bai-Yue populations. The *f*_4_ tests of our study also illustrated that, compared with the mainland Bai-Yue populations, HNL show closer genetic connections with ancient southern East Asian ancestry and Austronesian-related ancestry, which may also be preserved by the early migration to Hainan Island. Collectively, we propose that the ancient Bai-Yue population lived in mainland South China before ∼4,000 ya, and a part of the ancient Bai-Yue population, that is, the proto-HNL, started migrating from the mainland to Hainan Island and became the main settlers ∼4,000–3,000 ya. The isolated circumstance of Hainan Island well preserved the ancient Bai-Yue ancestry in the HNL and prevented admixture with other populations, thus restricting the increase of *N_e_* growth of the HNL. In turn, the mainland Bai-Yue populations were admixed in various degrees with ancestries from other surrounding groups on the mainland, and this contributed to the increase of *N_e_* since ∼4,000 ya. Such an effect thus further resulted in the differentiation of gene pools of island HNL and mainland Bai-Yue populations. For example, rs174570 within *FADS2* and rs186244045 within *FREM3* are population-specific adaptive variants for HNL, whereas these have lower DAF in mainland Bai-Yue populations (fig. 5*C*). The diluted adaptations could have resulted from the admixture between mainland Bai-Yue populations and other surrounding mainland populations that have much lower DAF values of these adaptive variants.

The enzymes encoded by the FADS genes are involved in the biosynthesis of omega-3 and omega-6 LC-PUFAs that are enriched in individuals subsisting on animal-based diets but absent for those subsisting on plant-based diets (Ameur et al. 2012; Ye et al. 2017). The decrease and increase of *FADS1* expression are likely to respectively represent adaptations to low and high conversion efficiency from SC- to LC-PUFA, corresponding to animal- and plant-based diets (Ye et al. 2017; Mathieson and Mathieson 2018). In our study, we identified strong positive selection signals on *FADS1* and *FADS2* in Tai-Kadai-speaking HNL. We observed that rs174570 and rs174547 showed inverse patterns in DAF, but the alleles with high frequency in HNL were both associated with down-regulation of *FADS1*, that is, reducing the efficiency of conversion from SC- to LC-PUFA (supplementary fig. S27, Supplementary Material online). We found that other mainland Bai-Yue populations, though lower than HNL, also showed relatively high frequencies of these adaptive variants (fig. 5*C*). Even though Tai-Kadai speakers are regarded as corresponding to the origin of rice farmers from the Yangtze River Basin in ancient South China (Li, Huang et al. 2007; Molina et al. 2011; Gutaker et al. 2020; Wang, Yeh, et al. 2021), our observations suggest that their adaptation was driven by traditional animal-based diets rather than plant-based diets. We propose that such adaptation in East Asia could be traced to ancestors in the more ancient periods such as pre-Neolithic hunter-gatherers (Matsumura et al. 2019; Yang et al. 2020) rather than the farmers with a prosperous rice culture. This hypothesis may be supported by a previous aDNA study illustrating that present-day Tai-Kadai speakers in South China comprise a higher proportion of ancestry sources from a Liangdao hunter-gatherer than other Chinese populations (Wang, Yeh, et al. 2021). Additionally, we observed that the haplotype frequency of the FADS region is differentiated between southern and northern East Asians (supplementary fig. S32, Supplementary Material online). The rs174570 within *FADS2* with the highest PBS value was also identified as a highly differentiated variant between northern and southern Han Chinese in our previous study (Xu et al. 2009). Such differentiation could have resulted from the differences in local historical diets between northern and southern populations in East Asia, and also the more frequent admixture between the Bai-Yue population and southern Han Chinese.

The HNL settlement area was once a region with a high incidence of malaria. In the genome-wide scan of PBS, we identified several signals of local adaptation related to malaria infection, including *CR1*, *FREM3*, and *IL6* (fig. 4*A*). These genes are highly correlated with hematopoietic functions, implying strong interaction with parasite invasion. For example, *CR1* plays a key role in the Knops blood group on erythrocytes; the *CR1* polymorphisms can result in the CR1 deficiency and help confer protection against severe malaria (Cockburn et al. 2004; Kwiatkowski 2005). Such variants of *CR1* were reported to be under selection in populations living in Sardinia (Kosoy et al. 2011) or prevalent in other malaria-endemic regions such as Papua New Guinea, India, and Kenya (Cockburn et al. 2004; Thathy et al. 2005; Rout et al. 2011). In addition, both functional enrichment and tests of polygenic selection detected the pathway of hematopoietic cell lineage (KEGG: hsa04640) as evidence that genes related to human hematopoietic function in HNL were differentiated, probably due to malaria pathogenesis. We then focused on genes under selection in HNL associated with the occurrence of B-cell lymphomas (fig. 4*A*), a blood disorder that occurs at a higher incidence in equatorial areas endemic to malaria (Molyneux et al. 2012; Robbiani et al. 2015; Nelson 2016). These genes, including *ATP5L*, *BCL9L*, *CD3G*, *CXCR5*, *DDX6*, and *FLT3*, all showed relatively high gene expression levels in tissues highly correlated with B cells such as spleen and EBV-transformed lymphocytes (supplementary fig. S29, Supplementary Material online). Moreover, a previous epidemiological study also described a higher incidence of B-cell lymphomas occurring on Hainan Island compared with other types of malignant lymphomas (The Nationwide Lymphoma Pathology Cooperative Group 1985). The main functions of B cells are producing antibodies to attack invading pathogens and to be involved in the immune response against pathogenic infections. The parasites that affect human health, such as malaria pathogens, could interact directly with and manipulate B-cell functions (Nothelfer et al. 2015). Therefore, we propose that malaria-driven selection influenced the hematopoietic function and B-cell immunoreaction in the HNL and further increased the incidence of hematological diseases such as B-cell lymphomas.

In this study, our efforts on genetic structure, population history, and natural selection of HNL improved the understanding of Bai-Yue groups and Tai-Kadai-speaking populations. However, some limitations are also shown in our study. First, the sampling of HNL from different locations on Hainan Island is unbalanced in our study. In addition, the Li population is also distributed outside Hainan Island with a small amount. These sampling biases for the Li individuals result in difficulties to investigate the detailed substructure within HNL.Main in this study. Second, “Bai-Yue” is a historical and ethnological definition, rather than a linguistic classification. We caution that our proposed evolutionary model of Bai-Yue lineage may be not suitable for Tai-Kadai speakers from other mainland Southeast Asian countries such as Thailand since they were deemed to be admixed with South Asian populations (Kutanan et al. 2021). Third, even though mainland Bai-Yue populations share a relatively high proportion of Bai-Yue ancestry and are less isolated than HNL.Main, differences in genomic diversity were also observed among mainland Bai-Yue populations. For example, we found that mainland Bai-Yue populations show differences in admixture (supplementary fig. S12, Supplementary Material online) and within-population IBD sharing (supplementary fig. S14, Supplementary Material online). These observations probably suggest different genetic histories occurred in these populations. Further studies are also needed to investigate the genetic connections and differences among mainland Bai-Yue populations. With the extension of genetic studies for populations in South China and Southeast Asia, it is anticipated that the complex history of Bai-Yue lineage as well as divergent evolution within Tai-Kadai speakers will be further refined.

## Materials and Methods

### Ethical Statement

All procedures performed in studies involving human participants were approved by the Ethics Committee of Hainan Medical University (HYLL-2011-001), and in accordance with the 1964 Helsinki declaration, its later amendments, or comparable ethical standards. Informed consent was obtained from all individual participants included in the study. The personal identifiers of all samples, if any existed, were stripped off before sequencing and analysis.

### Sample Collection, Whole-genome Sequencing, and single-nucleotide variant calling

Peripheral blood samples were collected from 55 Li individuals living in 7 counties of Hainan Province, China (supplementary fig. S1*A*, Supplementary Material online). To extend the representativeness of the Li population, we randomly selected Li individuals aged over 40 years old in the middle-aged and aged generations, with an average age of 69 years old. Based on the questionnaires and statements of participants, each individual was officially recognized as Li nationality, and was the offspring of a nonconsanguineous marriage of members of the same nationality within three generations. The name and language affiliation of the Li population in this study were referred to the National Ethnic Affairs Commission of the People’s Republic of China (https://www.neac.gov.cn). The map of China used in this study was obtained from http://bzdt.ch.mnr.gov.cn under the approval number GS(2020)4618.

Whole-genome sequencing (WGS) data with high coverage (30–50×) for 150 bp paired-end reads was carried out on an Illumina HiSeq X10 platform (Wuxi NextCODE, Shanghai, China). Reads of each sample were mapped to the human reference genome (GRCh37) using *BWA-MEM* v0.7.10 (Li and Durbin 2010). We executed duplicate mark and base quality recalibration using *GATK* v3.8 (McKenna et al. 2010). WGS data of 33 Tibetan (TBN) samples from Lu et al. (2016) and 131 Han Chinese samples from the *PGG.Han* (https://www.hanchinesegenomes.org) database (Gao et al. 2020) was also collected for comparison. We performed a joint variant calling of HNL with Tibetan and Han Chinese samples as well as samples from the Simon Genome Diversity Project data set (Mallick et al. 2016) through the *HaplotypeCaller* module of *GATK* based on the GVCF mode and implemented strict quality control through variant quality score recalibration. As a result, 38,605,313 bi-allelic single-nucleotide variants (SNVs) with high quality were retained for downstream analyses. Among these SNVs, we observed 13,605,313 SNVs for HNL samples, including 362,034 (2.66%) novel variants based on *dbSNP* database (https://www.ncbi.nlm.nih.gov/snp) v154 (Sherry et al. 2001). Most of these novel variants were rare, with 83.46% singletons, 12.63% doubletons, 2.61% tripletons, 0.74% other rare variants, and only 0.55% of the novel variants were common with MAF ≥ 0.05. We further annotated these novel variants using Ensembl *Variant Effect Predictor* v94 (McLaren et al. 2016) and observed that most of these variants were intron variants (52.71%) or intergenic variants (35.03%), and 0.2% of the novel variants were annotated as loss-of-function categories (supplementary table S1, Supplementary Material online).

### Public Data Collection and Data Compilation

To investigate the population structure of HNL in a broader context, we used Human Origin (HO) Affymetrix data set (Lazaridis et al. 2014) representing diverse global populations as a comparison. In addition, five Tai-Kadai-speaking populations (Dong, Gelao, Maonan, Mulam, and Zhuang) living in South China from Wang, Yeh, et al. (2021), five Tai-Kadai-speaking populations (Colao, Lachi, Nung, Tay, and Thai), and Kinh living in North Vietnam from Liu et al. (2020) were also collected to extend our analyses. We distinguished the Thai population in Vietnam (from Liu et al.) and Thailand (from the HO data set) as Thai_V and Thai_T, respectively. Since Southeast Asia is close to Hainan Island and there are fewer Southeast Asian populations in the HO data set, we also collected genotype data including 178 Southeast Asians (Vietnamese individuals had been excluded to avoid the ambiguity with populations from Vietnam in other data sets) as references (Morseburg et al. 2016). We combined our joint-calling data set and multiple genotype data as a Global Panel data set (supplementary table S2, Supplementary Material online), which resulted in 118,942 SNVs. This data set is mainly used for analyses of population structure and genetic affinity.

The Global Panel data set shows limitations in the accuracy and density of genome-wide markers. To address more comprehensive analytical purposes, we combined our joint-calling data set with the 1,000 Genomes Project phase 3 (KGP) data set (1000 Genomes Project Consortium 2015) as the NGS panel (supplementary table S2, Supplementary Material online) to solve problems at the genome-wide level, including local ancestry inference, estimation of effective population size, inferring population separation, scan of natural selection, as well as other analyses when needed.

To investigate the ancestry of HNL on a larger time scale, we collected aDNA samples of EAS.South and MSEA from Allen Ancient DNA Resource (AADR) v44.3, the curated data set (https://reich.hms.harvard.edu/allen-ancient-dna-resource-aadr-downloadable-genotypes-present-day-and-ancient-dna-data) of public aDNA samples. In addition, aDNA samples from Guangxi and Fujian of South China (Yang et al. 2020; Wang, Wang, et al. 2021) were also collected. We selected samples that share more variants with the Global Panel data set and have a lower missing rate, resulting in 21 ancient samples from public research (Lipson et al. 2018; Yang et al. 2020; Wang, Wang, et al. 2021; Wang, Yeh, et al. 2021) that were retained in the final ancient data set (supplementary table S9, Supplementary Material online). We then merged the ancient data set with the Global Panel data set and filtered out SNVs with a missing rate >0.05. As a result, a total of 31,654 SNVs were retained as an Ancient Panel for *ADMIXTURE* analysis.

### Population Structure and Genetic Affinity

All of the HNL samples were self-reported to be unrelated, although we identified a total of four individuals (two pairs of two individuals) within third-degree relatedness using *KING* v2.1.2 (Manichaikul et al. 2010; supplementary fig. S1*B*, Supplementary Material online). To avoid the bias caused by a close genetic relationship, we excluded related samples within third-degree relationships for subsequent population structure analyses.

To investigate the population structure of HNL, *PLINK* v1.9 (Purcell et al. 2007) was used to carry out LD-pruning by first filtering out SNVs with a missing rate <0.05 and then selecting SNVs in the 200-kb nonoverlapping windows. A series of PCA at the individual level were performed by further analyzing populations of concern on the PC plot based on the same data set using *SNPRelate* v1.16.0 (Zheng et al. 2012). All the ancient individuals in supplementary table S9, Supplementary Material online were projected onto the PCA determined for present-day East Asian and Southeast Asian populations using *smartsnp* v1.1.0 (Herrando-Perez et al. 2021).

Weir and Cockerham’s *F_ST_* (Weir and Cockerham 1984) was used to measure the overall genetic differentiation among populations using *SNPRelate* v1.16.0 (Zheng et al. 2012) which allows the correction for different sample sizes of populations. The matrix of the unbiased *F_ST_* was used to construct a phylogenetic tree representing the genetic relationships between the HNL and surrounding populations.

*ADMIXTURE* v1.3.0 (Alexander et al. 2009) was applied to perform global ancestry inference, by assuming the number of ancestries (K) from 2 to 12 for the Global Panel and from 2 to 10 for the Ancient Panel. The input data for *ADMIXTURE* analysis were prepared using the same process as for the PCA. To lessen the bias caused by different sample sizes, we set 40 as the maximum sample size for each population. The admixture proportion of ancestry in a population was presented as mean ± standard deviation.

To examine the relatedness between HNL and populations in East Asia and Southeast Asia, we also computed the outgroup *f*_3_ statistics (Reich et al. 2009) using the program *qp3pop* implemented in *ADMIXTOOLS* v7.0.2 (Patterson et al. 2012). The form of *f*_3_(HNL, X; Yoruba) was used in the calculation, where X represents different East Asian and Southeast Asian populations, and the output *Z* score was used to measure the genetic affinity between HNL and different populations.

### Population Admixture Analyses

To detect potential admixture events in HNL and mainland Bai-Yue populations, we first applied haplotype-based *ChromoPainter* v2 (Lawson et al. 2012) to get the haplotype painting for all recipients and the copying vectors for all individuals from East Asia and Southeast Asia. We sampled 10 paintings per haplotype for recipients in *ChromoPainter*. *GLOBETROTTER* (Hellenthal et al. 2014) was further employed to explore potential population admixture of target populations using other East Asian and Southeast Asian populations as donors. A population with an “uncertain” as a best-guess conclusion was deemed difficult to describe admixture events in *GLOBETROTTER* inferences.

We applied *qpAdm* implemented in *ADMIXTOOLS* v7.0.2 (Patterson et al. 2012) to perform *f*_4_ statistics-based admixture modeling. To model the composition of ancient ancestry in present-day HNL.Main, we selected five ancient individuals, including (1) Bianbian representing ancient northern East Asian ancestry, (2) Qihe representing ancient southern East Asian ancestry (or proto-Austronesian ancestry), (3) Longlin in Guangxi related to Hòabìnhian ancestry, (4) and (5) LadaKH01 ∼1,500 years and HuatuyanNL21 ∼500 years ago in Guangxi who were close to the present-day Tai-Kadai speakers. We performed three-, two-, and single-source mixture models using different combinations of these ancient ancestries for HNL.Main and other Bai-Yue populations to estimate ancestry coefficients of each model and determine the model with the largest *P*-value as the best-fitting one for each population (supplementary table S8, Supplementary Material online). After we observed HNL.Main showed the best representativeness of a Bai-Yue ancestry among Bai-Yue populations of our study, we further used (1) HNL.Main, (2) Bianbian, (3) Qihe, and (4) Longlin as ancestral sources to model ancestral components of present-day EAS.South and MSEA (supplementary table S8, Supplementary Material online). The best-fitting model for each target population was determined by a similar process as described above.

To identify the ancestral sources of HNL.Admixed individuals, we performed local ancestry inference using *RFMix* v2.0.3 (Maples et al. 2013) with a 0.5 cM random forest window size and assuming the expected admixture generation as 150. The estimated ancestry proportions of HNL.Admixed were presented as mean ± standard deviation. We phased the data of the NGS panel using *Beagle* v5.2 (Browning et al. 2018) and used the genetic map from HapMap (International HapMap Consortium 2007). The local ancestry inference was carried out using 48 unrelated HNL.Main individuals and randomly selected 50 Han individuals as ancestral populations based on phased VCF. The results of local ancestry of genomic regions were visualized by *karyoploteR* v1.16.0 (Gel and Serra 2017). We further estimated the admixture time of HNL.Main and Han. We used *MultiWaver* v2.0 (Ni et al. 2019) which supports automatically selecting the best-fitting admixture model based on the distribution of ancestral segments. We carried out *MultiWaver* analysis with the default parameters based on the output results of ancestral segment distribution generated by *RFMix*, and the hybrid isolation model was determined as the best-fitting model as *MultiWaver* described.

### Analyses of Uniparental Genomes

To construct a paternal and maternal genealogy of HNL, we classified NRY haplogroups using *Y-LineageTracker* v1.3.0 (Chen et al. 2021) based on the ISOGG Y-DNA tree v2019-2020 (https://isogg.org/tree), and mtDNA haplogroups using *HaploGrep* v2.1.16 (Weissensteiner et al. 2016) based on a PhyloTree mtDNA tree v17 (https://www.phylotree.org/tree; Van Oven 2015). To investigate the population structure at paternal and maternal levels more comprehensively, we also collected NRY and mtDNA haplogroup data of East Asian and Southeast Asian populations from published research (Kong et al. 2003; Wen et al. 2005; Hammer et al. 2006; Hill et al. 2007; Li, Cai, et al. 2007; Li, Zhong, et al. 2007; Jin et al. 2009; Li et al. 2010; Zhao et al. 2010; Delfin et al. 2012, 2014; Ko et al. 2014; Trejaut et al. 2014; 1000 Genomes Project Consortium 2015; Lu et al. 2016; Poznik et al. 2016; Song et al. 2019; Gao et al. 2020; He et al. 2020; Ma et al. 2021) for comparison (supplementary tables S4 and S5, Supplementary Material online) and performed PCA based on haplogroup frequency. We also calculated the haplogroup diversity for each population following the formula: $\mathrm{HD}=\frac{n\left(1-\Sigma x^{2}\right)}{n-1}$, where *n* is the sample size of each population and *x* is the frequency of each haplogroup in each population.

To investigate the specific paternal lineages of Bai-Yue populations on a fine scale, we used Y-chromosomal sequencing data of HNL, TBN, and East Asians of the KGP data set in the NGS panel comprising 290 male samples with sufficient coverage and covering the main NRY haplogroups in East Asia. To construct an NRY phylogeny and estimate the coalescent times of haplogroups, we applied *BEAST* v2.6.0 (Bouckaert et al. 2014) to perform Bayesian evolutionary analyses using the GTR model under the strict clock and mutation rate of 7.6 × 10^−10^. The age of NRY haplogroup CT-M168 (71,760 years, 95% CI = 69,777–73,799) was used for calibration in age estimation (Karmin et al. 2015). The final consensus tree was constructed by the *TreeAnnotator* module implemented in *BEAST* and visualized by *FigTree* v1.4.4 (http://tree.bio.ed.ac.uk/software/figtree).

### Runs of Homozygosity, *f* Statistics, and Identity by Descents

We identified ROH in the HNL and other East Asian populations under the NGS panel using *BCFTOOLS* v.1.6 (Narasimhan et al. 2016) based on the Hidden Markov Model approach. We used the *-G* option and set the argument as 30 to account for GT errors. We classified ROH with lengths of ≤0.5, 0.5–1, and >1 Mb as short, medium, and long ROH, respectively. We calculated the number of ROH for each individual in each classified ROH category and calculated the average length of ROH as $\mathrm{the}\;\mathrm{average}\;\mathrm{length}\;\mathrm{of}\;\mathrm{ROH}=\frac{\mathrm{the}\;\mathrm{total}\;\mathrm{length}\;\mathrm{of}\;\mathrm{ROH}}{\mathrm{the}\;\mathrm{number}\;\mathrm{of}\;\mathrm{ROH}}$.

To test the potential admixture of HNL, we calculated *f*_3_ statistics in the form of *f*_3_(X, Y; HNL) using *qp3pop* implemented in *ADMIXTOOLS* v7.0.2 (Patterson et al. 2012), where X and Y represented all the possible population combinations of East Asian and Southeast Asian populations. We also calculated *f*_3_ statistics in the form of *f*_3_(HNL.Main, Han; mainland Bai-Yue) and *f*_3_(mainland Bai-Yue, Han; HNL.Main) to compare the admixture between HNL.Main and other mainland Bai-Yue populations. We used *qpDstat* in *ADMIXTOOLS* to calculate *f*_4_ statistics in the form of *f*_4_(HNL.Main, mainland Bai-Yue groups; Bianbian/Qihe, Yoruba) to investigate the genetic relationships with ancient northern and southern ancestries (Yang et al. 2020). We also performed *f*_4_(mainland Bai-Yue groups, X; HNL.Main, Yoruba) to further investigate the genetic characterization of isolated HNL compared with other Bai-Yue populations. To measure and compare the genetic connections between ancient individuals and present-day populations, we first merged the Global Panel data set of present-day populations with every single ancient individual in the Ancient Panel data set to create multiple specific data sets for *f*_3_ calculations (supplementary table S9, Supplementary Material online). We then used ancient individuals and present-day populations of EAS.South and MSEA as X and Y to calculate outgroup *f*_3_ statistics in the form of *f*_3_(X, Y; Yoruba).

We applied *hap-IBD* (Zhou et al. 2020) to estimate the IBD sharing segments within and between populations. The genotype data were phased using *Beagle* v5.2 (Browning et al. 2018) to estimate the IBD blocks among individuals. Both IBD and HBD blocks identified by *hap-IBD* were used as IBD sharing segments in our analyses. The total length of IBD sharing segments in each pair of individuals was used to evaluate the shared IBD between two individuals. We also calculated the average total length and number of IBD between HNL.Main and populations of EAS.South and MSEA. We further inferred a recent change in effective population size (*N_e_*) within 60 generations of Han and Bai-Yue populations using *IBDNe* v23Apr20 (Browning and Browning 2015). We set the minimum length of IBD segments to be used in each *IBDNe* estimation within the population as 2 cM.

### Detection of Archaic Introgression

We applied *ArchaicSeeker* v2.0 (Yuan et al. 2021) to detect archaic introgression in the present-day populations, using Denisovan (Meyer et al. 2012) and Altai Neanderthal (Prufer et al. 2014) as archaic genomes in the analysis. To test the correlation between archaic ancestry and Bai-Yue ancestry enriched in HNL, we first performed global ancestry inference of HNL and five other mainland East Asian populations (CDX, CHB, CHS, KHV, and TBN). We used the result of *K* = 2 with the lowest CV-error in *ADMIXTURE* to profile Bai-Yue ancestry proportions (supplementary fig. S17, Supplementary Material online). We then calculated the archaic ancestry proportion of these East Asian populations to test the correlation between archaic ancestry proportion and Bai-Yue ancestry proportion. Based on the results of *ArchaicSeeker*, we also searched HNL-specific archaic introgression segments that were enriched in HNL.Main but showed relatively lower frequency in other global populations.

### Inference of Population Demography

We applied *MSMC* v2.1.2 (Schiffels and Durbin 2014) to estimate the long-term effective population size of HNL, Dai, Kinh, and Han from high-coverage genomes in the NGS panel. The mask files and single-sample VCF files were generated from BAM files and phased data of the NGS panel, respectively. The estimates of *N_e_* were based on autosomal sequences by analyzing four genomes (eight haplotypes) for each population separately. Population separation between each pair of populations was estimated using four autosomal sequences from two individuals of each population. We assumed a mid-point of 0.5 as the start of separation and a point of 0.2 as when the two populations were separated. We used 64 segments for each MSMC estimation and scaled the output parameters to real-time and population sizes using an autosomal neutral mutation rate of 1.25 × 10^−8^ per base pair per generation and 25 years per generation.

We also employed *RELATE* v1.1.7 (Speidel et al. 2019) to estimate historical *N_e_* from the same samples as used for the MSMC. The hap/sample files were converted from phased VCF and were further processed as input files by the *PrepareInputFiles* module implemented in *RELATE*. We used the same genomic mask as that of MSMC and the human ancestor sequence of GRCh37 downloaded from Ensembl Release 71 (http://ftp.ensembl.org/pub/release-71/fasta/ancestral_alleles) in the process of preparing input files. The anc/mut files used for *N_e_* estimation were generated from hap/sample files by the *RelateParallel* module using a mutation rate of 1.25 × 10^−8^ per base pair per generation, default *N_e_* of haplotypes, and the genetic map from HapMap (International HapMap Consortium 2007). Finally, *N_e_* estimation was performed by the *EstimatePopulationSize* module with parameters of the mutation rate of 1.25 × 10^−8^ per base pair per generation and 25 years per generation.

### Scanning for Natural Selection

We applied PBS (Yi et al. 2010) to detect signals of recent positive selection at the genome-wide level. We used variant sites with a depth above 10× and a missing rate of <5% for PBS calculation. The PBS is defined as: $\mathrm{PB}S_{A}=\frac{T_{\mathrm{AB}}+T_{\mathrm{AC}}-T_{\mathrm{BC}}}{2}$, where *T* = −log(1 − *F*_*ST*_); A is the target population for the selection scan, and B and C are ingroup and outgroup populations used as references, respectively. We only considered variant sites that were polymorphic in at least one of the three populations in the PBS calculation.

To detect specific selection signals in 48 unrelated HNL.Main individuals, we used Han and CEU as ingroup and outgroup populations, respectively. We focused on signals for which the PBS values were above the 99.995th percentile. We also zoomed in on the local PBS distribution of selection signals concerning genes within 20-kb upstream and downstream and analyzed the LD pattern of the variant with the highest PBS value within the gene. To validate identified variants or genomic regions within concerning genes, we also estimated integrated haplotype scores (iHSs) and cross-population extended haplotype homozygosity (XP-EHH) of these genes within 20 kb upstream and downstream. The iHS and XP-EHH were both estimated using *selscan* v1.2.0 (Szpiech and Hernandez 2014), and the Han was used as a reference population in XP-EHH estimation.

We also explored differential selection within Bai-Yue lineage by comparing island (HNL) and mainland (CDX and KHV) Bai-Yue populations. We used HNL as a target population, assuming each of the other two mainland Bai-Yue populations as the ingroup population and HAN as the outgroup population.

### Functional Annotation of Natural Selection Signatures

To explore the detailed information of concerning variants with strong selection signals, we obtained the functional annotation and the global population prevalence from the *PGG.SNV* database (https://www.pggsnv.org; Zhang et al. 2019) and the association with gene expression from the GTEx Portal (https://gtexportal.org; GTEx Consortium 2013). The protein tertiary structure showing the functional consequence of the concerning variant was obtained from the *AlphaFold Protein Structure* database (https://alphafold.ebi.ac.uk; Jumper et al. 2021). We also referred to the reported cases from the *GWAS Catalog* database (https://www.ebi.ac.uk/gwas; MacArthur et al. 2017) to search previous published genome-wide associations concerning genes and variants.

To investigate the interactions of genes with strong PBS signals, we performed functional enrichment by *metascape* (https://metascape.org; Zhou et al. 2019), an online program that incorporates popular ontologies of functional categories. We used genes with variants of PBS values in the top 0.005% percentile as the input gene set. The top 20 functional categories with −log_10_(*P*-value) ≥ 2 were displayed as enriched terms. Similar functional categories were classified into one group, and the category with the summarized −log_10_(*P*-value) was shown in the enrichment figure.

To detect enrichment of PBS values in gene sets corresponding to a given biological pathway, we downloaded KEGG gene sets (Kanehisa et al. 2017) of *Homo sapiens* from the NCBI BioSystems database (http://www.ncbi.nlm.nih.gov/biosystems). We excluded nonautosomal genes and genes unmapped to the human reference genome of GRCh37 for each gene set and further excluded gene sets of less than ten genes. As a result, a total of 365 gene sets remained for the detection of polygenic selection. We compared the distributions of PBS in each gene set relative to the rest of the genes across the genome using one-sided Mann–Whitney *U* tests. Each gene set was tested independently and accounted for multiple testing using the Bonferroni correction.

## Supplementary Material

msac210_Supplementary_Data

## Data Availability

The genome data of 55 Hainan Li samples generated during this study are available in the National Omics Data Encyclopedia (NODE) at https://www.biosino.org/node and can be accessed with accession number OEP003168. Data application is conditioned on the following commitments: (1) the data will not be used for commercial purposes; (2) the data will not be shared with anyone else; and (3) no attempt will be made to identify any of the sample donors. Requests for access to data may be directed to xushua@fudan.edu.cn or heyungang@fudan.edu.cn.
