# Supplementary material for: Tracing Bai-Yue Ancestry in Aboriginal Li People on Hainan Island
Source: Mol Biol Evol. 2022 Sep 29;39(10):msac210. doi: 10.1093/molbev/msac210 (PMC9585476; doi:10.1093/molbev/msac210)
Supplement: msac210_Supplementary_Data [file msac210_supplementary_data.zip › MBE-22-0227.A.Supplement.pdf]

Tracing Bai-Yue ancestry in aboriginal Li people in Hainan Island

Supplementary Materials

Hao Chen,[1,10] Rong Lin,[2,6,10] Yan Lu,[3] Rui Zhang,[1] Yang Gao,[4] Yungang He,[5]* Shuhua Xu[3,4,7,8,9]*

[1]Key Laboratory of Computational Biology, Shanghai Institute of Nutrition and Health, University of Chinese Academy of Sciences, Chinese Academy of Sciences, Shanghai 200031, China;
[2]Department of Biology, Hainan Medical University, Haikou 571199, Hainan, China;
[3]State Key Laboratory of Genetic Engineering, Center for Evolutionary Biology, Collaborative Innovation Center for Genetics and Development, School of Life Sciences, Fudan University, Shanghai 200438, China;
[4]Human Phenome Institute, Zhangjiang Fudan International Innovation Center, and Ministry of Education Key Laboratory of Contemporary Anthropology, Fudan University, Shanghai 201203, China;
[5]Shanghai Fifth People's Hospital, and Shanghai Key Laboratory of Medical Epigenetics, International Co-laboratory of Medical Epigenetics and Metabolism (Ministry of Science and Technology), Institutes of Biomedical Sciences, Fudan University, Shanghai 200032, China;
[6]Center of Forensic Medicine of Hainan Medical University, Hainan Provincial Academician Workstation (tropical forensic medicine), Hainan Provincial Tropical Forensic Engineering Research Center, Haikou 571199, Hainan, China;
[7]Department of Liver Surgery and Transplantation Liver Cancer Institute, Zhongshan Hospital, Fudan University, Shanghai 200032, China;
[8]Center for Excellence in Animal Evolution and Genetics, Chinese Academy of Sciences, Kunming 650223, China;
[9]Jiangsu Key Laboratory of Phylogenomics and Comparative Genomics, School of Life Sciences, Jiangsu Normal University, Xuzhou, 221116, China;
[10]These authors contributed equally to this work.
*Correspondence: xushua@fudan.edu.cn (S.X.) or heyungang@fudan.edu.cn (Y.H.)

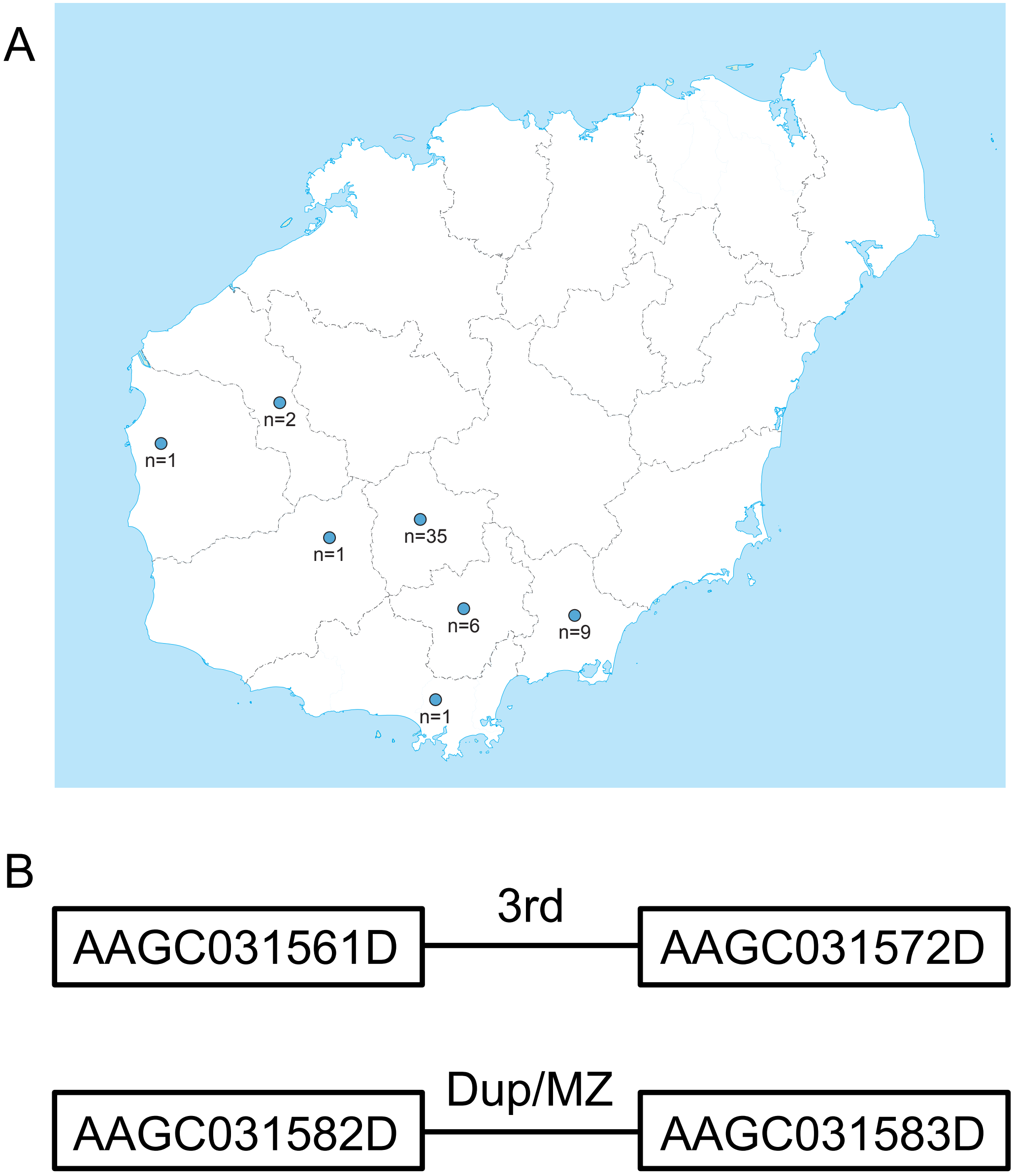

Fig. S1. Sample information of HNL.

(A) Sampling location of the Li population on Hainan Island (HNL). HNL samples were collected from 7 counties in Hainan Province, China. Each dot represents the sampling location and corresponding sample size. The map used in this figure was obtained from Hainan Administration of Surveying Mapping and Geoinformation (http://hism.mnr.gov.cn). (B) Relatedness within third-degree kinship among HNL samples identified by *KING* (Manichaikul, et al. 2010). 3rd: third-degree kinship; Dup/MZ: duplicates or monozygotic twins.

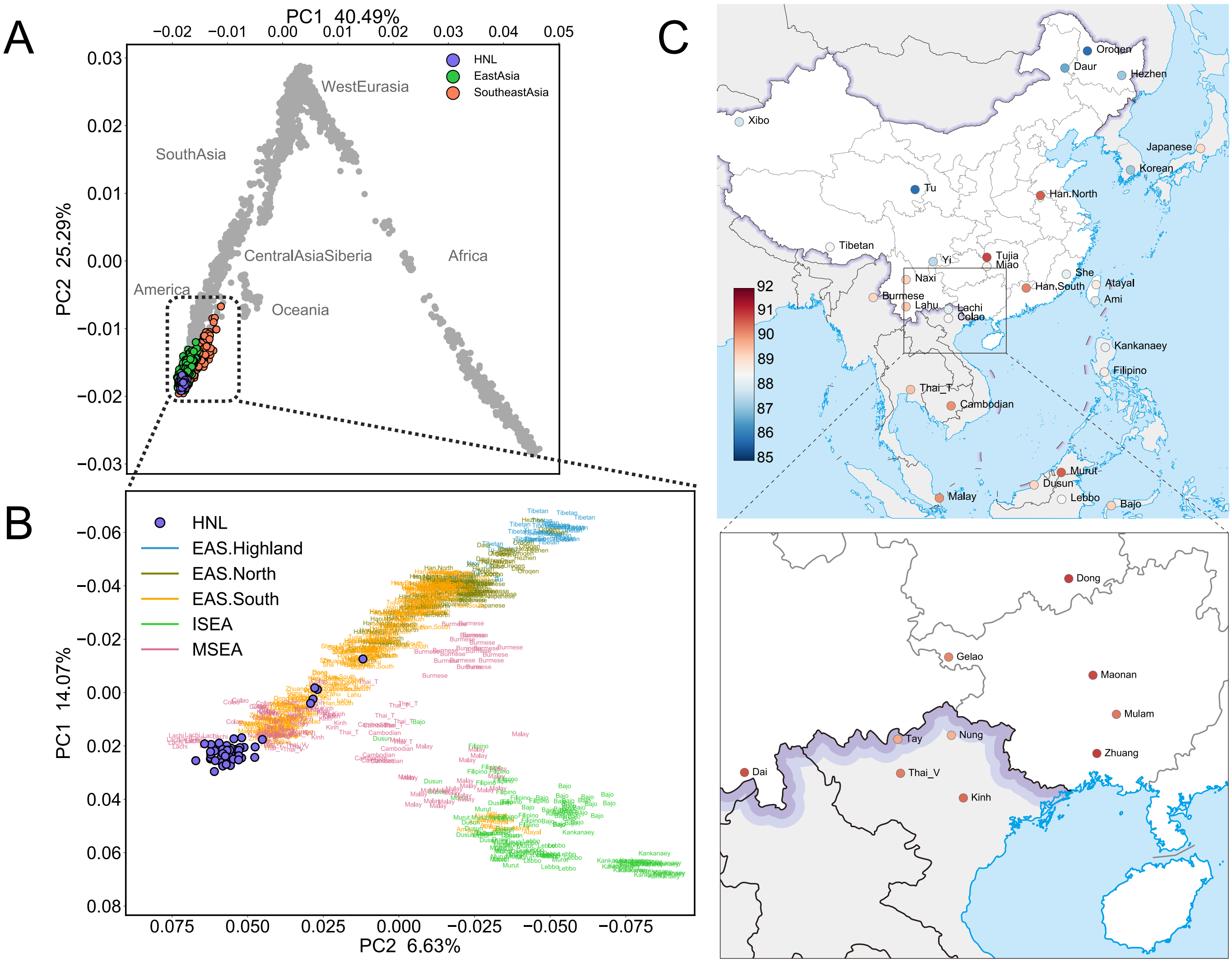

Fig. S2. Genetic affinity of HNL in the context of East Asia and Southeast Asia.

(A–B) PCA with 45,150 SNVs of (A) global populations (2896 individuals), and (B) East Asian and Southeast Asian populations (784 individuals). Population names were labeled in (B). (C) Outgroup f_3 in the form of f_3(X, HNL; Yoruba), assuming X is different East Asian and Southeast Asian populations. The map used in this figure was obtained from http://bzdt.ch.mnr.gov.cn (GS(2020)4618). EAS.Highland: East Asian highlanders; EAS.North: northern East Asians; EAS.South: southern East Asians; ISEA: island Southeast Asians; MSEA: mainland Southeast Asians.

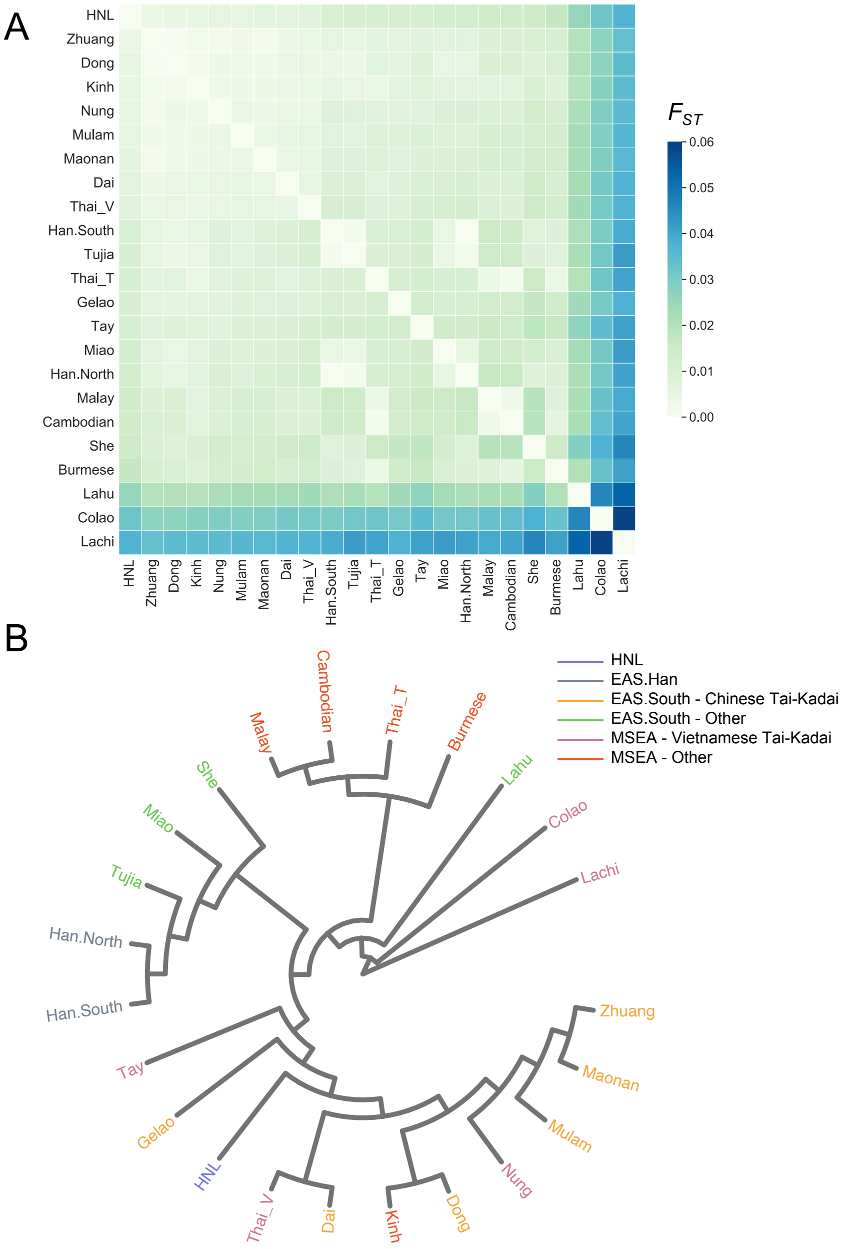

Fig. S3. Population differentiation in the context of southern East Asia and mainland Southeast Asia.

(A) Population differentiations measured by pairwise F_{ST} among southern East Asian (except Ami and Atayal) and mainland Southeast Asian populations. (B) Population phylogeny based on the result of pairwise F_{ST} in southern East Asian (except Ami and Atayal) and mainland Southeast Asian populations. EAS.Han: East Asian Han; EAS.South: Southern East Asians; MSEA: mainland Southeast Asians.

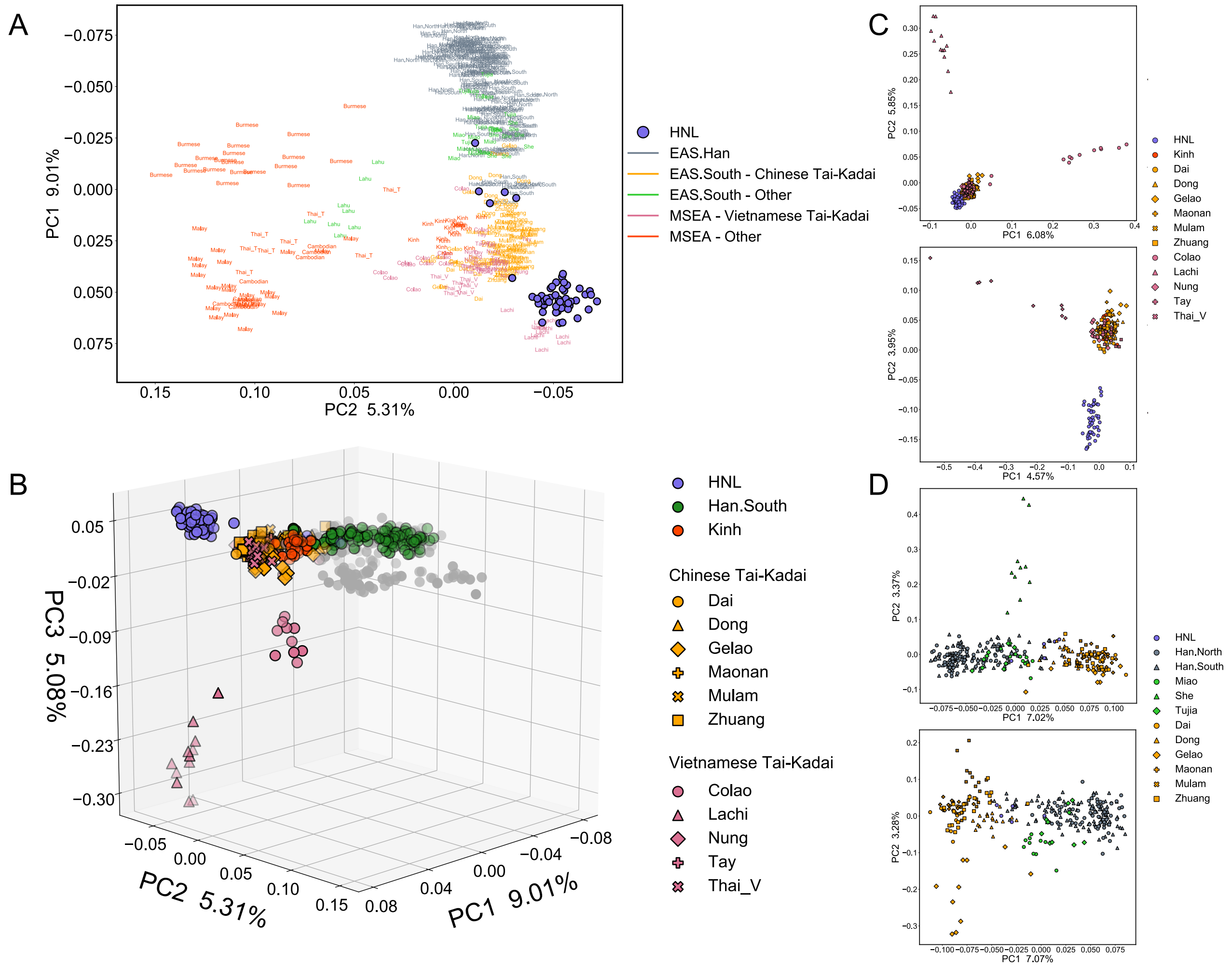

Fig. S4. PCA in the context of southern East Asia and mainland Southeast Asia.

(A) PCA performed by a total number of 45,150 SNVs in 502 individuals of southern East Asian and mainland Southeast Asian populations. (B) Three-dimensional PCA of 502 individuals from southern East Asia and mainland Southeast Asia. (C) PCA of the major cluster of HNL (HNL.Main), mainland Tai-Kadai-speaking populations, and Kinh. Populations close to HNL in PC1 and PC2 are colored and labeled on the PC plot. (D) PCA of the minor cluster of HNL (HNL.Admixed) and populations from southern East Asia (except Ami and Atayal). EAS.Han: East Asian Han; EAS.South: Southern East Asians; MSEA: mainland Southeast Asians.

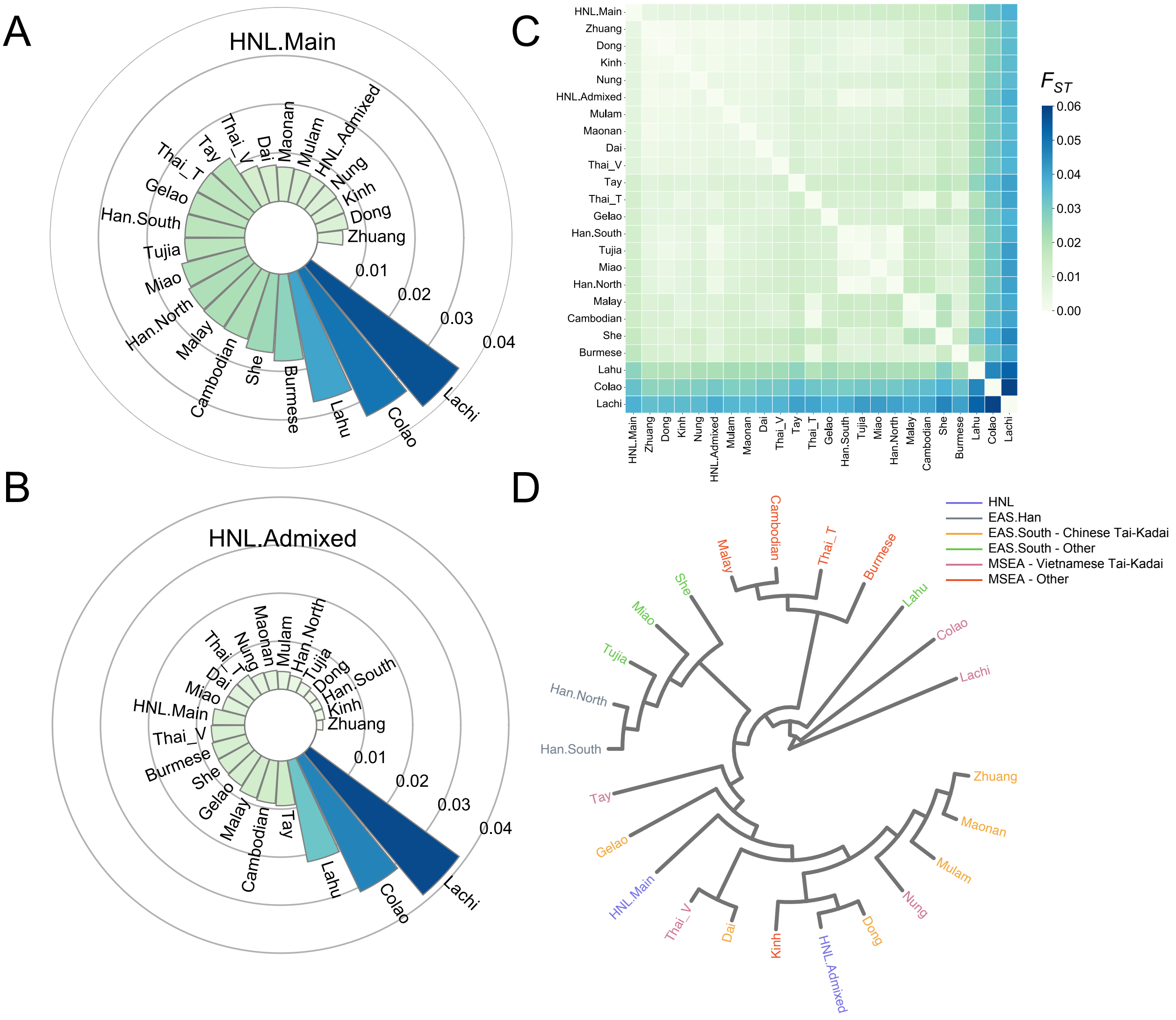

Fig. S5. Population differentiation of HNL subgroups in the context of southern East Asia and mainland Southeast Asia.

(A–B) Genetic affinity measured by F_{ST} between (A) HNL.Main / (B) HNL.Admixed and populations from southern East Asia (except Ami and Atayal) and mainland Southeast Asia. (C) Population differentiations measured by pairwise F_{ST} among southern East Asian (except Ami and Atayal) and mainland Southeast Asian populations. (D) Population phylogeny based on the result of pairwise F_{ST} in southern East Asian (except Ami and Atayal) and mainland Southeast Asian populations. EAS.Han: East Asian Han; EAS.South: Southern East Asians; MSEA: mainland Southeast Asians.

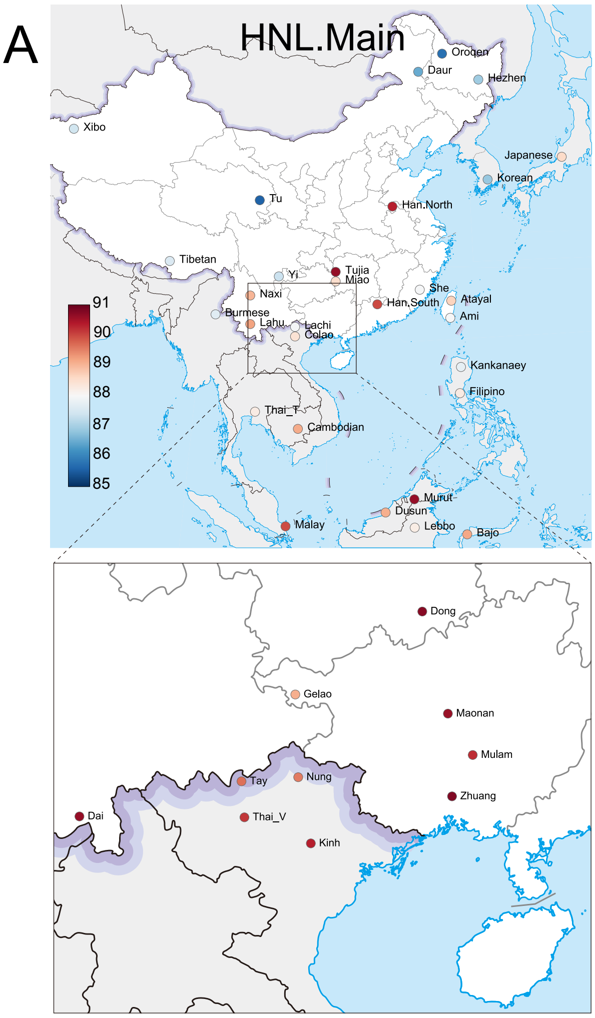

Fig. S6. Outgroup f_3 statistics of HNL subgroups compared with East Asians and Southeast Asians.

Outgroup f_3 statistics in the form of f_3(X, Y; Yoruba), assuming X is different East Asian and Southeast Asian populations, Y is (A) HNL.Main and (B) HNL.Admixed. The map used in this figure was obtained from http://bzdt.ch.mnr.gov.cn (GS(2020)4618).

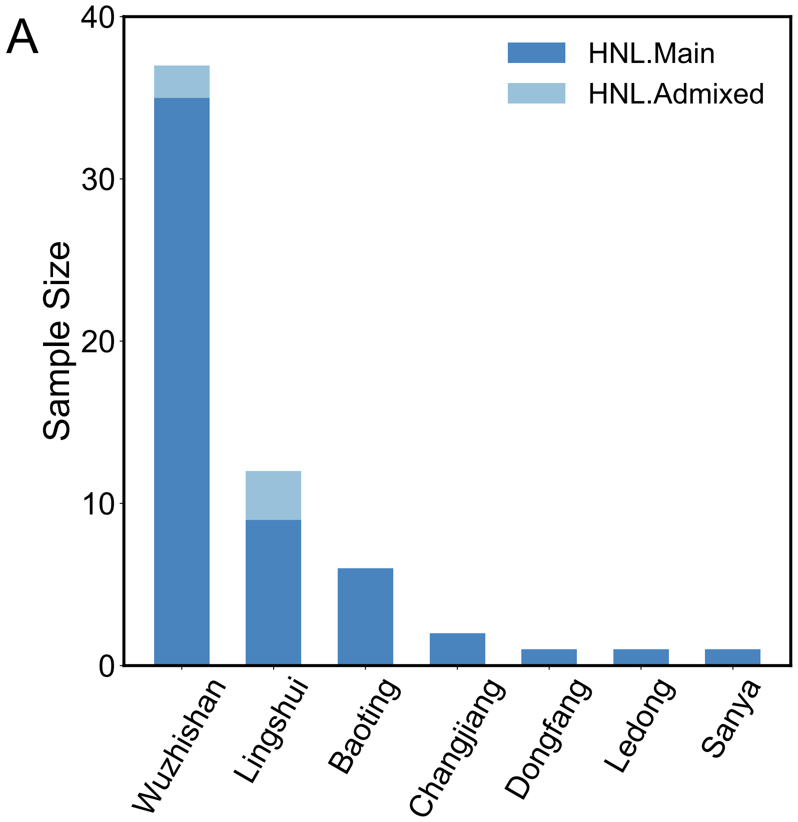

B

	Wuzhishan	Lingshui
HNL.Main	35	9
HNL.Admixed	2	3

Fig. S7. Sampling information of HNL subgroups.

(A) Sampling information of two HNL subgroups (HNL.Main and HNL.Admixed), x-axis represents sampling location and y-axis represents the sample size. (B) Contingency tables used for Fisher's exact test, using sampling information containing HNL.Admixed samples. The *P*-value of Fisher's exact test is 0.087, which indicated that HNL substructure is not correlated with sampling locations.

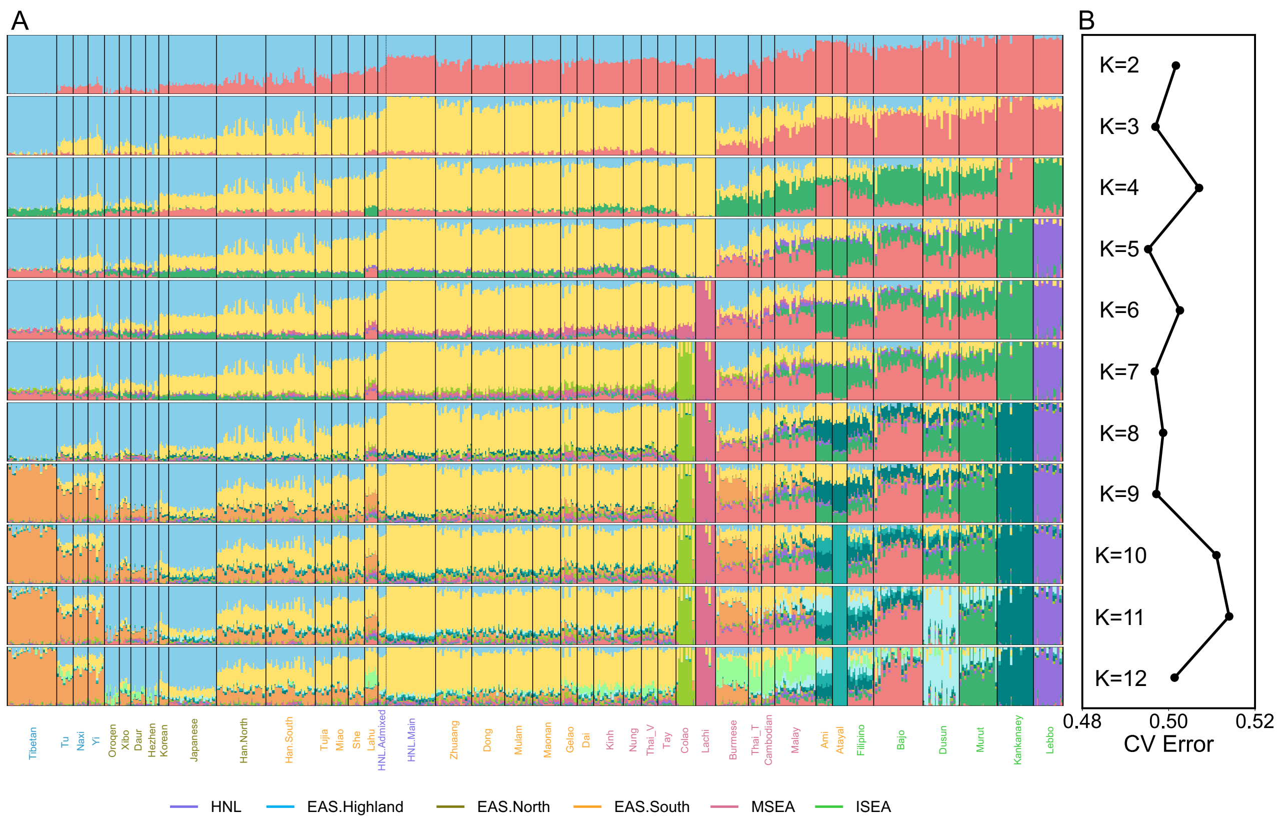

Fig. S8. Unsupervised *ADMIXTURE* of present-day East Asian and Southeast Asian populations.

(A) Unsupervised *ADMIXTURE* analysis (Alexander, et al. 2009) with the default parameters from K = 2 to K= 12 performed by 73,812 SNVs in 670 individuals of East Asian and Southeast Asian populations. The maximum sample size was restricted to 40 for populations with larger sample sizes. (B) CV error of current *ADMIXTURE* analysis from K = 2 to K= 12. EAS.Highland: East Asian highlanders; EAS.North: northern East Asians; EAS.South: southern East Asians; ISEA: island Southeast Asians; MSEA: mainland Southeast Asians.

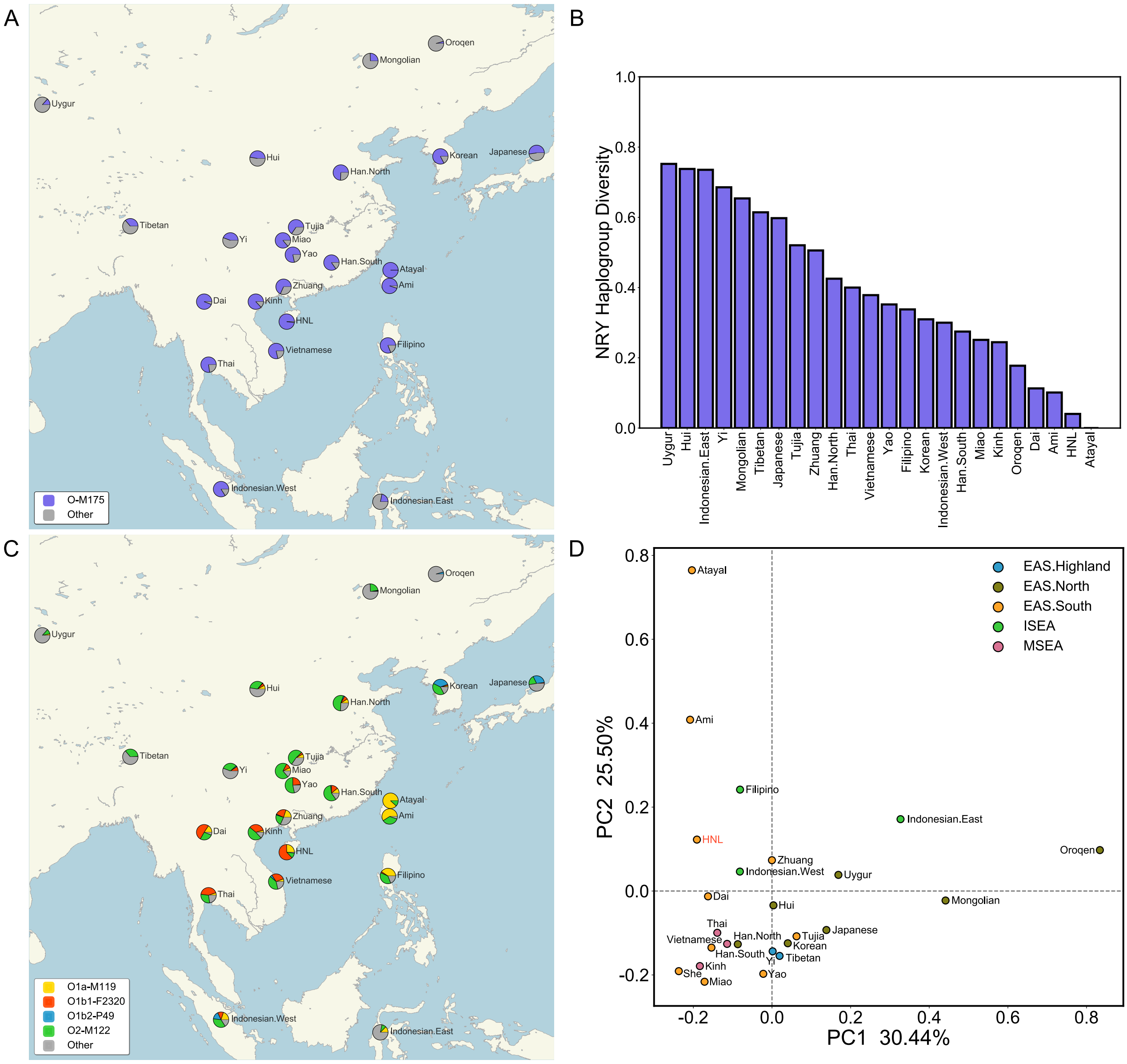

Fig. S9. Pattern of HNL-dominated NRY haplogroups in East Asian and Southeast Asian populations.

(A) Frequency of NRY haplogroup O-M175 in East Asian and Southeast Asian populations (Hammer, et al. 2006; Li, et al. 2010; Zhao, et al. 2010; Delfin, et al. 2012; Trejaut, et al. 2014; Lu, et al. 2016; Poznik, et al. 2016; Song, et al. 2019; Gao, et al. 2020; He, et al. 2020; Ma, et al. 2021). (B) NRY haplogroup diversity of East Asian and Southeast Asian populations. (C) Frequency of O sub-haplogroups in East Asian and Southeast Asian populations. (D) PCA based on NRY haplogroup frequency of East Asian and Southeast Asian populations. EAS.Highland: East Asian highlanders; EAS.North: northern East Asians; EAS.South: Southern East Asians; ISEA: island Southeast Asians; MSEA: mainland Southeast Asians.

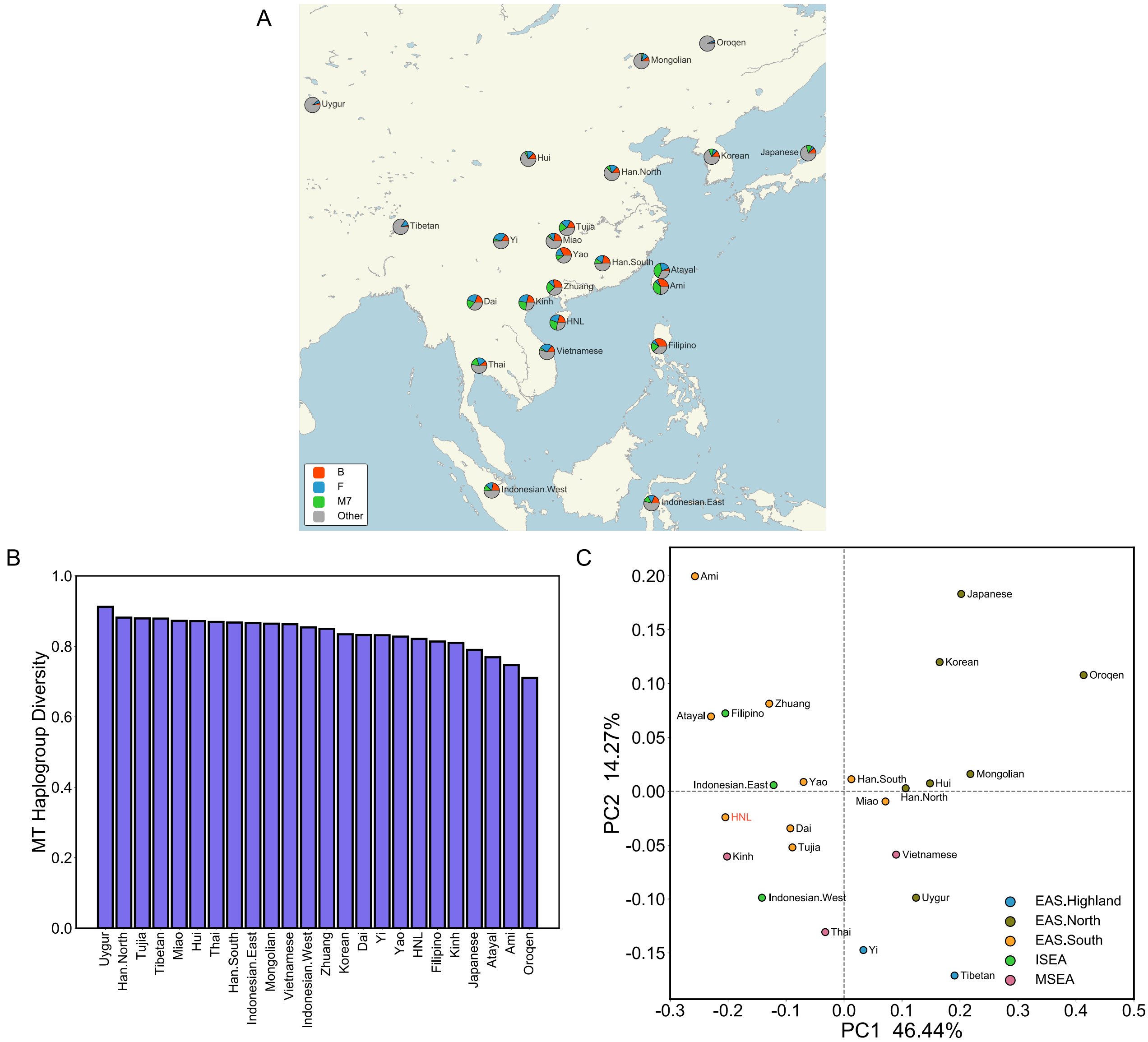

Fig. S10. Pattern of HNL-dominated mtDNA haplogroups in East Asian and Southeast Asian populations.

(A) Frequency of three HNL-dominated mtDNA haplogroups in East Asian and Southeast Asian populations (Kong, et al. 2003; Wen, et al. 2005; Hill, et al. 2007; Li, Zhong, et al. 2007; Li, Cai, et al. 2007; Jin, et al. 2009; Delfin, et al. 2014; Ko, et al. 2014; 1000 Genomes Project Consortium 2015; Lu, et al. 2016; Gao, et al. 2020; He, et al. 2020; Ma, et al. 2021). (B) MtDNA haplogroup diversity of East Asian and Southeast Asian populations. (C) PCA based on mtDNA haplogroup frequency of East Asian and Southeast Asian populations. EAS.Highland: East Asian highlanders; EAS.North: northern East Asians; EAS.South: southern East Asians; ISEA: island Southeast Asians; MSEA: mainland Southeast Asians.

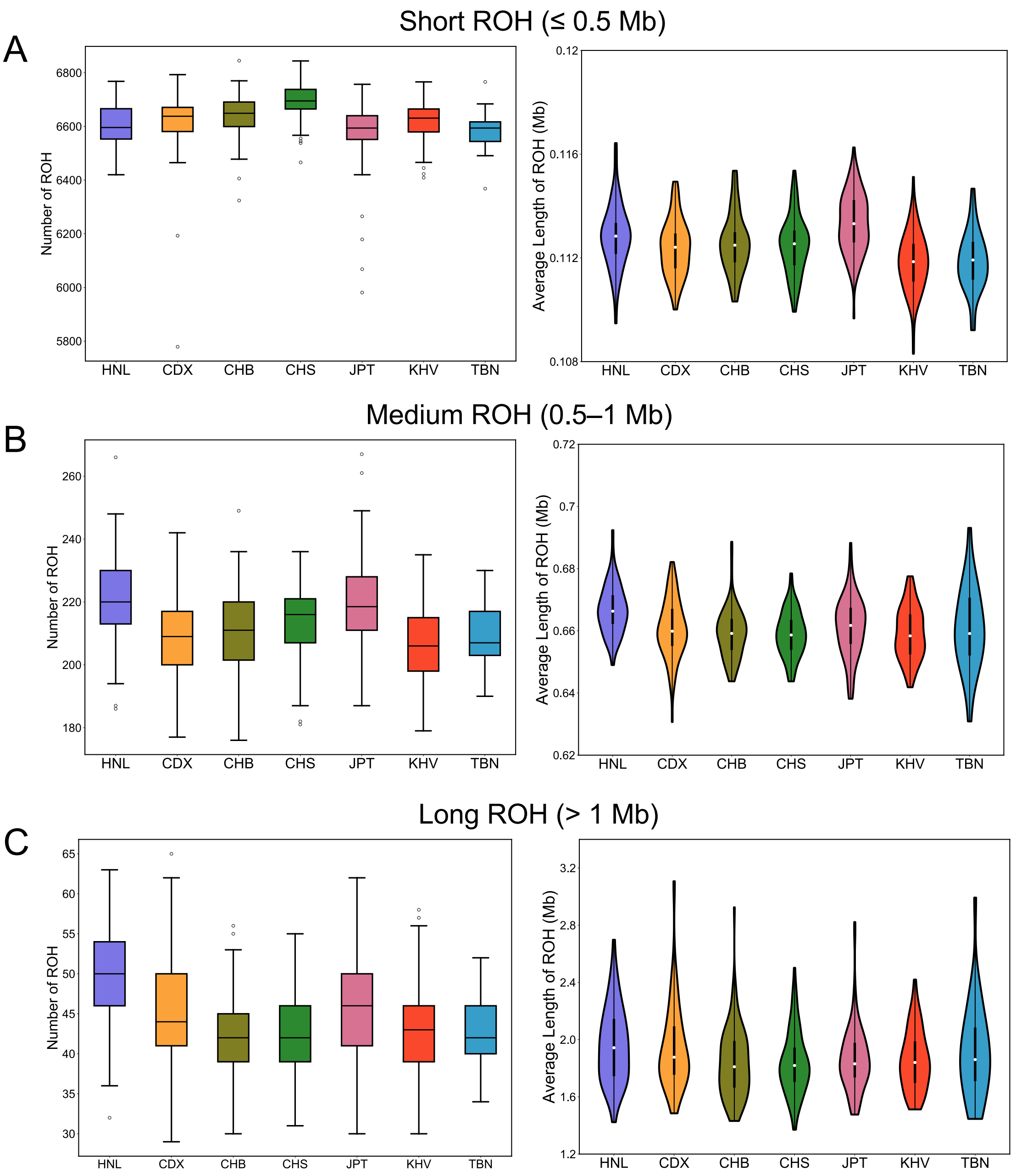

Fig. S11. ROH of HNL and other East Asian populations in the NGS Panel.

Comparison of the HNL's number of ROH and average length of ROH in (A) short (≤ 0.5 Mb), (B) medium (0.5–1Mb), and (C) long (> 1Mb) ROH categories with Tibetan and East Asian populations from the KGP dataset (1000 Genomes Project Consortium 2015) in the NGS Panel. CDX: Chinese Dai in Xishuangbanna, China; CHB: Han Chinese in Beijing, China; CHS: Han Chinese South; JPT: Japanese in Tokyo, Japan; KHV: Kinh in Ho Chi Minh City, Vietnam; TBN: Tibetan.

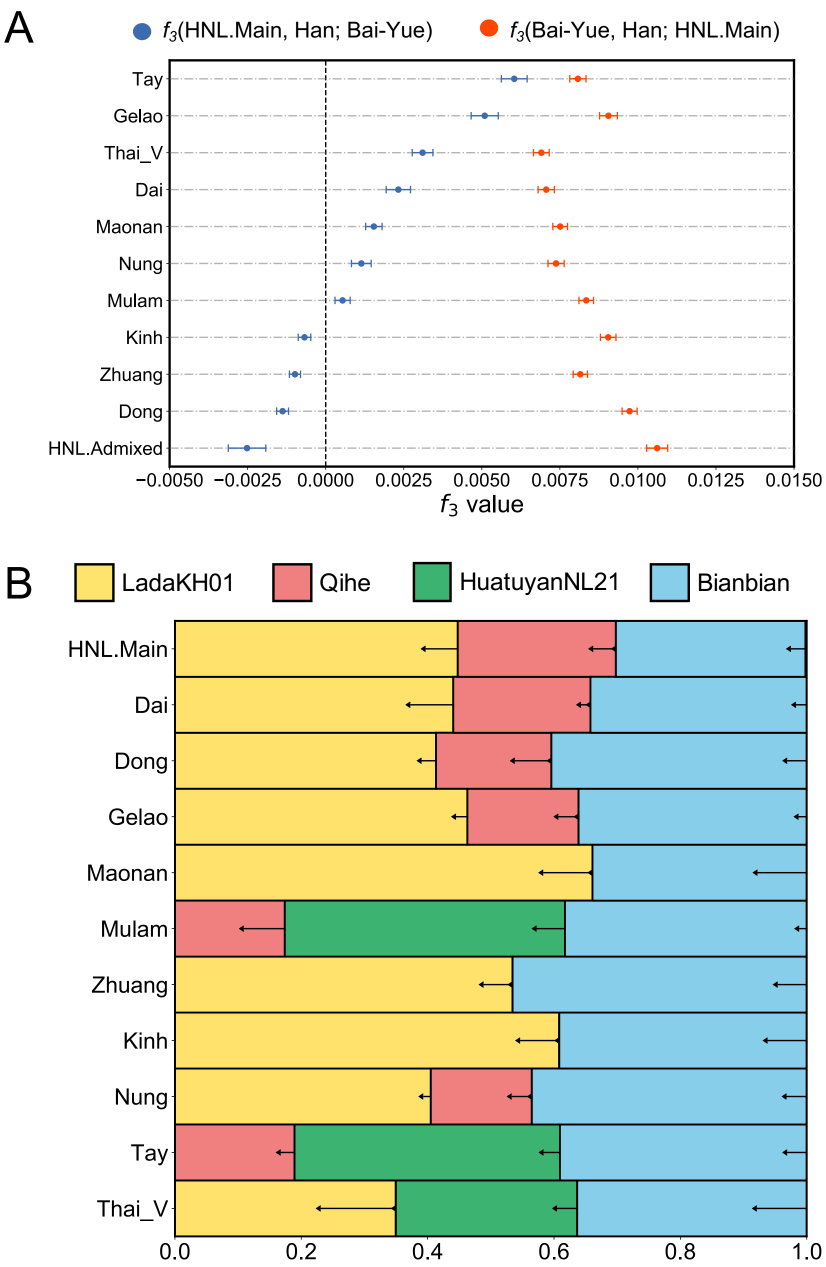

Fig. S12. Ancestral characterization of HNL.Main.

(A) Admixture f_3 statistics in the form of f_3(HNL.Main, Han; mainland Bai-Yue groups) and f_3(mainland Bai-Yue groups, Han; HNL.Main). (B) *qpAdm*-based admixture modeling for HNL.Main and mainland Bai-Yue populations, using Bianbian (ancient northern ancestry), Qihe (anceint southern ancestry), Longlin (ancient Guangxi individual related to Hòabìnhians ancestry), LadaKH01 (an ancient individual in Guangxi ~1,500 years ago), and HuatuyanNL21 (an ancient individual in Guangxi ~500 years ago) as ancestral sources. The best-fitting model with largest *P*-value was presented. The horizontal arrows represent standard deviations for coefficients of ancestry sources.

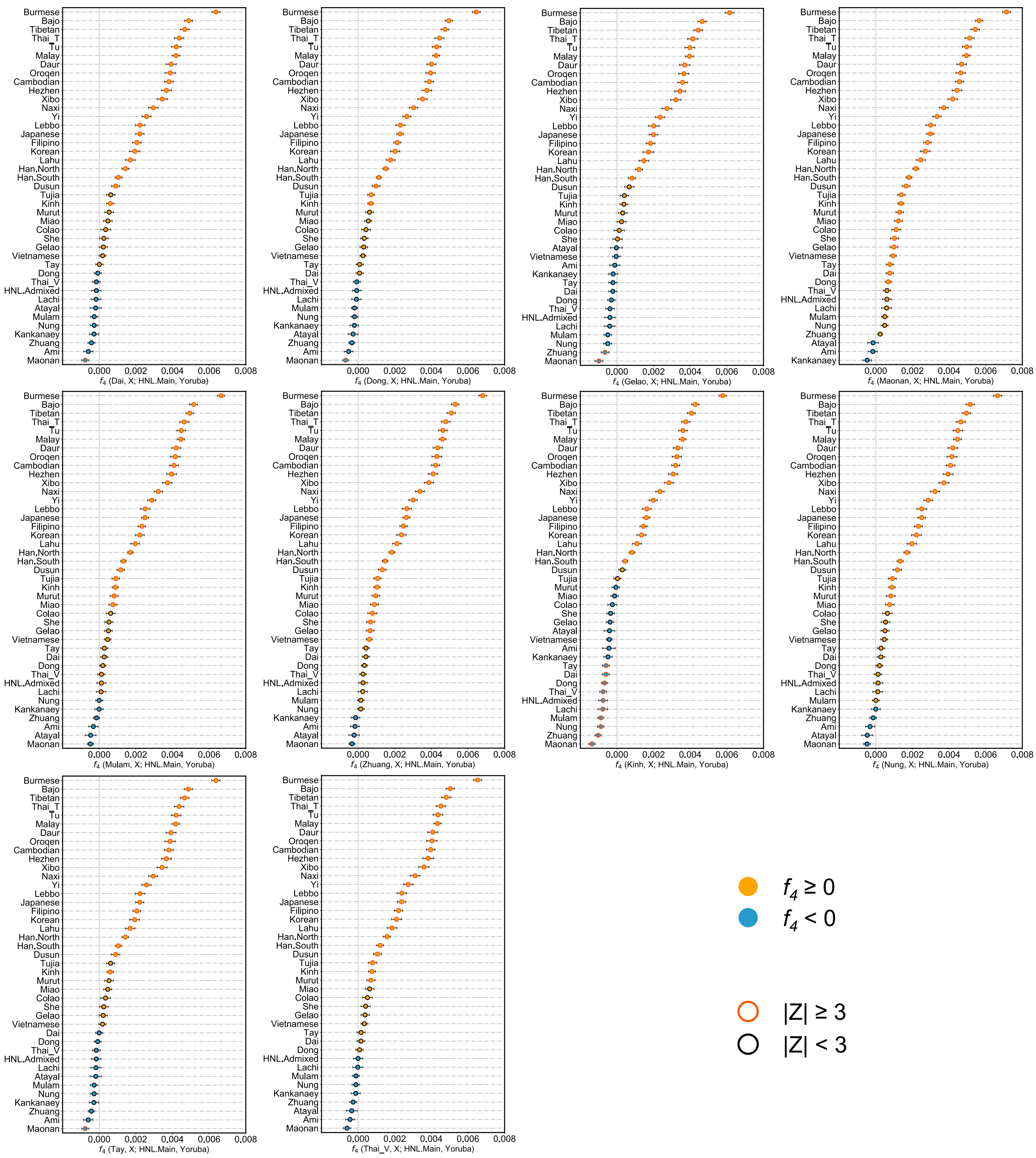

Fig. S13. Genetic affinity of HNL with East Asian and Southeast Asian populations compared to mainland Bai-Yue populations.

Genetic affinity between HNL and East Asian and Southeast Asian populations compared to mainland Bai-Yue populations, measured by f_4 statistics in the form of f_4(mainland Bai-Yue groups, X; HNL, Yoruba), where X is different East Asian and Southeast Asian populations. The positive values (orange dot) and negative values (blue dot) indicate the population show closer genetic affinity with a mainland Bai-Yue populations and HNL, respectively. The red and black edges of the dots represent absolute Z values above and below 3, respectively.

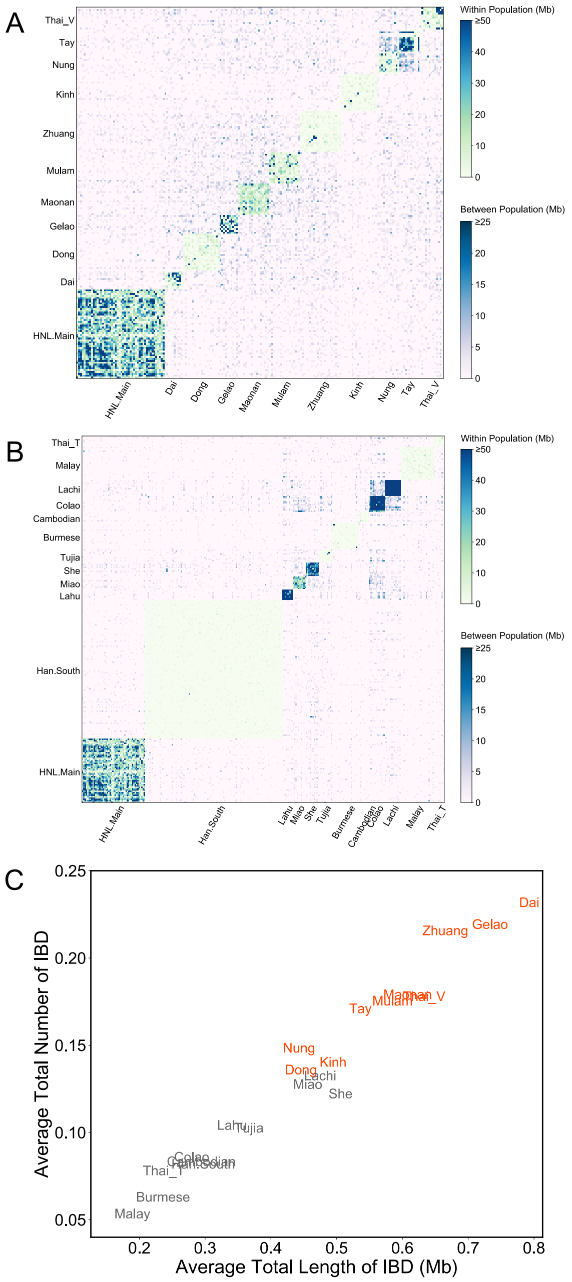

Fig. S14. IBD sharing of HNL with southern East Asian and mainland Southeast Asian populations.

A and B: Within-population and Between-population IBD sharing of HNL.Main compared to (A) mainland Bai-Yue populations and (B) other populations in southern East Asia (except Ami and Atayal) and mainland Southeast Asia, inferred by *hap-IBD* (Zhou, et al. 2020). C: Averaged total number and length of between-population IBD of HNL.Main and other southern East Asians and mainland Southeast Asians. The Bai-Yue populations were distinguished as orangered color.

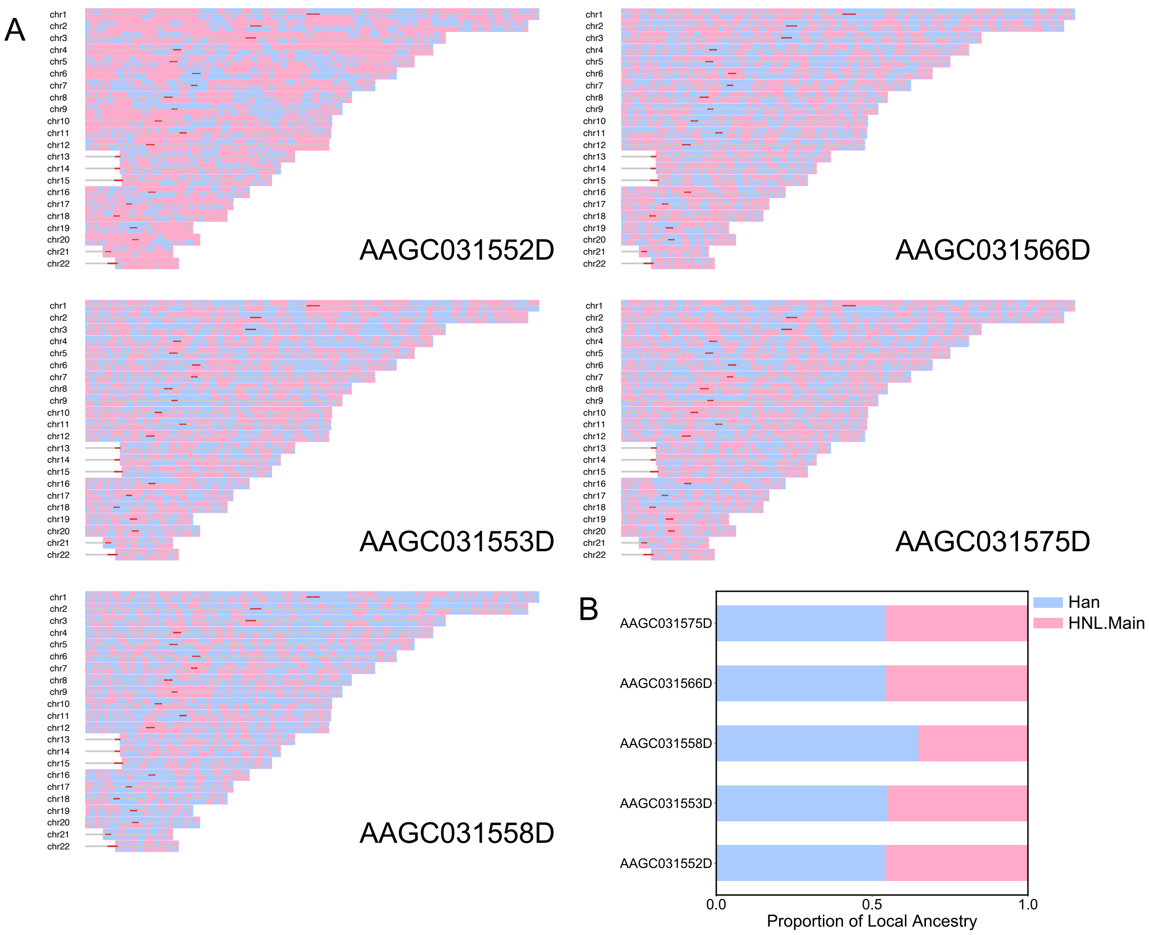

Fig. S15. Local ancestry inference of 5 HNL.Admixed individuals.

(A) Local ancestry inference results of 5 HNL.Admixed individuals performed by *RFMix* (Maples, et al. 2013), using the Han and HNL.Main as ancestral populations. (B) Summarized overall ancestry proportion of LAI results in 5 HNL.Admixed individuals.

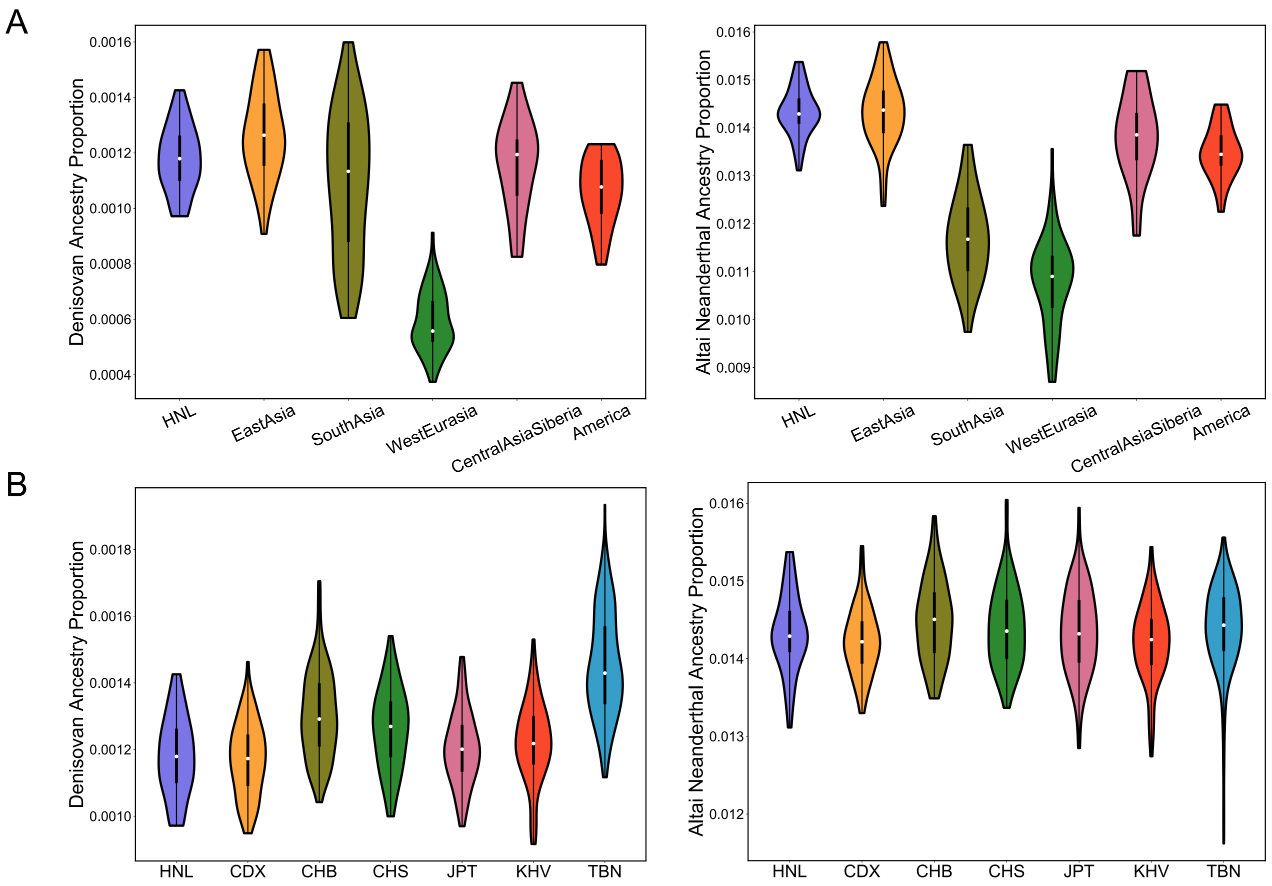

Fig. S16. Archaic introgression proportion of HNL compared with other populations. Archaic proportion of Denisovan (Meyer, et al. 2012) and Altai Neanderthal (Prufer, et al. 2014) of HNL compared with (A) non-African global populations in the SGDP dataset (Mallick, et al. 2016) under the joint-calling dataset and (B) Tibetan and East Asian populations in the KGP dataset (1000 Genomes Project Consortium 2015) under the NGS Panel. The archaic introgression was identified using *ArchaicSeeker* (Yuan, et al. 2021). CDX: Chinese Dai in Xishuangbanna, China; CHB: Han Chinese in Beijing, China; CHS: Han Chinese South; JPT: Japanese in Tokyo, Japan; KHV: Kinh in Ho Chi Minh City, Vietnam; TBN: Tibetan.

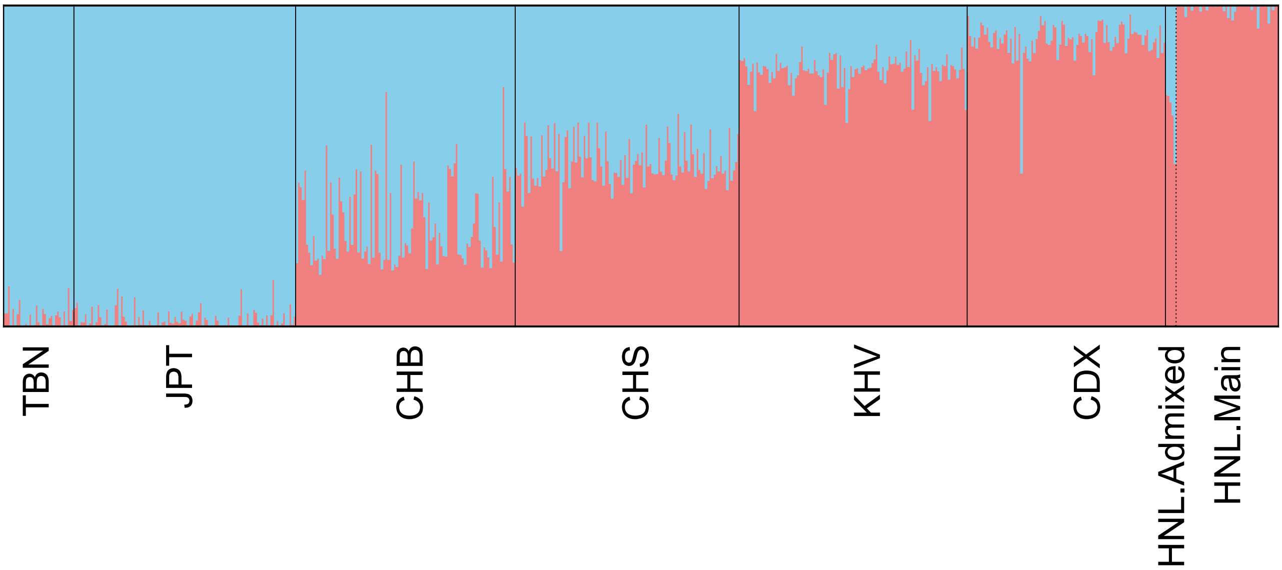

Fig. S17. Bai-Yue ancestry profile in the NGS Panel.

Unsupervised *ADMIXTURE* analysis (Alexander, et al. 2009) at K = 2 performed by 151,899 SNVs in 590 individuals of East Asian populations in the NGS Panel. The ancestry colored in red was used as Bai-Yue ancestry in these individuals. CDX: Chinese Dai in Xishuangbanna, China; CHB: Han Chinese in Beijing, China; CHS: Han Chinese South; JPT: Japanese in Tokyo, Japan; KHV: Kinh in Ho Chi Minh City, Vietnam; TBN: Tibetan.

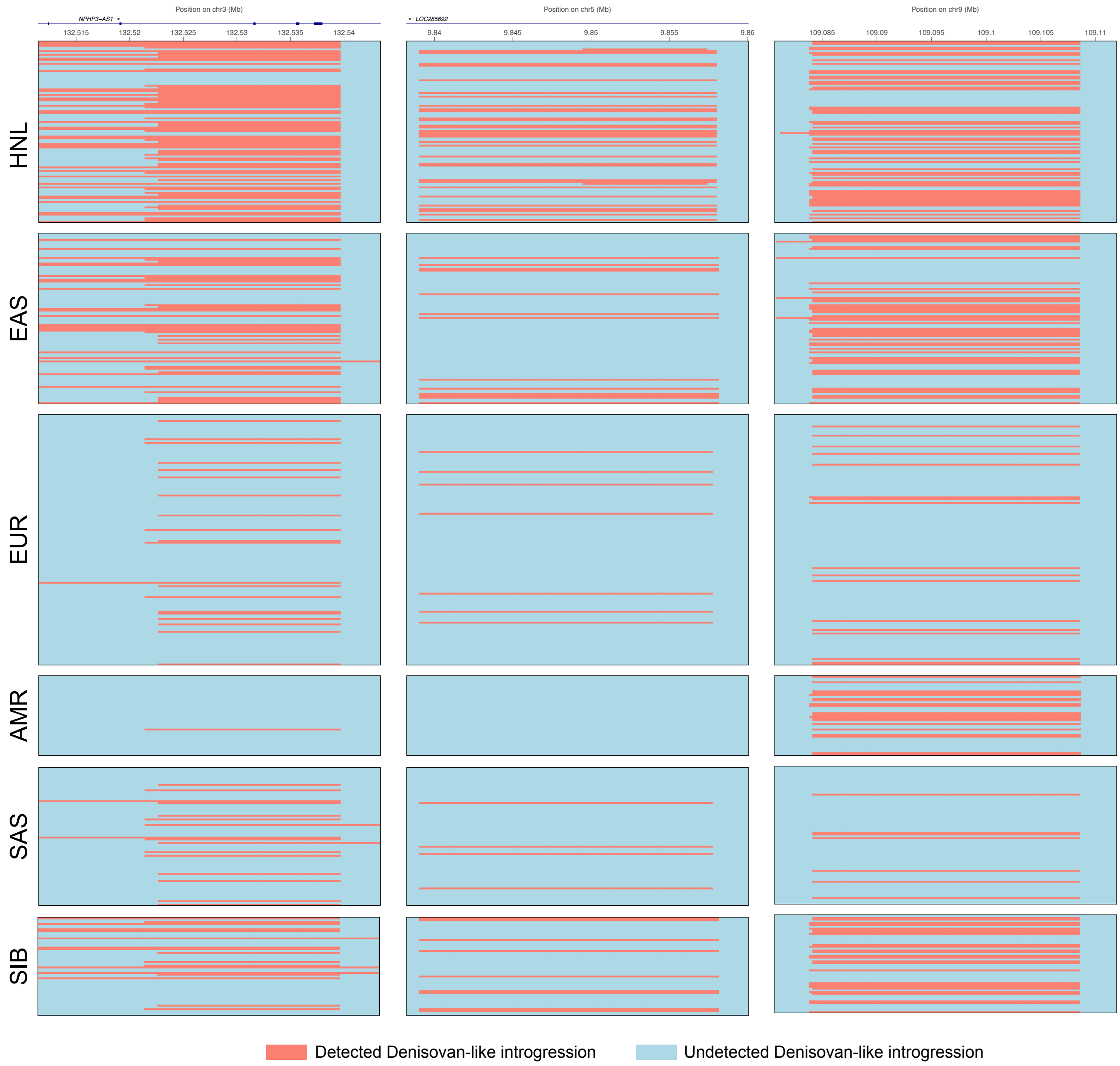

Fig. S18. Denisovan-like introgressions enriched in HNL compared to global populations. Three Denisovan-like introgressions enriched in HNL but show relatively lower frequency in other non-African global populations in the SGDP dataset (Mallick, et al. 2016). The archaic introgression was identified using *ArchaicSeeker* (Yuan, et al. 2021). The genes involved in three introgressions are *NPHP-AS1*, *LOC285692* and hypothetical gene *BC039487*, respectively. EAS: East Asian; EUR: European; AMR: American; SAS: South Asian; SIB: Central Asians and Siberian.

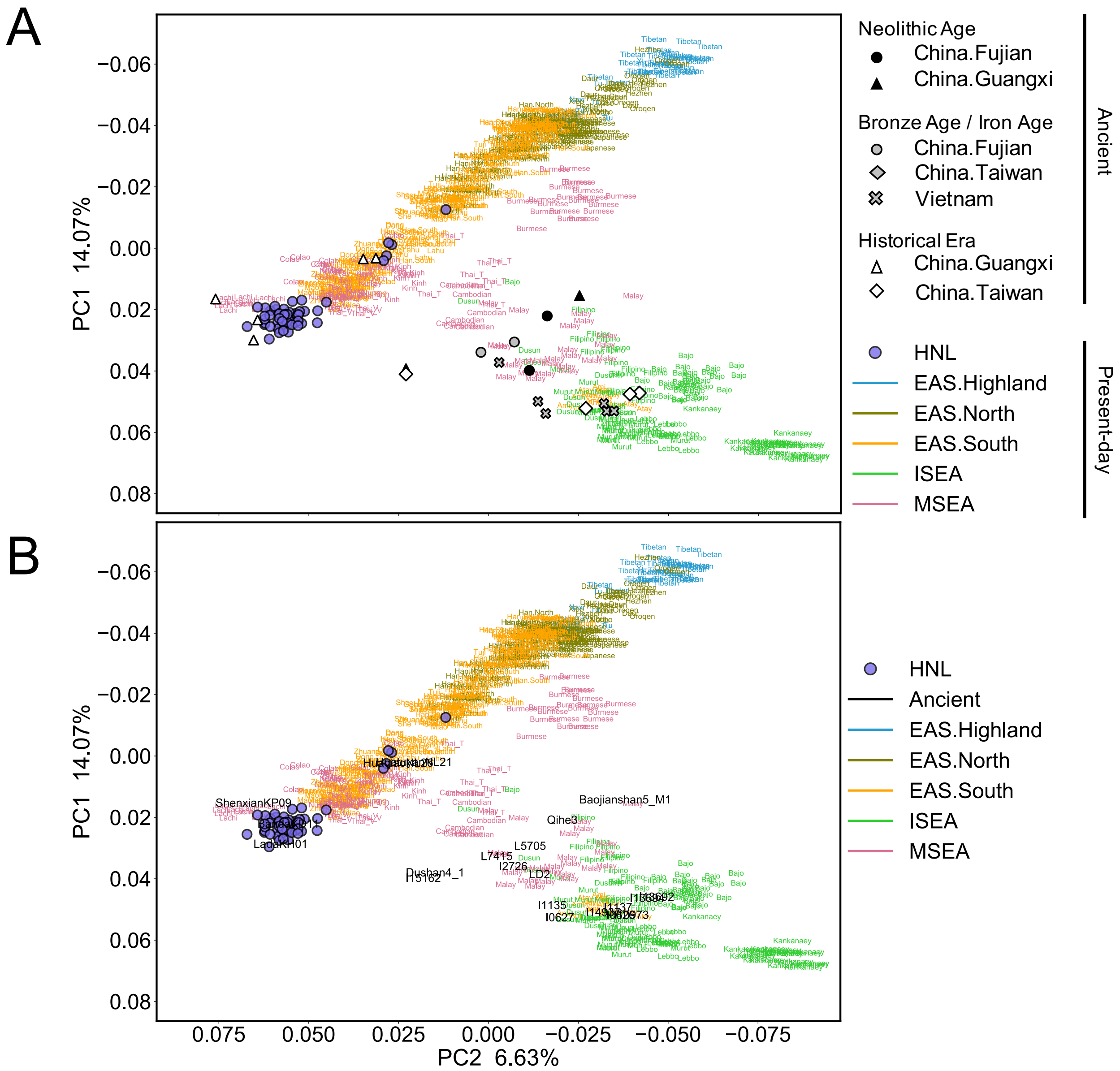

Fig. S19. PCA of ancient individuals in the context of East Asia and Southeast Asia.

21 ancient individuals from southern East Asia and mainland Southeast Asia (Lipson, et al. 2018; Yang, et al. 2020; Wang, Yeh, et al. 2021; Wang, Wang, et al. 2021) were projected onto the PCA determined for 784 present-day East Asian and Southeast Asian individuals. The ancient individuals were displayed with (A) different shapes representing different time periods and (B) sample names.

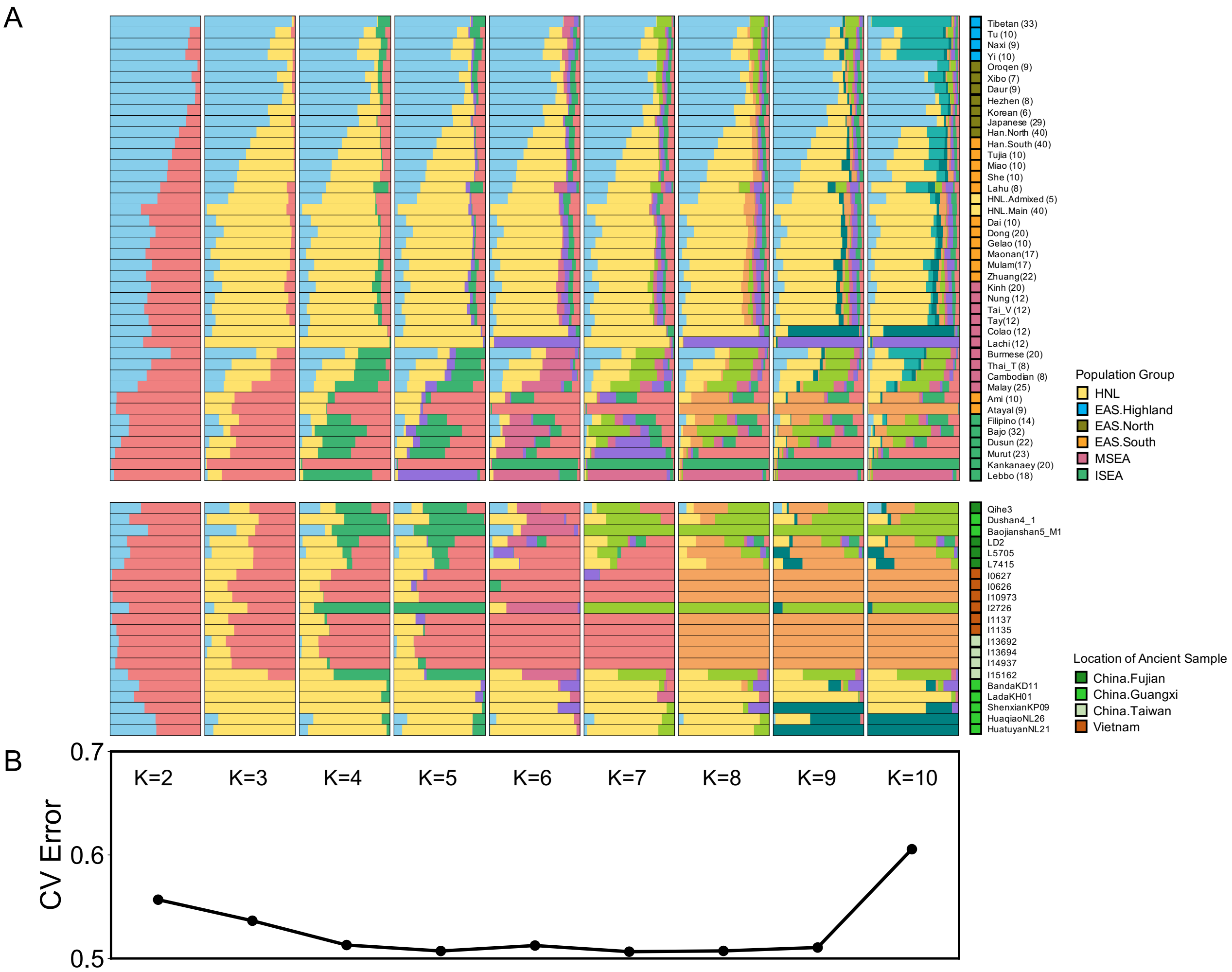

Fig. S20. Unsupervised *ADMIXTURE* of present-day and ancient individuals in East Asia and Southeast Asia.

(A) Unsupervised *ADMIXTURE* analysis (Alexander, et al. 2009) with the default parameters from K = 2 to K= 10 performed by 19,674 SNVs in 507 present-day and 21 ancient individuals (Lipson, et al. 2018; Yang, et al. 2020; Wang, Yeh, et al. 2021; Wang, Wang, et al. 2021). The maximum sample size was restricted to 40 for present-day populations with larger sample sizes. The global ancestry components for present-day and ancient samples were displayed at population and individual level, respectively. The numbers in brackets for present-day and ancient samples represent the sample sizes and approximate historical times before present, respectively. (B) CV error of current *ADMIXTURE* analysis from K = 2 to K= 10. EAS.Highland: East Asian highlanders; EAS.North: northern East Asians; EAS.South: southern East Asians; ISEA: island Southeast Asians; MSEA: mainland Southeast Asians.

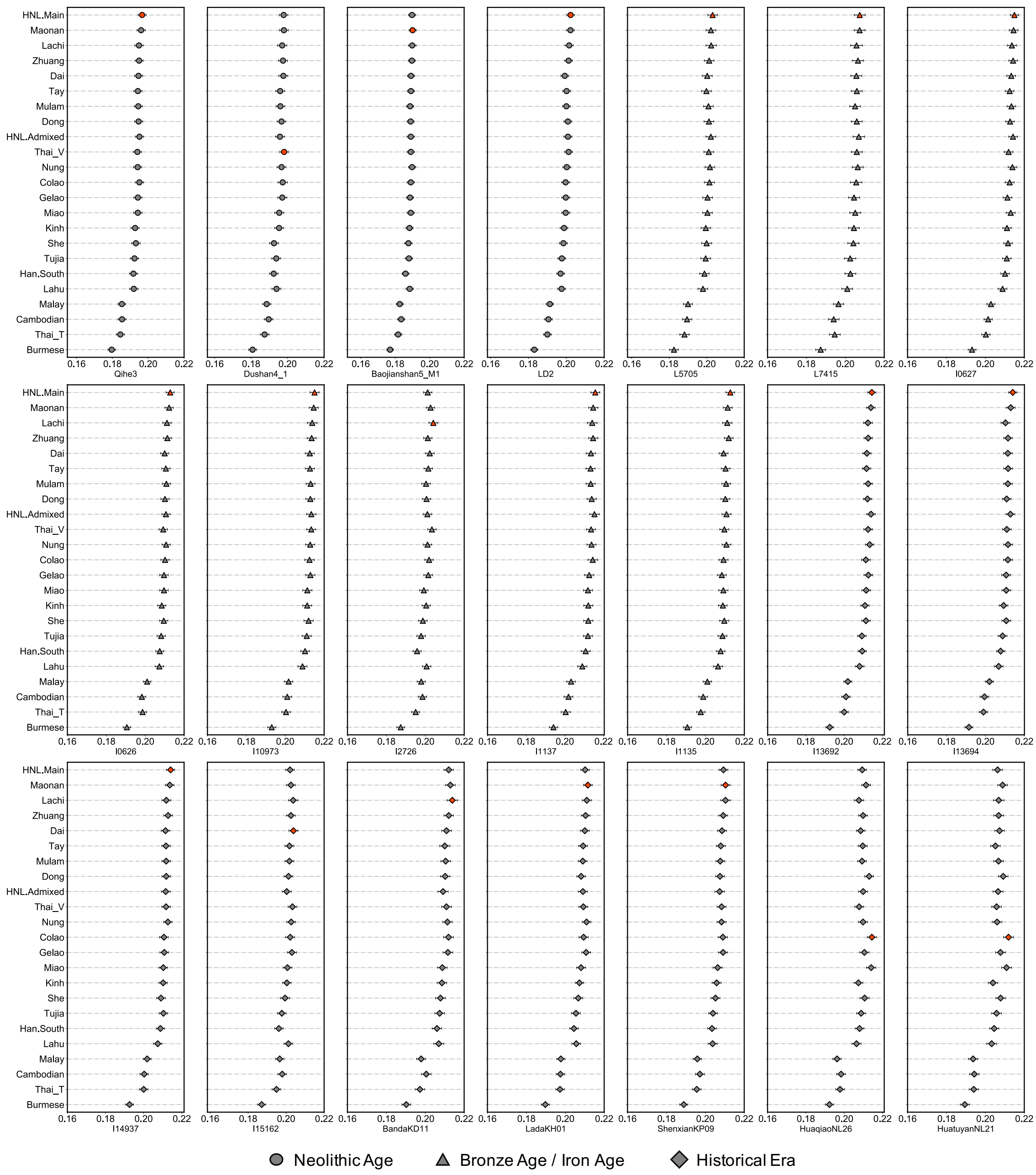

Fig. S21. Outgroup f_3 statistics comparing present-day populations to ancient individuals in the context of southern East Asia and mainland Southeast Asia.

Outgroup f_3 in the form of f_3 (X, Y; Yoruba), where X is different southern East Asian and mainland Southeast Asian populations in present day, Y is ancient individuals from southern East Asia and mainland Southeast Asia. The different shapes represent different periods in which ancient individuals were inferred.

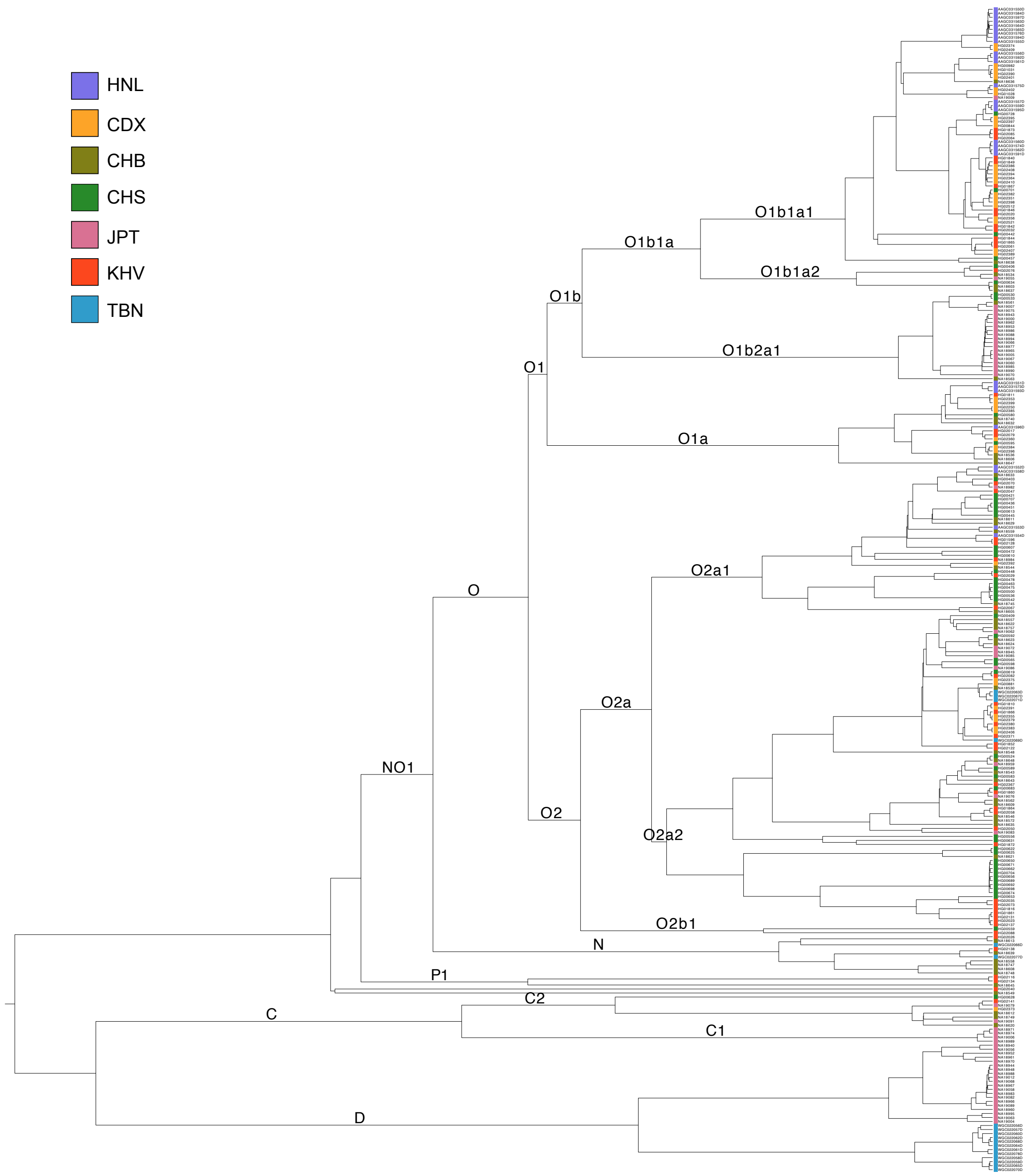

Fig. S22. Y-chromosomal phylogeny of East Asian populations.

Phylogenetic tree estimated from the Y-chromosomal sequence data of HNL, TBN, and East Asians from the KGP dataset (1000 Genomes Project Consortium 2015) using the GTR model under the strict clock and mutation rate of 7.6×10^{-10} in *BEAST* (Bouckaert, et al. 2014). Common haplogroups were displayed in the tree branches. CDX: Chinese Dai in Xishuangbanna, China; CHB: Han Chinese in Beijing, China; CHS: Han Chinese South; JPT: Japanese in Tokyo, Japan; KHV: Kinh in Ho Chi Minh City, Vietnam; TBN: Tibetan.

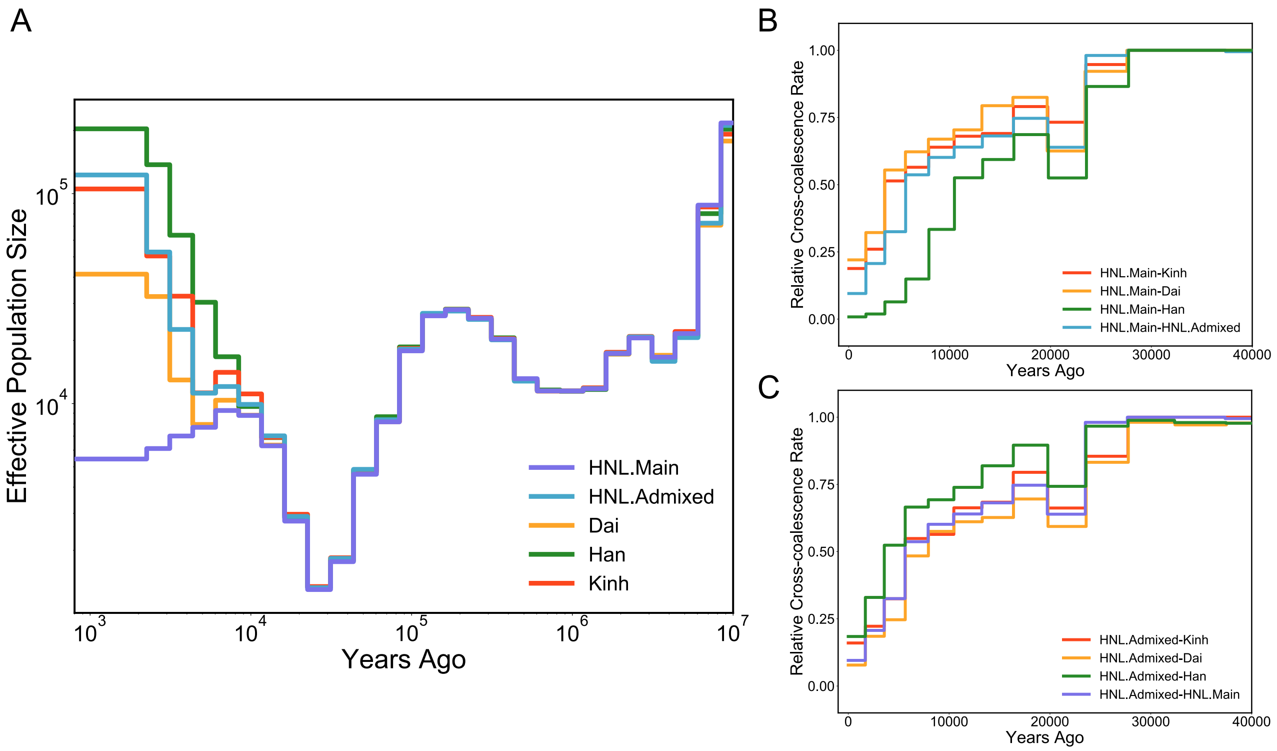

Fig. S23. Population demographic history of HNL compared to mainland Bai-Yue populations and Han.

(A) Estimate of effective population size based on genome-wide genealogies using *RELATE* (Speidel, et al. 2019) with the mutation rate of 1.25×10^{-8} per base pair per generation and 25 years per generation. (B–C) Estimates of divergence time between (B) HNL.Main and other populations and (C) HNL.Admixed and other populations, using *MSMC* (Schiffels and Durbin 2014). Divergence time between each pair of populations was evaluated using autosomal sequences of four genomes from two individuals of each population. An autosomal mutation rate with 1.25×10^{-8} per base-pair per generation and 25 years per generations were used in estimates.

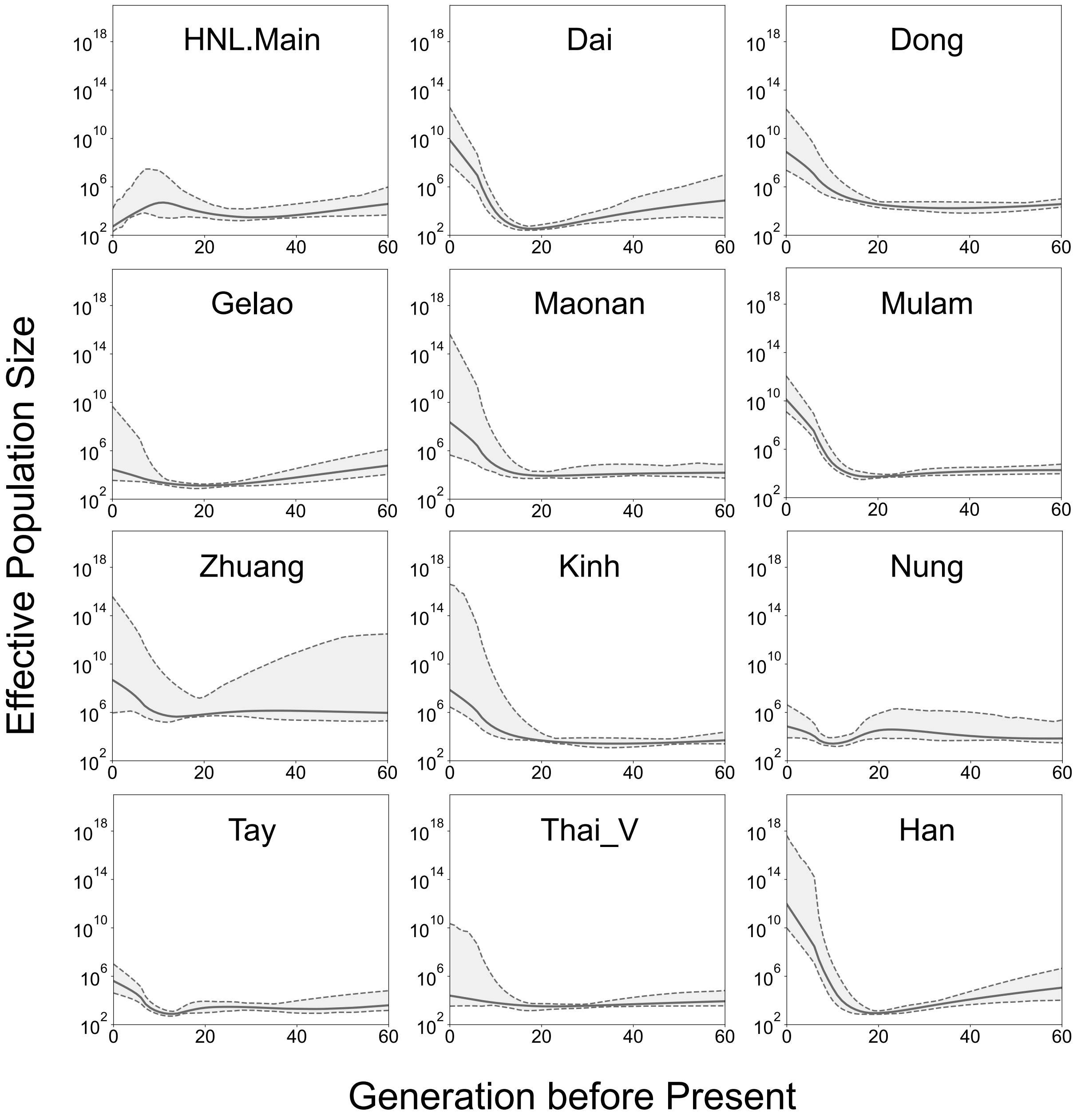

Fig. S24. Recent Population demography of HNL compared to mainland Bai-Yue populations and Han.

Estimate of recent effective population size based on IBD sharing using *IBDNe* (Browning and Browning 2015). Recent effective population size within 60 generations of Mainland Bai-Yue populations and Han were used as comparison for HNL.Main.

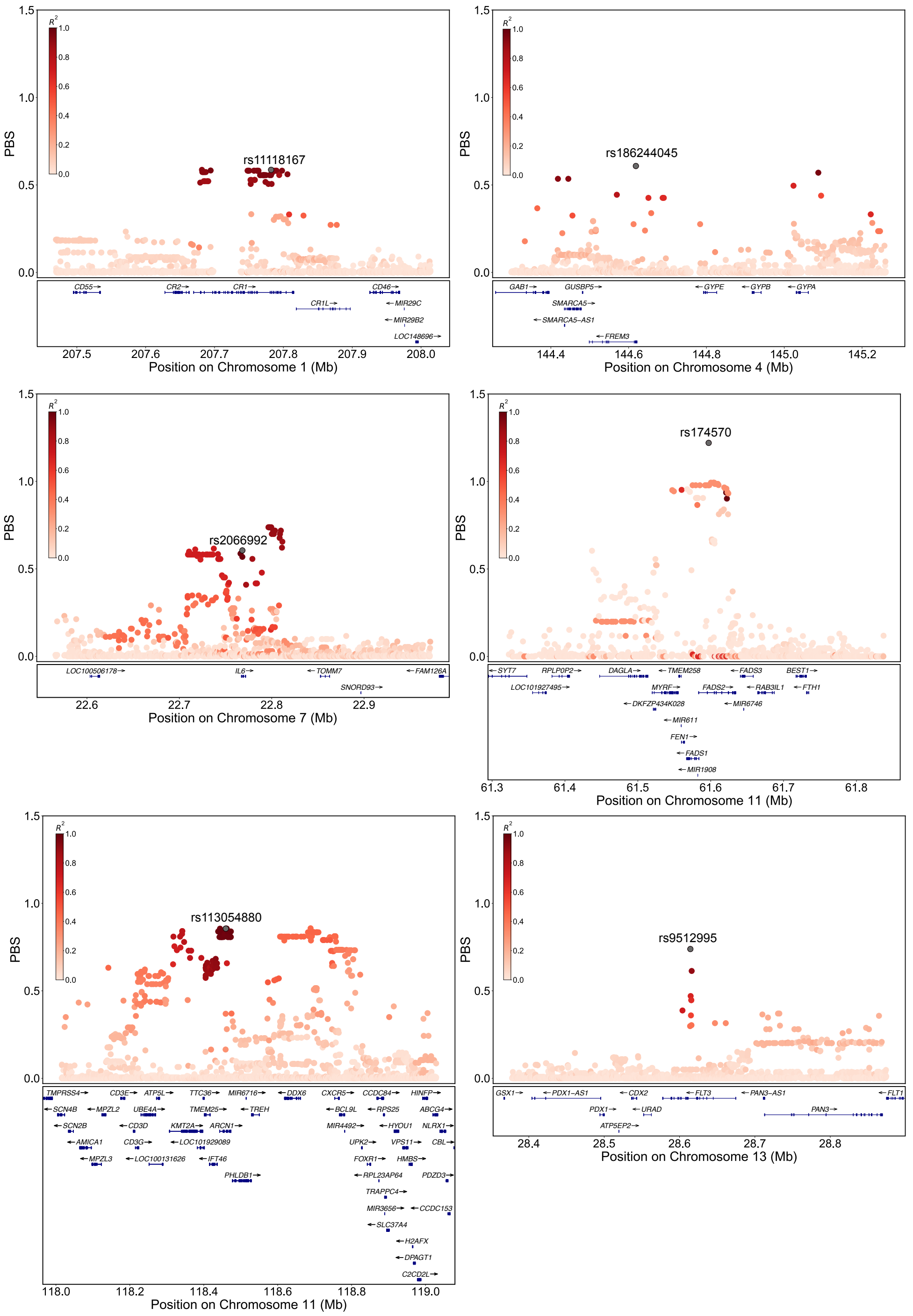

Fig. S25. Local PBS distribution and LD pattern of highlighted genes putatively under selection.

Local Manhattan plot showing PBS values spanning the highlighted genes within upstream and downstream 20 kb regions. A SNV within a gene with highest PBS value is shown in dark grey and defined as the top SNV, whereas other SNVs are colored according to pairwise linkage disequilibrium with the top SNV based on the HNL-Han-CEU trio dataset. CEU: Utah residents with northern and western European ancestry.

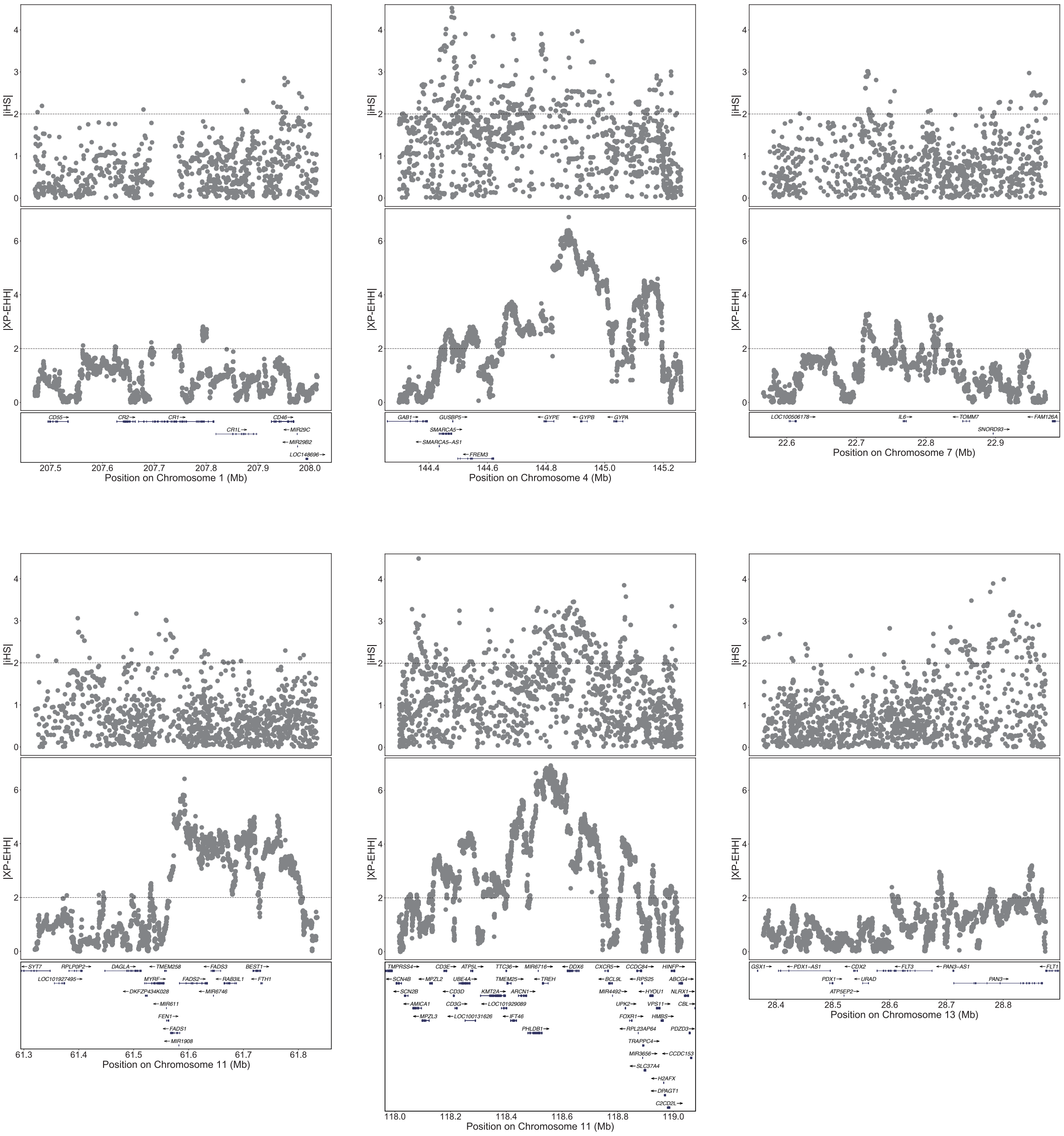

Fig. S26. Local iHS and XP-EHH distribution of highlighted genes putatively under selection.

Local Manhattan plot showing absolute iHS and XP-EHH values spanning the highlighted genes within upstream and downstream 20 kb regions.

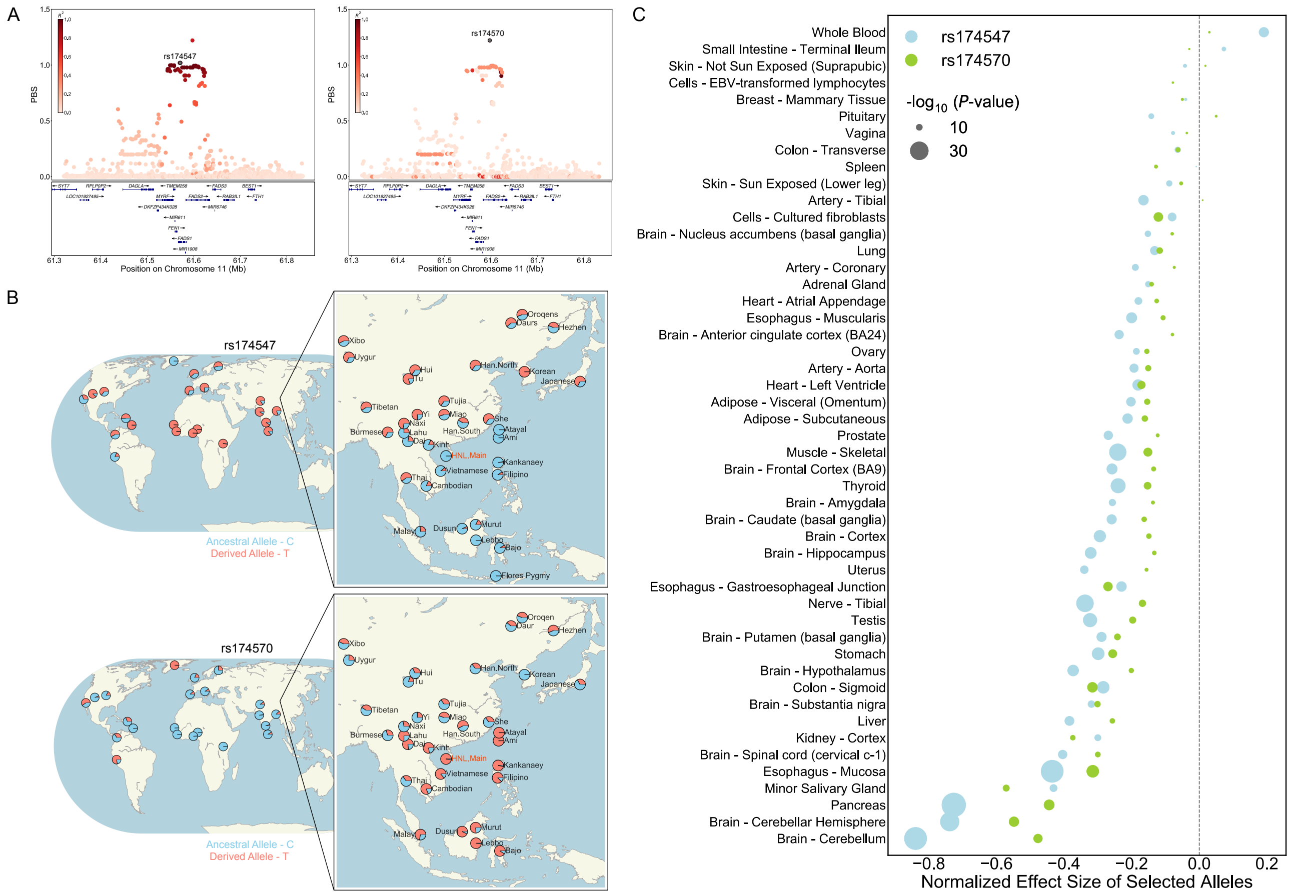

Fig. S27. Population prevalence and functional signatures of concerned variants in FADS locus.

(A) Local distribution of PBS values spanning the FADS locus within upstream and downstream 20 kb regions. rs174547 (left) and rs174570 (right) are shown in dark grey, whereas other SNVs are colored according to pairwise linkage disequilibrium with rs174547/rs174570 based on the HNL-Han-CEU trio dataset. CEU: Utah residents with northern and western European ancestry. (B) Population prevalence of the rs174547 (upper) and rs174570 (lower) based on the *PGG.SNV* database (Zhang, et al. 2019) and Southeast Asian populations from Mörseburg et al (Morseburg, et al. 2016). (C) Associations between *FADS1* expression and the rs174547 (skyblue) and rs174570 (yellowgreen) based on multi-tissue eQTL of the GTEx dataset (GTEx Consortium 2013). The x-axis represents normalized effect size, y-axis represents different tissues, and point sizes represent significance. Selected allele signifies the allele nearly fixed in HNL.

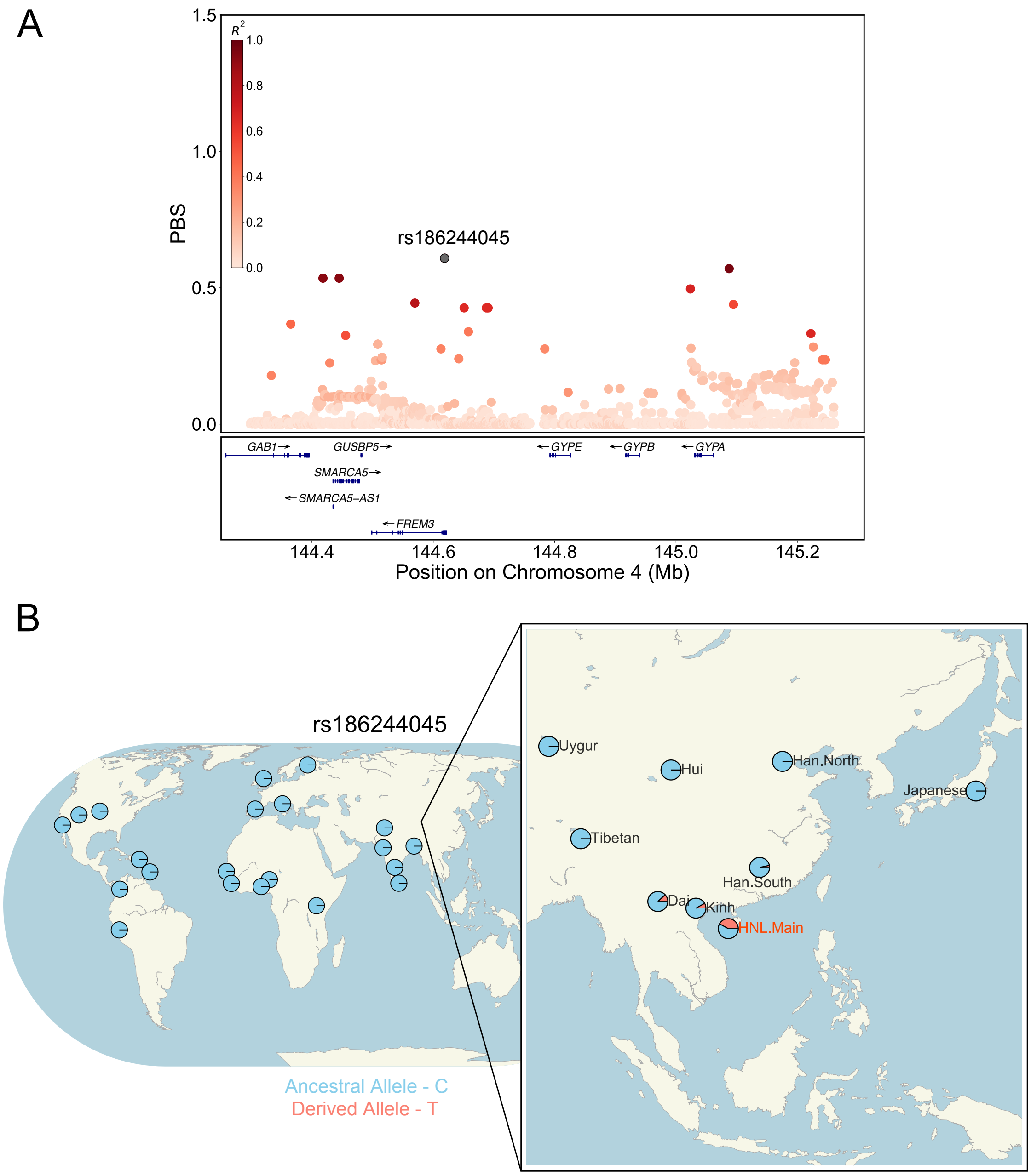

Fig. S28. Local PBS distributions and global population prevalence of Bai-Yue-specific adaptive variant rs186244045 within *FREM3*.

(A) Local distribution of PBS values spanning the malaria-related *FREM3* within upstream and downstream 20 kb regions. The specific adaptive variant of Bai-Yue populations (rs186244045) is shown in dark grey, whereas other SNVs are colored according to pairwise linkage disequilibrium with this variant based on the HNL-Han-CEU trio dataset. (B) Population prevalence of the rs186244045 based on the *PGG.SNV* database (Zhang, et al. 2019). CEU: Utah residents with northern and western European ancestry.

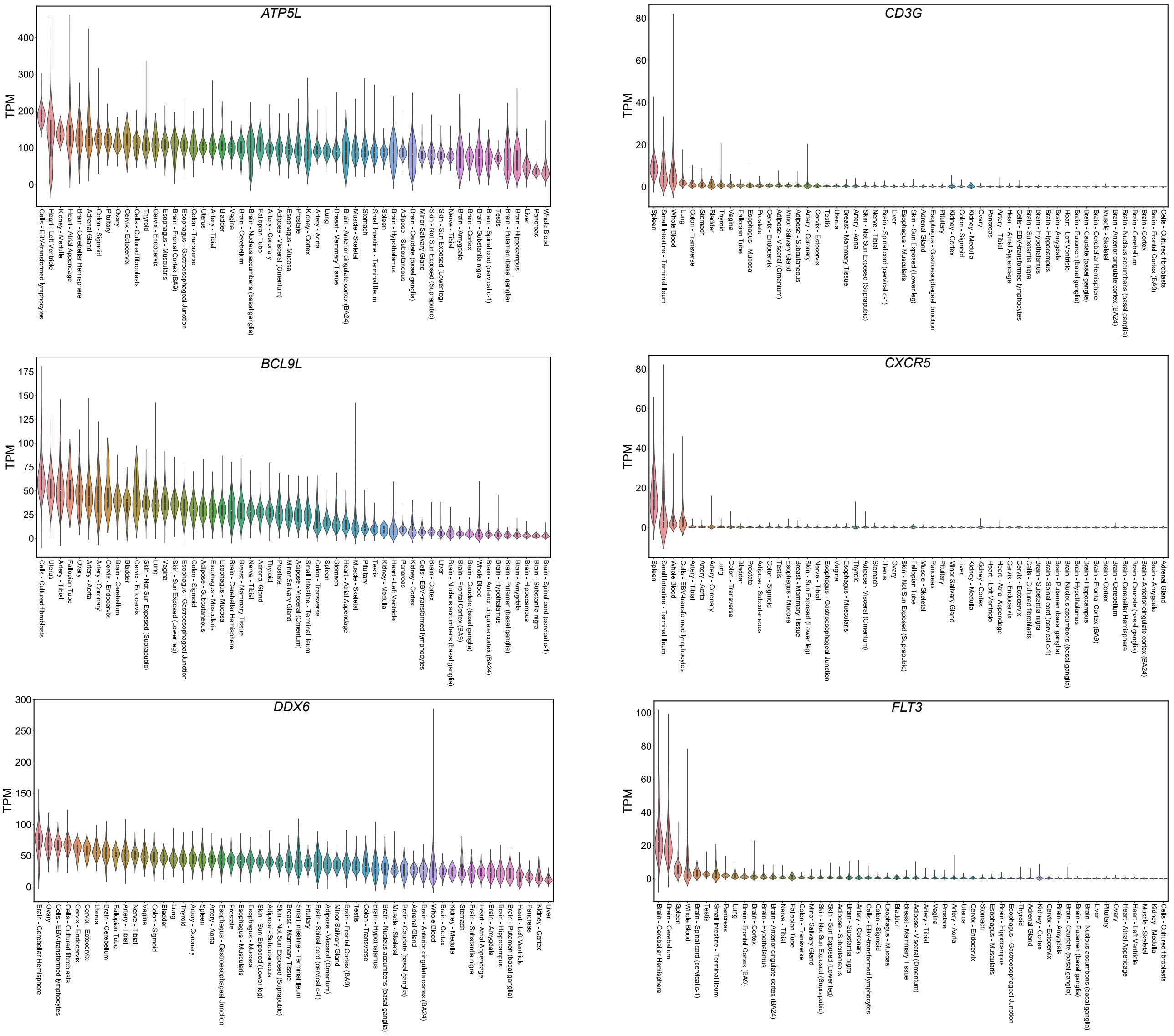

Fig. S29. Multi-tissue expression of genes under selection related to hematopoietic function and occurrence of B cell lymphomas.

Multi-tissue expression based on the GTEx dataset (GTEx Consortium 2013) for 6 genes under selection related to hematopoietic function and occurrence of B cell lymphomas. The x-axis represents different tissues and y-axis represents gene expression level in Transcripts Per Million (TPM).

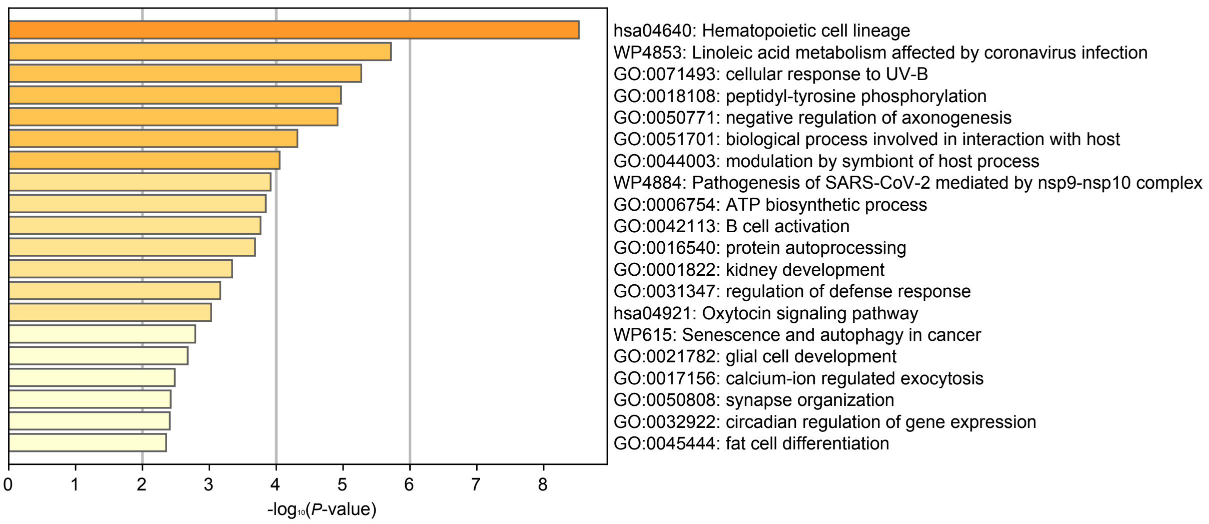

Fig. S30. Functional terms enriched from gene set consisting of genes with strong selection signals.

Functional enrichment of HNL selection signals performed by *metascape* (Zhou, et al. 2019). The input gene set consist of genes with variants of PBS values in the top 0.005% percentile. The top 20 functional categories with $-\log_{10}(P\text{-value}) \geq 2$ were displayed as enriched terms.

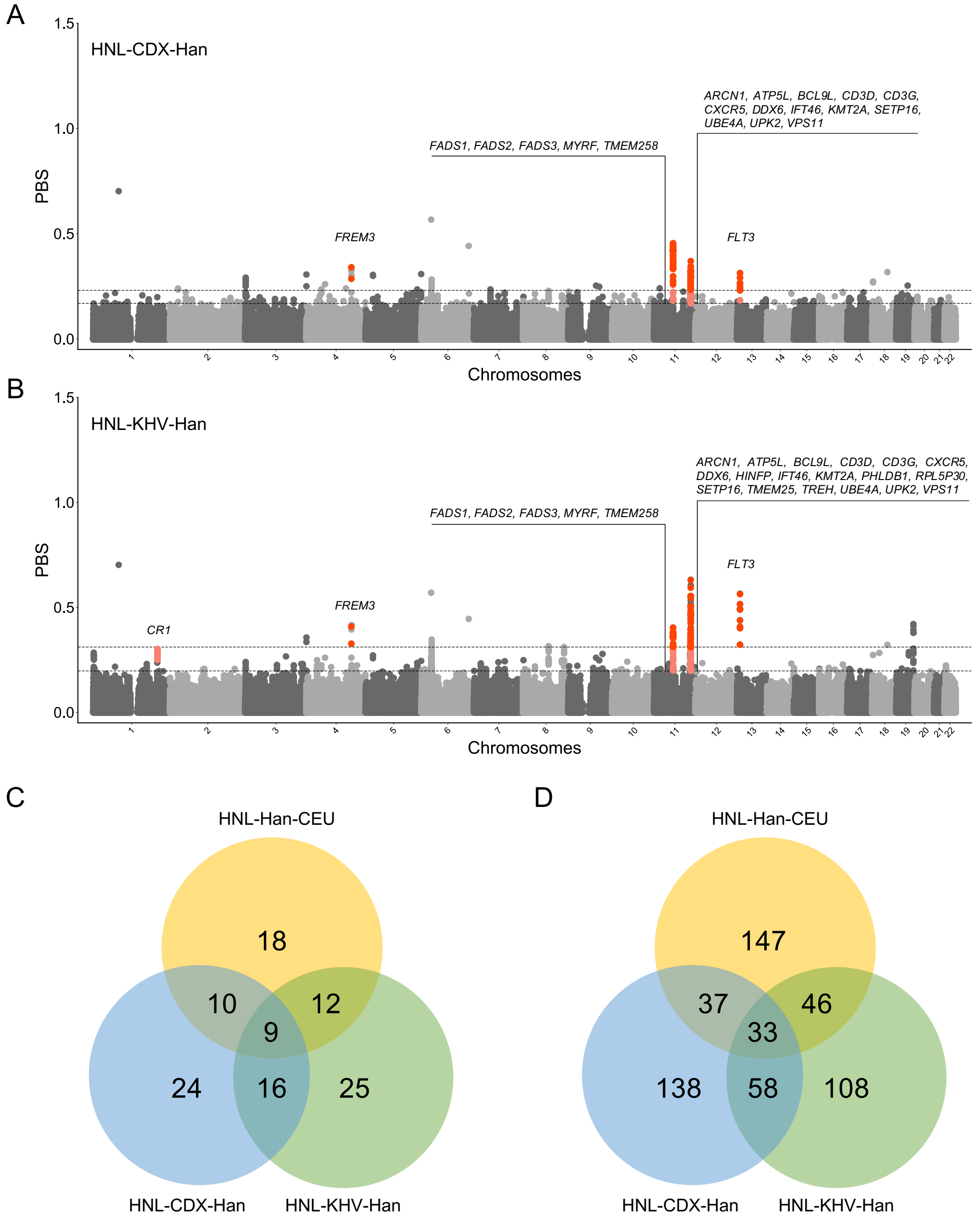

Fig. S31. Differentiated adaptive signals within Bai-Yue lineage.

(A–B) Genome-wide scan of differentiated adaptive signals between island (HNL) and mainland (CDX and KHV) Bai-Yue populations using the (A) HNL-CDX-Han and (B) HNL-KHV-Han trios. (C–D): Venn plot showing overlaps of adaptive signals over (C) 99.999th and (D) 99.995th percentiles among the HNL-Han-CEU, HNL-CDX-Han, and HNL-KHV-Han trios. CDX: Chinese Dai in Xishuangbanna, China; KHV: Kinh in Ho Chi Minh City, Vietnam. CEU: Utah residents with northern and western European ancestry.

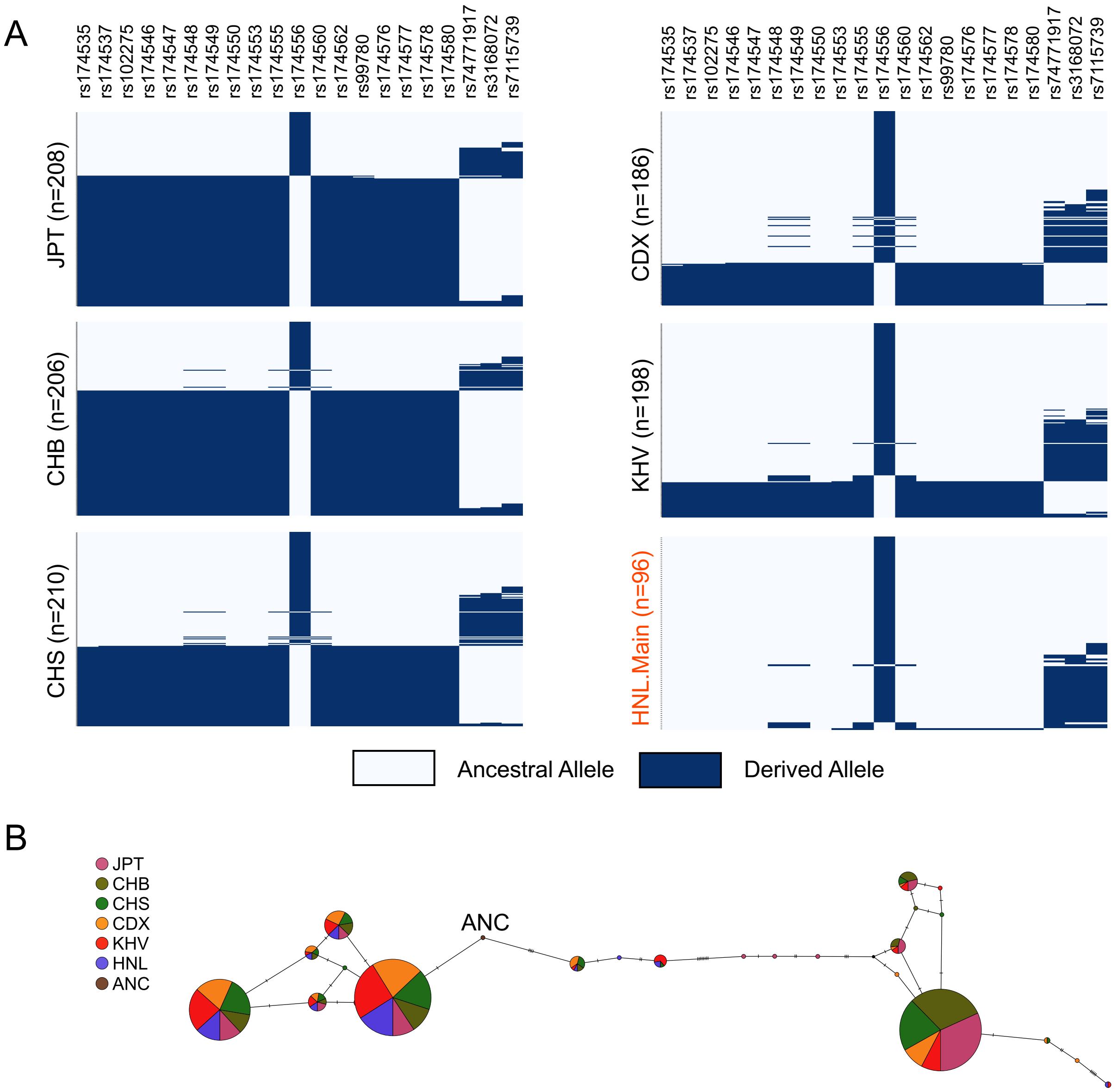

Fig. S32. Haplotype patterns of FADS region among East Asian populations.

(A) Haplotype patterns stratified by East Asian populations. The haplotype pattern was defined by a previous study of Tucci et al (Tucci, et al. 2018). Populations and corresponding sample sizes are labeled in y-axis. Columns represent SNVs and rows represent individuals for each population. Dark blue indicates derived alleles and light blue indicates ancestral alleles. (B) Haplotype network of East Asian populations of FADS region, constructed by *popart* (Leigh and Bryant 2015). JPT: Japanese in Tokyo, Japan; CHB: Han Chinese in Beijing, China; CHS: Han Chinese South; CDX: Chinese Dai in Xishuangbanna, China; KHV: Kinh in Ho Chi Minh City, Vietnam.

Table S1. Novel variants identified from 55 whole-genome sequenced HNL samples in this study.

Functional categories were annotated for novel variants of 55 whole-genome sequenced HNL samples in this study. The novel variants were defined as SNVs that are not included in the *dbSNP* database (Sherry, et al. 2001) v154 and were annotated by *Variant Effect Predictor* (McLaren, et al. 2016).

Category		Count
Loss-of-function	missense variant (SIFT < 0.05 and PolyPhen > 0.446)	650
	start lost	5
	stop gained	48
	stop lost	1
	splice acceptor variant	4
	splice donor variant	8
missense variant (SIFT ≥ 0.05 and PolyPhen ≤ 0.446)		1317
synonymous variant		890
Non-coding	non-coding transcript exon variant	936
	mature miRNA variant	1
	intron variant	190855
UTR	3 prime UTR variant	2590
	5 prime UTR variant	540
intergenic variant		126831
upstream gene variant		13576
downstream gene variant		17374
regulatory region variant		6299
TF binding site variant		109
Total		362034

Table S3. NRY and mtDNA haplogroups of 55 whole-genome sequenced HNL samples in this study.

SampleID	Subgroup	Mt haplogroup	NRY haplogroup
AAGC031543D	HNL.Main	F3a1	-
AAGC031544D	HNL.Main	M7b1a1	-
AAGC031545D	HNL.Main	B4b1a2a	-
AAGC031546D	HNL.Main	B5a1b1	-
AAGC031547D	HNL.Main	M7b2	-
AAGC031548D	HNL.Main	M7c2a	-
AAGC031549D	HNL.Main	M7c1b2b	-
AAGC031550D	HNL.Main	R9b1a3	O1b1a1a1b2a1a1b
AAGC031551D	HNL.Main	F2e	O1a1a2a1a
AAGC031552D	HNL.Admixed	G2a4	O2a1b1a1a1a1g1
AAGC031553D	HNL.Admixed	M7b1a1b	O2a1b1a1a1a1d
AAGC031554D	HNL.Main	M7b1a1e	O2a1b1a1a1a1c2
AAGC031555D	HNL.Main	R9c1b1	O1b1a1a1b2a1a1b
AAGC031556D	HNL.Main	R9b2	O1b1a1a1b1b
AAGC031557D	HNL.Main	M8a2a1	O1b1a1a1a1b1a
AAGC031558D	HNL.Admixed	B4c1b2a2	O2a1b1a1a1j
AAGC031559D	HNL.Main	B4a1d	O1b1a1a1a1b1a
AAGC031560D	HNL.Main	M7b1a1	O1b1a1a1a1a1a1
AAGC031561D	HNL.Main	M7c1b2b	O1b1a1a1b1b
AAGC031562D	HNL.Main	B4h1	O1b1a1a1a1a1a1
AAGC031563D	HNL.Main	F1a1d	O1b1a1a1b2a1a1b
AAGC031564D	HNL.Main	R11b1	O1b1a1a1b2a1a1b
AAGC031565D	HNL.Main	M8a2a1	O1b1a1a1b2a1a1b
AAGC031566D	HNL.Admixed	F3a1	-
AAGC031567D	HNL.Main	B5b	-
AAGC031568D	HNL.Main	B5a1	-
AAGC031569D	HNL.Main	M7c2a	-
AAGC031570D	HNL.Main	F1a1a	-
AAGC031571D	HNL.Main	F1a1d	-
AAGC031572D	HNL.Main	M7c1b2b	-
AAGC031573D	HNL.Main	D4b1b2	O1a1a2a1a
AAGC031574D	HNL.Main	R11b1	O1b1a1a1a1a1a1
AAGC031575D	HNL.Admixed	M7c1a	O1b1a1a1b1a1a1a1a
AAGC031576D	HNL.Main	M8a2a1	O1b1a1a1b2a1a1b
AAGC031577D	HNL.Main	M7b1a1	-
AAGC031578D	HNL.Main	F2b1	-
AAGC031579D	HNL.Main	B4a1d	-
AAGC031580D	HNL.Main	M12a1a	-

SampleID	Subgroup	Mt haplogroup	NRY haplogroup
AAGC031581D	HNL.Main	B4g2	-
AAGC031582D	HNL.Main	M7c2	-
AAGC031583D	HNL.Main	M7c2	-
AAGC031584D	HNL.Main	M8a2a1	O1b1a1a1a1b2a1a1b
AAGC031585D	HNL.Main	B5a1	-
AAGC031586D	HNL.Main	B4g1a	-
AAGC031587D	HNL.Main	B4b1a2a	-
AAGC031588D	HNL.Main	M12a1a	-
AAGC031589D	HNL.Main	R9b1	-
AAGC031590D	HNL.Main	F4a	-
AAGC031591D	HNL.Main	M7c2a	O1b1a1a1a1a1a1
AAGC031592D	HNL.Main	M8a2a1	O1b1a1a1a1b1b
AAGC031593D	HNL.Main	F2	O1a1a2a1a
AAGC031594D	HNL.Main	M7c1b2b	O1b1a1a1a1b2a1a1b
AAGC031595D	HNL.Main	M7c1a	O1b1a1a1a1a1b1a
AAGC031596D	HNL.Main	B4b1a2a	O1a1a1b1b
AAGC031597D	HNL.Main	F1a1d	O1b1a1a1a1b2a1a1b

Table S10. Estimated age of NRY haplogorups in East Asian populations used for Y-chromosomal phylogeny construction.

Age estimates with 95% confidence interval (CI) of NRY haplogroups in Y-chromosomal phylogeny using HNL, Tibetan and other East Asian populations in the KGP dataset (1000 Genomes Project Consortium 2015).

Haplogroup	Alternate Name	Age	95% CI	
			Lower	Upper
C	C-M130	43250	41105	45851
C1a1a1	D-M174	1441	1258	1970
C2b	C-L1373	30680	29210	32059
D1a	D-F6251	30063	26548	32728
D1a1	D-M15	9351	8238	10056
D1a2a	D-P47	6579	4295	7729
NO1	NO-M214	48599	46342	50858
N1	N-CTS3750	18275	17472	19710
O	O-M175	40691	39021	42671
O1	O-F265	38994	36898	40608
O1a1	O-B384	13415	12434	15094
O1a1a	O-M307.1	8418	7704	9286
O1a1a1	O-F446	6832	6298	7685
O1b1a	O-M1740	35821	33542	38350
O1b1a1a	O-M95	25742	23683	27555
O1b1a1a1a1	O-F2924	10998	10282	11651
O1b1a1a1a1a1	O-F2758	8980	8435	9397
O1b1a1a1a1a1a1	O-Z24089	3849	3030	4597
O1b1a1a1a1a1a1a1	O-CTS2022	2663	2282	2972
O1b1a1a1a1a1a1a1a	O-F1399	2511	2156	2712
O1b1a1a1a1a1a1a1a1	O-F2415	1964	1734	2216
O1b1a1a1a1a1b	O-F2890	3161	2616	4247
O1b1a1a1a1b	O-CTS5854	8558	8024	8978
O1b1a1a1a1b1	O-Z23810	8353	7727	8810
O1b1a1a1a1b1a	O-CTS7399	2258	1655	2735
O1b1a1a1a1b1b	O-CTS651	3094	2670	3703
O1b1a1a1a1b2	O-F4229	2828	2025	3437
O1b1a1a1b	O-F789	10530	7903	10960
O1b1a1b1a1	O-F3357	3123	2224	4012
O1b1a2a	O-F993	11810	9912	13611
O1b2a1	O-CTS9259	8541	7903	10960
O1b2a1a	O-F1204	5450	4575	6546
O2	O-M122	36356	34374	38209
O2a	O-M324	30665	28733	32629

Haplogroup	Alternate Name	Age	95% CI	
			Lower	Upper
O2a1	O-L127.1	20409	19126	21740
O2a1a1	O-F2159	12033	10762	12801
O2a1b	O-M164	16370	14744	17573
O2a2	O-P201	29484	27581	31491
O2a2a	O-M188	22412	20483	24689
O2a2a1a	O-CTS445	15485	13791	17994
O2a2a1a2a1	O-F1276	6275	5165	7471
O2a2b	O-P164	23348	21718	24741
O2a2b1a	O-F450	19884	18603	21094
O2a2b2a1	O-N7	15149	14091	16299
O2b1	O-F1150	20122	17942	21713
P1	P-M45	40244	37094	42089

The following supplementary tables were provided as separate spreadsheets:

Table S2. Information of present-day samples used in this study.

Table S4. NRY haplogroup frequencies of East Asian and Southeast Asian populations.

Table S5. MtDNA haplogroup frequencies of East Asian and Southeast Asian populations.

Table S6. Potential admixture of HNL.Main inferred by f_3 statistics.
The f_3 tests were performed for Bai-Yue populations in the form of f_3(X, Y; HNL.Main), where X and Y represent different combinations of East Asian and Southeast Asian populations.

Table S7. Potential admixture of Bai-Yue populations inferred by *GLOBETROTTER*.
Bai-Yue populations were used as target populations to perform *GLOBETROTTER* (Hellenthal, et al. 2014) tests, using East Asian and Southeast Asian populations as donor groups. The "best.guess" gave the conclusion of admixture in each target population.

Table S8. Analysis results of *qpAdm*-based admixture modeling.

Table S9. Information of ancient samples used in this study.

Table S11. List of candidate genes under selection with adaptive variants in HNL.
Genes with adaptive variants over 99.995th percentile in PBS scan of HNL-Han-CEU trio were selected as candidate genes under selection in HNL.

Table S12. Functional categories enriched from genes with adaptive variants in HNL.
Enrichment analysis was performed using *metascape* (Zhou, et al. 2019) to investigate interaction of candidate genes under selection in HNL.

Table S13. Candidate pathways of polygenic selection in HNL.
The candidate polygenic selection was defined as a KEGG pathway (Kanehisa, et al. 2017) that show significant higher PBS distribution than the rest genes across the genome.

Table S14. List of differentiated genes within Bai-Yue lineage.

Genes with adaptive variants over 99.995^{th} percentile in PBS scan of HNL-CDX-Han and HNL-KHV-Han trios were selected as differentiated genes between island and mainland Bai-Yue populations.

References

1000 Genomes Project Consortium. 2015. A global reference for human genetic variation. Nature 526:68-74.

Alexander DH, Novembre J, Lange K. 2009. Fast model-based estimation of ancestry in unrelated individuals. Genome Res 19:1655-1664.

Bouckaert R, Heled J, Kuhnert D, Vaughan T, Wu CH, Xie D, Suchard MA, Rambaut A, Drummond AJ. 2014. BEAST 2: a software platform for Bayesian evolutionary analysis. PLoS Comput Biol 10:e1003537.

Browning SR, Browning BL. 2015. Accurate Non-parametric Estimation of Recent Effective Population Size from Segments of Identity by Descent. Am J Hum Genet 97:404-418.

Delfin F, Min-Shan Ko A, Li M, Gunnarsdottir ED, Tabbada KA, Salvador JM, Calacal GC, Sagum MS, Datar FA, Padilla SG, et al. 2014. Complete mtDNA genomes of Filipino ethnolinguistic groups: a melting pot of recent and ancient lineages in the Asia-Pacific region. Eur J Hum Genet 22:228-237.

Delfin F, Myles S, Choi Y, Hughes D, Illek R, van Oven M, Pakendorf B, Kayser M, Stoneking M. 2012. Bridging near and remote Oceania: mtDNA and NRY variation in the Solomon Islands. Mol Biol Evol 29:545-564.

Gao Y, Zhang C, Yuan L, Ling Y, Wang X, Liu C, Pan Y, Zhang X, Ma X, Wang Y, et al. 2020. PGG.Han: the Han Chinese genome database and analysis platform. Nucleic Acids Res 48:D971-D976.

GTEx Consortium. 2013. The Genotype-Tissue Expression (GTEx) project. Nat Genet 45:580-585.

Hammer MF, Karafet TM, Park H, Omoto K, Harihara S, Stoneking M, Horai S. 2006. Dual origins of the Japanese: common ground for hunter-gatherer and farmer Y chromosomes. J Hum Genet 51:47-58.

He G, Wang Z, Guo J, Wang M, Zou X, Tang R, Liu J, Zhang H, Li Y, Hu R, et al. 2020. Inferring the population history of Tai-Kadai-speaking people and southernmost Han Chinese on Hainan Island by genome-wide array genotyping. Eur J Hum Genet 28:1111-1123.

Hellenthal G, Busby GBJ, Band G, Wilson JF, Capelli C, Falush D, Myers S. 2014. A genetic atlas of human admixture history. Science 343:747-751.

Hill C, Soares P, Mormina M, Macaulay V, Clarke D, Blumbach PB, Vizuete-Forster M, Forster P, Bulbeck D, Oppenheimer S, et al. 2007. A mitochondrial stratigraphy for island southeast Asia. Am J Hum Genet 80:29-43.

Jin HJ, Tyler-Smith C, Kim W. 2009. The peopling of Korea revealed by analyses of mitochondrial DNA and Y-chromosomal markers. PLoS One 4:e4210.

Kanehisa M, Furumichi M, Tanabe M, Sato Y, Morishima K. 2017. KEGG: new perspectives on genomes, pathways, diseases and drugs. Nucleic Acids Res 45:D353-D361.

Ko AMS, Chen CY, Fu QM, Delfin F, Li MK, Chiu HL, Stoneking M, Ko YC. 2014. Early Austronesians: Into and Out Of Taiwan. Am J Hum Genet 94:426-436.

Kong QP, Yao YG, Liu M, Shen SP, Chen C, Zhu CL, Palanichamy MG, Zhang YP. 2003. Mitochondrial DNA sequence polymorphisms of five ethnic populations from northern China. Hum Genet 113:391-405.

Leigh JW, Bryant D. 2015. POPART: full-feature software for haplotype network construction. Methods Ecol Evol 6:1110-1116.

Li B, Zhong F, Yi H, Wang X, Li L, Wang L, Qi X, Wu L. 2007. Genetic polymorphism of mitochondrial DNA in Dong, Gelao, Tujia, and Yi ethnic populations from Guizhou, China. J Genet Genomics 34:800-810.

Li D, Sun Y, Lu Y, Mustavich LF, Ou C, Zhou Z, Li S, Jin L, Li H. 2010. Genetic origin of Kadai-speaking Gelong people on Hainan island viewed from Y chromosomes. J Hum Genet 55:462-468.

Li H, Cai X, Winograd-Cort ER, Wen B, Cheng X, Qin Z, Liu W, Liu Y, Pan S, Qian J, et al. 2007. Mitochondrial DNA diversity and population differentiation in southern East Asia. Am J Phys Anthropol 134:481-488.

Lipson M, Cheronet O, Mallick S, Rohland N, Oxenham M, Pietrusewsky M, Pryce TO, Willis A, Matsumura H, Buckley H, et al. 2018. Ancient genomes document multiple waves of migration in Southeast Asian prehistory. Science 361:92-95.

Lu D, Lou H, Yuan K, Wang X, Wang Y, Zhang C, Lu Y, Yang X, Deng L, Zhou Y, et al. 2016. Ancestral Origins and Genetic History of Tibetan Highlanders. Am J Hum Genet 99:580-594.

Ma X, Yang W, Gao Y, Pan Y, Lu Y, Chen H, Lu D, Xu S. 2021. Genetic Origins and Sex-Biased Admixture of the Huis. Mol Biol Evol 38:3804-3819.

Mallick S, Li H, Lipson M, Mathieson I, Gymrek M, Racimo F, Zhao M, Chennagiri N, Nordenfelt S, Tandon A, et al. 2016. The Simons Genome Diversity Project: 300 genomes from 142 diverse populations. Nature 538:201-206.

Manichaikul A, Mychaleckyj JC, Rich SS, Daly K, Sale M, Chen WM. 2010. Robust relationship inference in genome-wide association studies. Bioinformatics 26:2867-2873.

Maples BK, Gravel S, Kenny EE, Bustamante CD. 2013. RFMix: a discriminative modeling approach for rapid and robust local-ancestry inference. Am J Hum Genet 93:278-288.

McLaren W, Gil L, Hunt SE, Riat HS, Ritchie GR, Thormann A, Flicek P, Cunningham F. 2016. The Ensembl Variant Effect Predictor. Genome Biol 17:122.

Meyer M, Kircher M, Gansauge MT, Li H, Racimo F, Mallick S, Schraiber JG, Jay F, Prufer K, de Filippo C, et al. 2012. A high-coverage genome sequence from an archaic Denisovan individual. Science 338:222-226.

Morseburg A, Pagani L, Ricaut FX, Yngvadottir B, Harney E, Castillo C, Hoogervorst T, Antao T, Kusuma P, Brucato N, et al. 2016. Multi-layered population structure in Island Southeast Asians. Eur J Hum Genet 24:1605-1611.

Poznik GD, Xue Y, Mendez FL, Willems TF, Massaia A, Wilson Sayres MA, Ayub Q, McCarthy SA, Narechania A, Kashin S, et al. 2016. Punctuated bursts in human male demography inferred from 1,244 worldwide Y-chromosome sequences. Nat Genet 48:593-599.

Prufer K, Racimo F, Patterson N, Jay F, Sankararaman S, Sawyer S, Heinze A, Renaud G, Sudmant PH, de Filippo C, et al. 2014. The complete genome sequence of a Neanderthal from the Altai Mountains. Nature 505:43-49.

Schiffels S, Durbin R. 2014. Inferring human population size and separation history from multiple genome sequences. Nat Genet 46:919-925.

Sherry ST, Ward MH, Kholodov M, Baker J, Phan L, Smigielski EM, Sirotkin K. 2001. dbSNP: the NCBI database of genetic variation. Nucleic Acids Res 29:308-311.

Song MY, Wang Z, Zhang YQ, Zhao CX, Lang M, Xie MK, Qian XQ, Wang MG, Hou YP. 2019. Forensic characteristics and phylogenetic analysis of both Y-STR and Y-SNP in the Li and Han ethnic groups from Hainan Island of China. Forensic Sci Int-Gen 39:E14-E20.
Speidel L, Forest M, Shi S, Myers SR. 2019. A method for genome-wide genealogy estimation for thousands of samples. Nat Genet 51:1321-1329.
Trejaut JA, Poloni ES, Yen JC, Lai YH, Loo JH, Lee CL, He CL, Lin M. 2014. Taiwan Y-chromosomal DNA variation and its relationship with Island Southeast Asia. BMC Genet 15:77.
Tucci S, Vohr SH, McCoy RC, Vernot B, Robinson MR, Barbieri C, Nelson BJ, Fu W, Purnomo GA, Sudoyo H, et al. 2018. Evolutionary history and adaptation of a human pygmy population of Flores Island, Indonesia. Science 361:511-516.
Wang CC, Yeh HY, Popov AN, Zhang HQ, Matsumura H, Sirak K, Cheronet O, Kovalev A, Rohland N, Kim AM, et al. 2021. Genomic insights into the formation of human populations in East Asia. Nature 591:413-+.
Wang TY, Wang W, Xie GM, Li Z, Fan XC, Yang QP, Wu XC, Cao P, Liu YC, Yang RW, et al. 2021. Human population history at the crossroads of East and Southeast Asia since 11,000 years ago. Cell 184:3829-+.
Wen B, Li H, Gao S, Mao XY, Gao Y, Li F, Zhang F, He YG, Dong YL, Zhang YJ, et al. 2005. Genetic structure of Hmong-Mien speaking populations in East Asia as revealed by mtDNA lineages. Mol Biol Evol 22:725-734.
Yang MA, Fan XC, Sun B, Chen CY, Lang JF, Ko YC, Tsang CH, Chiu HL, Wang TY, Bao QC, et al. 2020. Ancient DNA indicates human population shifts and admixture in northern and southern China. Science 369:282-+.
Yuan K, Ni X, Liu C, Pan Y, Deng L, Zhang R, Gao Y, Ge X, Liu J, Ma X, et al. 2021. Refining models of archaic admixture in Eurasia with ArchaicSeeker 2.0. Nat Commun 12:6232.
Zhang C, Gao Y, Ning Z, Lu Y, Zhang X, Liu J, Xie B, Xue Z, Wang X, Yuan K, et al. 2019. PGG.SNV: understanding the evolutionary and medical implications of human single nucleotide variations in diverse populations. Genome Biol 20:215.
Zhao Q, Pan SL, Qin ZD, Cai XY, Lu Y, Farina SE, Liu CW, Peng JH, Xu JS, Yin RX, et al. 2010. Gene flow between Zhuang and Han populations in the China-Vietnam borderland. J Hum Genet 55:774-776.
Zhou Y, Browning SR, Browning BL. 2020. A Fast and Simple Method for Detecting Identity-by-Descent Segments in Large-Scale Data. Am J Hum Genet 106:426-437.
Zhou Y, Zhou B, Pache L, Chang M, Khodabakhshi AH, Tanaseichuk O, Benner C, Chanda SK. 2019. Metascape provides a biologist-oriented resource for the analysis of systems-level datasets. Nat Commun 10:1523.
